# Enzymatic Macrocyclization of 1,2,3‐Triazole Peptide Mimetics

**DOI:** 10.1002/anie.201601564

**Published:** 2016-04-05

**Authors:** Emilia Oueis, Marcel Jaspars, Nicholas J. Westwood, James H. Naismith

**Affiliations:** ^1^ Biomedical Science Research ComplexUniversity of St Andrews, BSRCNorth HaughSt AndrewsKY16 9STUK; ^2^ State Key Laboratory of BiotherapySichuan UniversityChina; ^3^ Marine Biodiscovery Centre, Department of ChemistryUniversity of AberdeenOld AberdeenAB24 3UEUK

**Keywords:** biotransformation, cyanobactin, cyclic peptides, peptidomimetics, triazole

## Abstract

The macrocyclization of linear peptides is very often accompanied by significant improvements in their stability and biological activity. Many strategies are available for their chemical macrocyclization, however, enzyme‐mediated methods remain of great interest in terms of synthetic utility. To date, known macrocyclization enzymes have been shown to be active on both peptide and protein substrates. Here we show that the macrocyclization enzyme of the cyanobactin family, PatGmac, is capable of macrocyclizing substrates with one, two, or three 1,4‐substituted 1,2,3‐triazole moieties. The introduction of non‐peptidic scaffolds into macrocycles is highly desirable in tuning the activity and physical properties of peptidic macrocycles. We have isolated and fully characterized nine non‐natural triazole‐containing cyclic peptides, a further ten molecules are also synthesized. PatGmac has now been shown to be an effective and versatile tool for the ring closure by peptide bond formation.

Macrocycles in general and peptidic macrocycles in particular have recently gained more attention since they have been identified as potential sources for novel therapeutics.^1^ These compounds, abundant in nature, are known to possess a wide range of biological activities.^1^, ^2^ They owe their attractiveness to having higher structural rigidity and better chemical stability, especially against hydrolysis and peptidase activity in the case of peptide‐based macrocycles.^3^ The increased molecular weight of these compounds is usually balanced by their increased specificity and selectivity towards the targets compared to “small” molecules.^4^ Patellamides are natural cyclic octapeptides that belong to the large family of cyanobactins.^5^ Their biosynthesis consists of the modification of a long precursor peptide via the action of different tailoring enzymes to achieve heterocyclization of cysteines, or threonines and serines to thiazoline or oxazoline, proteolysis, macrocyclisation, epimerization of certain amino acids, oxidation of the heterocycles, and in some cases prenylation.^6^

Although many natural peptidic macrocycles have some side chain modifications, (e.g. sugars, prenyl, lipid), it would appear that it is relatively rare in nature, to have both amino acids and non‐peptidic molecules forming the macrocyclic backbone. Nonetheless, the synthesis of such compounds would be greatly beneficial for complementary SAR studies on different targets.2b A restriction to the natural amino acid building blocks in these cyclic peptides limits the diversity and thus the chemical properties and activities that such molecules can possess. A partial solution to this problem comes from the use of non‐natural amino acids in biological systems in vivo or in vitro,^7^ which have already been demonstrated for peptidic macrocycles^8^ (Figure 1). Hybrid molecules have gained significant attention through the use of the so called stapled peptides,^9^ but in this case, the non‐peptidic module tends to be chosen to lock the peptide in a specific conformation rather than being a key component for activity.

**Figure 1:**
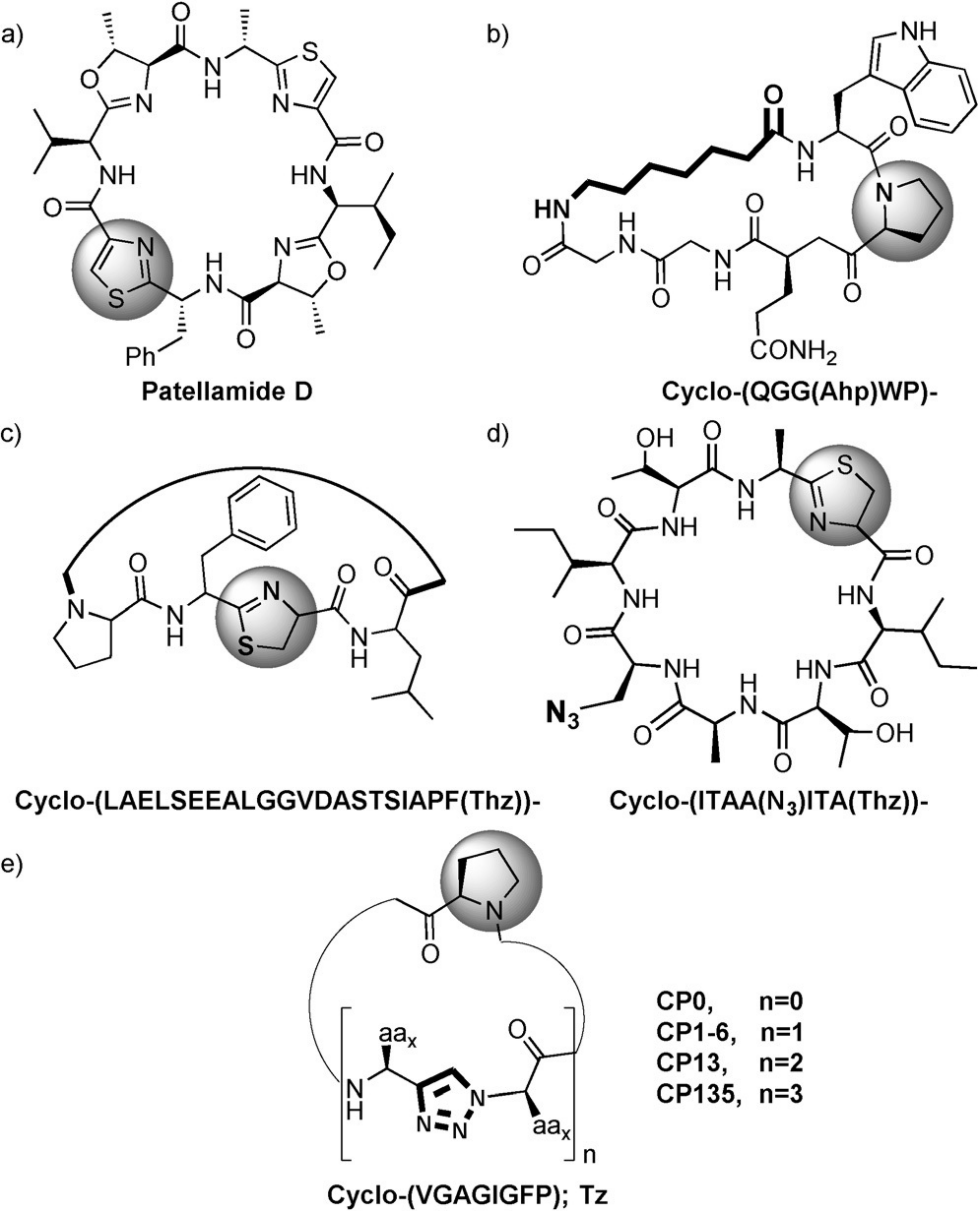
PatGmac macrocylized compounds. a) The natural compound, patellamide D. b–d) Examples of previously synthesized non‐natural cyclic compounds.8a–8c e) This study; *n* represents the number of triazole units in the sequence; the molecule number represents the position of the triazole (Tz) moiety/moieties in the core peptide (i.e. Cyclo(‐VTzGAGIGFP) is **CP1**). The highlighted heterocycle in grey is C‐terminal in the core peptide. The bold parts of the molecules represent the non‐natural modifications to the core peptide. Ahp: 7‐aminoheptanoic acid. (Thz): thiazoline heterocycle.

The ring closure of peptidic macrocycles can be achieved either chemically2b, 10 or enzymatically with a number of distinct enzymes known including butelase,^11^ POPB,^12^ sortase,^13^ recombinant asparaginyl endopeptidase (AEP),^14^ thioesterase,^15^ and the macrocyclase enzyme (PatGmac) from the patellamide family.8a–8c, 16 PatGmac recognizes a C‐terminal AYD(G) motif located outside the core of the precursor peptide substrate and cleaves it to form a water shielded acyl‐enzyme intermediate using its catalytic Asp‐His‐Ser triad.^16^ The intermediate is then attacked by the free N‐terminal amine of the core peptide. The substrate must have a thiazoline, oxazoline or a proline immediately N‐terminal to the (cleaved off) AYD(G)^16^ motif with no other restrictions on the rest of the core peptide. This very broad substrate range has made PatGmac an attractive tool for biotransformation (Figure 1). We decided to test its ability to macrocyclize substrates in which non‐peptidic motifs are included in the sequence. We have chosen to investigate the incorporation of 1,4‐substituted 1,2,3‐triazole^17^ groups into macrocycles.^18^ The triazole ring can act as an isostere for a *trans*‐amide bond and its synthesis is facile.^19^ Further the triazole moiety has been incorporated in many biologically active compounds as it is thermodynamically and physiologically stable.^20^ We reasoned therefore this would be a suitable test of the versatility of PatGmac by systematically incorporating the triazole unit (Tz) at different positions of the precursor peptide while varying their number as well. We report herein the successful formation (catalyzed by PatGmac) of macrocycles containing one, two, and three 1,2,3‐triazoles.^21^

A series of 19 different linear precursor peptides **P** based on the core sequence VGAGIGFP were prepared by standard solid phase peptide synthesis using the Fmoc strategy (see Supporting Information, SI). The linear peptides VGAGIGFPAYD‐NH_2_ consisted of the core sequence modified with one, two, or three non‐consecutive triazoles and the PatGmac recognition motif AYD with an N‐terminal amide. The triazoles were generated directly on solid phase using a copper(I) catalyzed azide‐alkyne cycloaddition (CuAAC).18b Azido acids **1 a**,**b** and **1 d** were synthesized in one step by a diazo transfer reaction^22^ (Scheme S3).^23^ Fmoc protected amino alkynes **2 a**–**c** were synthesized in five steps starting from the commercial Boc protected amino acid. First, the Weinreb amides were synthesized by a standard coupling reaction. The use of DIBAL‐H for the amide reduction in a one pot procedure with the Bestmann–Ohira reagent^24^ in our hands was found to yield very little conversion to the aldehyde. Separating the two steps with the reduction to aldehyde using LiAlH_4_ followed by the Bestmann‐Ohira reagent, successfully afforded the Boc protected amino alkynes. A final deprotection and protection step afforded the desired alkynes **2 a**–**c** in moderate yields overall (Scheme S4). The N‐terminal azide was attached on the growing peptide by a standard coupling reaction then was subjected to the CuAAC reaction with the corresponding alkyne counterpart **2** on solid phase (Scheme 1; Scheme S1). Final precursor peptides **P1‐6** have a single triazole (Tz) at a different position of the core sequence^25^ (the number denoting the position), precursors **P13‐16**, **P24‐26**, **P35‐36** and **P46** have two triazoles (i.e. at positions 1 and 3 of the peptide for **P13**; VTzGATzGIGFPAYD), and **P135** and **P246** have three triazoles (Table S1; Table S2). **P0** has no triazoles and was synthesized to serve as a control for the macrocyclization reaction.

**Scheme 1:**
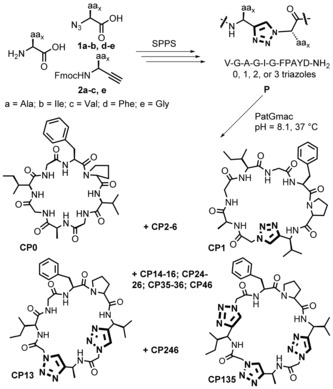
Synthesis of triazole‐containing cyclic peptides.

All precursor peptides **P** were subjected to the macrocylization reaction conditions in the presence of PatGmac in bicine buffer at pH 8.1 and 37 °C. The reaction progress was monitored by MALDI‐MS and some of the resulting spectra are shown in Figure 2.

**Figure 2:**
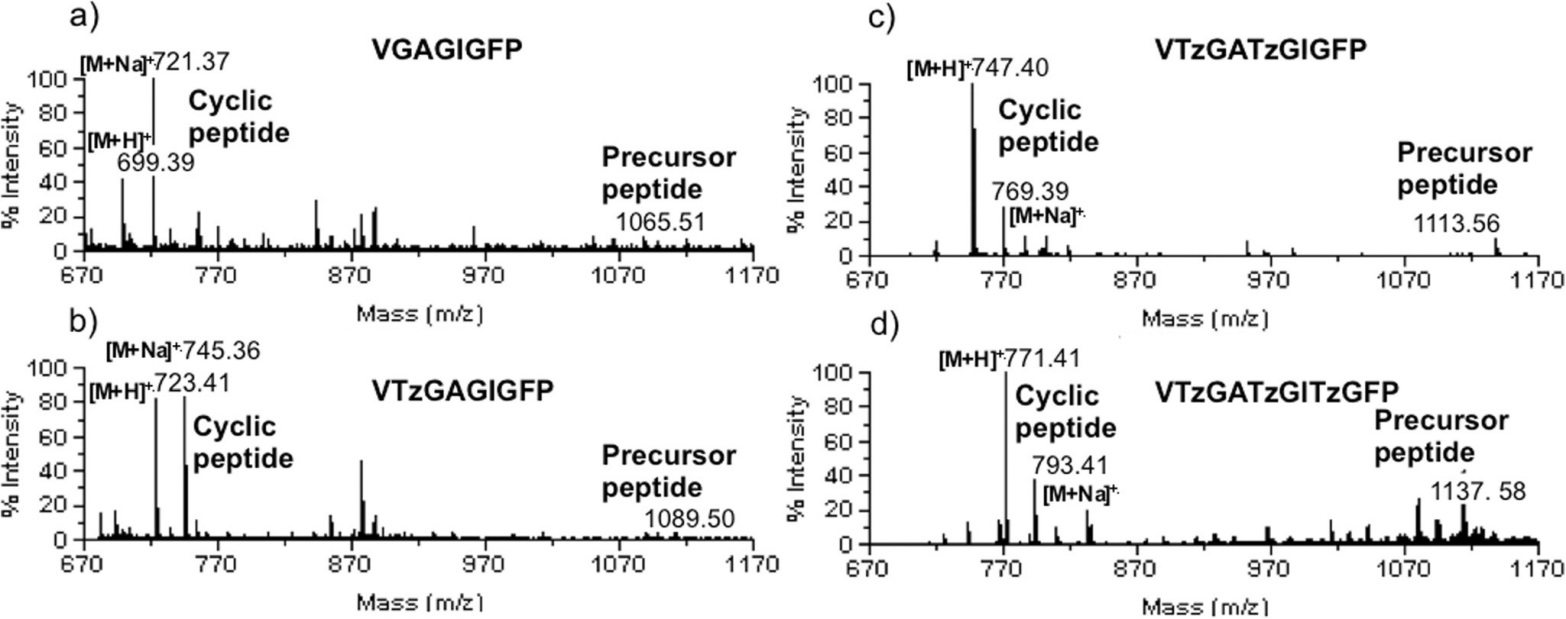
MALDI‐MS traces of the macrocyclization reactions of representative sequences with a) control peptide; VGAGIGFP, 0 triazole, 20 days; b) VTzGAGIGFP, 1 triazole, 21 days; c) VTzGATzGIGFP, 2 triazoles, 23 days; d) VTzGATzGITzGFP, 3 triazoles, 25 days.

All 19 precursor peptides converted to the desired macrocyclic structure and no significant linear product (AYD cleavage product) was detected in the reaction mixture. We observed that two triazole‐containing substrates were slower to macrocyclize than single triazole‐containing peptides and that both three triazole peptides were the slowest to convert. Nonetheless, because PatGmac is known to be a slow enzyme, the rate difference is insignificant (reactions were monitored 20 to 25 days for full conversion on large scale).^16^

Nine of the precursor peptides, **P0**, **P1**, **P2**, **P3**, **P4**, **P5**, **P6**, **P13** and **P135**, were reacted at a larger scale (8 µmol, 9–10 mg) in the presence of PatGmac under the previously stated conditions in order to provide sufficient amounts of the macrocyclic product for full characterization. Cyclic peptides **CP** were extracted and HPLC purified to afford moderate yields of high purity samples. Results are summarized in Tables 1 and S3 (see Scheme 1 or SI, section I for structures). For some of these (**CP3**, **CP13**, **CP135**), two peaks on the HPLC had the expected mass of the cyclic peptide in the crude reaction mixture, as can be seen from the ratio column in Table 1 (SI, section VIII). When applicable, both peaks were collected and analyzed by HRMS and MS‐MS fragmentation patterns and both were determined to be the desired cyclic peptide (Table S3). However, the NMR experiments were only conducted for the major isolated compound.

**Table 1:** PatGmac macrocylization reaction products.

Peptide	Core peptide ^[a]^	Ratio [%]^[b]^	Yield [%]^[c]^	Purity [%]^[d]^
**CP0**	VGAGIGFP	100	32	99
**CP1**	VTzGAGIGFP	100	43	99
**CP2**	VGTzAGIGFP	100	48	91
**CP3**	VGATzGIGFP	76/24	55	99
**CP4**	VGAGTzIGFP	100	34	98
**CP5**	VGAGITzGFP	100	58	93
**CP6**	VGAGIGTzFP	100	40	98
**CP13**	VTzGATzGIGFP	20/80	54	98
**CP135**	VTzGATzGITzGFP	15/85	39	97

[a] Tz=triazole replacing the amide bond. [b] Ratio of cyclic products in crude mixture (SI, section VIII). [c] Total yield. [d] Purity of major product determined by HPLC, UV absorption at 220 nm (SI, section IX).

The MS‐MS fragmentation data of the cyclic peptides gave a very complex series of ions consistent with their peptidic sequence, the newly formed valine to proline bond (macrocycle formation), and the triazole presence (SI, section VII).

NMR experiments were also conducted in order to further confirm their structures. The proton NMR data of the cyclic peptides showed at least two species present at different ratios depending on the compound. For **CP0**, **CP1**, **CP2**, **CP3 CP4**, **CP6** and **CP13** this was shown by Exchange Spectroscopy (EXSY) experiments to result from different conformers (SI, section VI). However, compound **CP5** was found to have two distinct compounds in a 1:0.6 ratio. The NMR assignments of both species show they have the same peptidic sequence (cyclic VGAGITzGFP). Both HRMS and MS‐MS fragmentation data confirm the presence of one mass/one sequence. This led us to believe that the two species are most likely epimers but we cannot exclude structurally rigid conformers unable to interconvert.^26^

**CP135** has a very complex proton and 2D NMR spectra that showed at least four different species (with two other minor species). General assignment has been achieved for most proton peaks (SI, section V) however, the individual sequence for each was not determined. EXSY showed that two of them are related conformers and the other two are distinct compounds. Similarly to compound **CP5**, these species would most probably arise from epimers or from conformationally rigid variants.

Biotransformation is now widely used in organic chemistry for the synthesis of biologically relevant compounds. The PatGmac enzyme is unusual in that it operates on substrates and disposes of almost the entire recognition site during the reaction. PatGmac macrocyclizes a wide range of natural peptidic substrates and tolerates non‐natural amino acids.8a,8b From a chemical diversity point of view, the ability to mix amino acid residues and non‐amino acid scaffolds within the backbone of macrocyles is highly desirable. We have now shown that it is possible to synthesize macrocycles with one, two, or three triazoles within a peptidic sequence. Thus PatGmac is able to process peptide hybrid substrates extending its utility. The ability to macrocyclise such hybrid molecules highlights the scope for creating highly diverse libraries through split and pool approaches.

## Supporting information

As a service to our authors and readers, this journal provides supporting information supplied by the authors. Such materials are peer reviewed and may be re‐organized for online delivery, but are not copy‐edited or typeset. Technical support issues arising from supporting information (other than missing files) should be addressed to the authors.

Supplementary

## References

[1a] E. M. Driggers , S. P. Hale , J. Lee , N. K. Terrett , Nat. Rev. Drug Discovery 2008, 7, 608; 18591981 10.1038/nrd2590

[1b] D. J. Newman , G. M. Cragg in Macrocycles in Drug Discovery (Eds.: J. R. Morphy, C. J. Harris), The Royal Society of Chemistry, London, 2015, pp. 1–36.

[2a] A. T. Bockus , C. M. McEwen , R. S. Lokey , Curr. Top. Med. Chem. 2013, 13, 821; 23578026 10.2174/1568026611313070005

[2b] X. Yu , D. Sun , Molecules 2013, 18, 6230. 23708234 10.3390/molecules18066230 PMC4374646

[3] S. H. Joo , Biomol. Ther. 2012, 20, 19. 10.4062/biomolther.2012.20.1.019 PMC3792197 24116270

[4] C. Heinis , Nat. Chem. Biol. 2014, 10, 696. 25038789 10.1038/nchembio.1605

[5] M. Jaspars , Chem. Commun. 2014, 50, 10174–10176. 10.1039/c3cc49252d 25068542

[6a] J. Koehnke , A. F. Bent , W. E. Houssen , G. Mann , M. Jaspars , J. H. Naismith , Curr. Opin. Struct. Biol. 2014, 29, 112–121; 25460274 10.1016/j.sbi.2014.10.006 PMC4268494

[6b] E. W. Schmidt , J. T. Nelson , D. A. Rasko , S. Sudek , J. A. Eisen , M. G. Haygood , J. Ravel , Proc. Natl. Acad. Sci. USA 2005, 102, 7315–7320. 15883371 10.1073/pnas.0501424102 PMC1091749

[7] T. S. Young , P. G. Schultz , J. Biol. Chem. 2010, 285, 11039–11044. 20147747 10.1074/jbc.R109.091306 PMC2856976

[8a] J. A. McIntosh , C. R. Robertson , V. Agarwal , S. K. Nair , G. W. Bulaj , E. W. Schmidt , J. Am. Chem. Soc. 2010, 132, 15499–15501; 20961047 10.1021/ja1067806 PMC2975588

[8b] E. Oueis , C. Adamson , G. Mann , H. Ludewig , P. Redpath , M. Migaud , N. J. Westwood , J. H. Naismith , ChemBioChem 2015, 16, 2646–2650; 26507241 10.1002/cbic.201500494 PMC4736454

[8c] D. Sardar , Z. Lin , E. W. Schmidt , Chem. Biol. 2015, 22, 907–916; 26165156 10.1016/j.chembiol.2015.06.014 PMC4522240

[8d] T. Kawakami , A. Ohta , M. Ohuchi , H. Ashigai , H. Murakami , H. Suga , Nat. Chem. Biol. 2009, 5, 888–890. 19915537 10.1038/nchembio.259

[9] A. K. Yudin , Chem. Sci. 2015, 6, 30–49. 10.1039/c4sc03089c PMC5424464 28553456

[10] C. J. White , A. K. Yudin , Nat. Chem. 2011, 3, 509–524. 21697871 10.1038/nchem.1062

[11] G. K. T. Nguyen , A. Kam , S. Loo , A. E. Jansson , L. X. Pan , J. P. Tam , J. Am. Chem. Soc. 2015, 137, 15398–15401. 26633100 10.1021/jacs.5b11014

[12] H. Luo , S.-Y. Hong , R. M. Sgambelluri , E. Angelos , X. Li , J. D. Walton , Chem. Biol. 2014, 21, 1610–1617. 25484237 10.1016/j.chembiol.2014.10.015 PMC4272623

[13] W. van't Hof , H. M. Silvie , C. I. V. Enno , G. M. B. Jan , Biol. Chem. 2015, 396, 283–293. 25581753 10.1515/hsz-2014-0260

[14] K. S. Harris , T. Durek , Q. Kaas , A. G. Poth , E. K. Gilding , B. F. Conlan , I. Saska , N. L. Daly , N. L. van der Weerden , D. J. Craik , M. A. Anderson , Nat. Commun. 2015, 6, 6370. 26680698 10.1038/ncomms10199 PMC4703859

[15] R. M. Kohli , J. W. Trauger , D. Schwarzer , M. A. Marahiel , C. T. Walsh , Biochemistry 2001, 40, 7099–7108. 11401555 10.1021/bi010036j

[16] J. Koehnke , A. Bent , W. E. Houssen , D. Zollman , F. Morawitz , S. Shirran , J. Vendome , A. F. Nneoyiegbe , L. Trembleau , C. H. Botting , M. C. M. Smith , M. Jaspars , J. H. Naismith , Nat. Struct. Mol. Biol. 2012, 19, 767–772. 22796963 10.1038/nsmb.2340 PMC3462482

[17] In the rest of the paper, the 1,4-substituted 1,2,3-triazole will be referred to as triazole.

[18a] C. W. Tornøe , C. Christensen , M. Meldal , J. Org. Chem. 2002, 67, 3057–3064; 11975567 10.1021/jo011148j

[18b] M. Meldal , C. W. Tornøe , Chem. Rev. 2008, 108, 2952–3015. 18698735 10.1021/cr0783479

[19] G. C. Tron , T. Pirali , R. A. Billington , P. L. Canonico , G. Sorba , A. A. Genazzani , Med. Res. Rev. 2008, 28, 278–308. 17763363 10.1002/med.20107

[20] I. Pibiri , S. Buscemi , Curr. Bioact. Compd. 2010, 6, 208.

[21] The replacement of an amide bond by a triazole is represented by a “Tz” between the corresponding two amino acids in the sequence.

[22] E. D. Goddard-Borger , R. V. Stick , Org. Lett. 2007, 9, 3797. 17713918 10.1021/ol701581g

[23] Reagant **1 e** is commercially available. Reagant **2 e** is easily prepared from commercial propargylamine.

[24] H. D. Dickson , S. C. Smith , K. W. Hinkle , Tetrahedron Lett. 2004, 45, 5597–5599.

[25] The molecule number represents the position of the triazole (Tz) moiety/moieties in the core peptide (VTzG is position 1, GTzF is position 6).

[26] J. N. Tabudravu , M. Jaspars , L. A. Morris , J. J. Kettenes-van den Bosch , N. Smith , J. Org. Chem. 2002, 67, 8593–8601. 12444643 10.1021/jo020482s

