# Supplementary material for: Enzymatic Macrocyclization of 1,2,3‐Triazole Peptide Mimetics
Source: Angew Chem Int Ed Engl. 2016 Apr 5;55(19):5842–5. doi: 10.1002/anie.201601564 (PMC4924597; doi:10.1002/anie.201601564)
Supplement: Supplementary file 1 — Supplementary [file ANIE-55-5842-s001.pdf]

Angewandte Chemie
Eine Zeitschrift der Gesellschaft Deutscher Chemiker

Supporting Information

Enzymatic Macrocyclization of 1,2,3-Triazole Peptide Mimetics

*Emilia Oueis, Marcel Jaspars, Nicholas J. Westwood, and James H. Naismith**

anie_201601564_sm_miscellaneous_information.pdf

I. STRUCTURES OF PRECURSOR PEPTIDES P AND CYCLIC PEPTIDES CP................ S2

II. GENERAL INFORMATION AND MATERIALS S4

III. GENERAL PROCEDURES S6

IV. MS AND HPLC DATA OF STARTING AND FINAL CYCLIC PEPTIDES...................S14

V. NMR DATA OF FINAL CYCLIC PEPTIDES S16

VI. NMR SPECTRA OF FINAL CYCLIC PEPTIDES S33

VII. EXSY NMR OF FINAL CYCLIC PEPTIDES S42

VIII. MS-MS DATA OF FINAL CYCLIC PEPTIDES..S47

IX. LC-MS TRACES OF CRUDE MIXTURES OF CP3, CP13, AND CP135 ....S52

X. LC-MS TRACES OF FINAL CYCLIC PEPTIDES S55

XI. LC-MS TRACES OF STARTING PEPTIDES..S59

XII. REFERENCES S69

I. Structures of precursor peptides P and cyclic peptides CP

1. Precursor peptides P

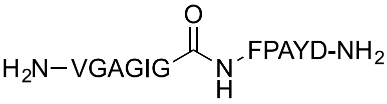

P0

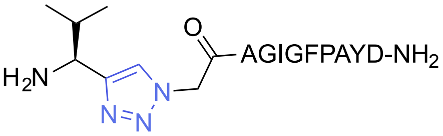

P1

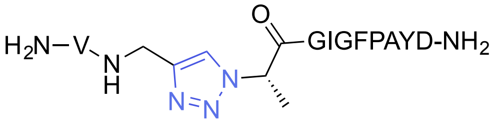

P2

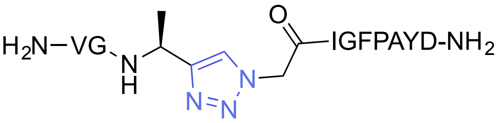

P3

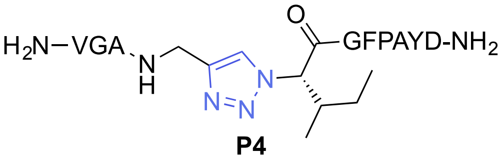

P4

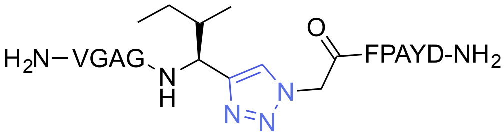

P5

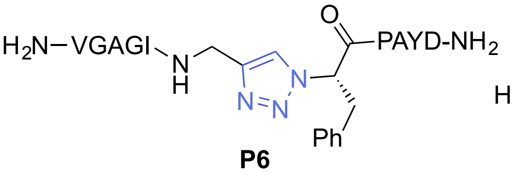

P6

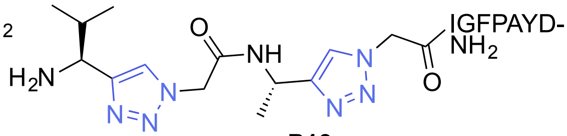

P13

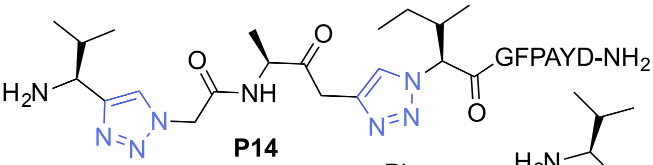

P14

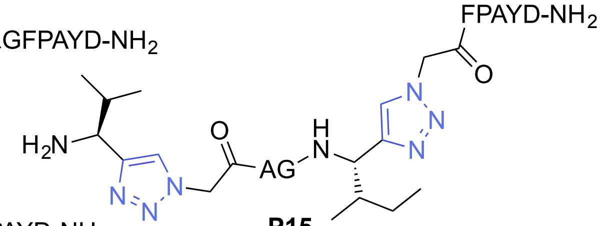

P15

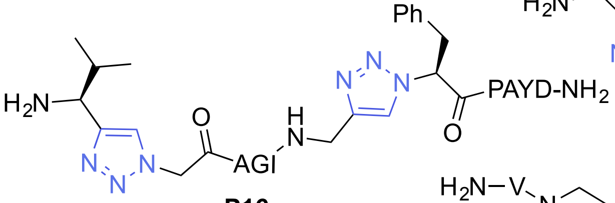

P16

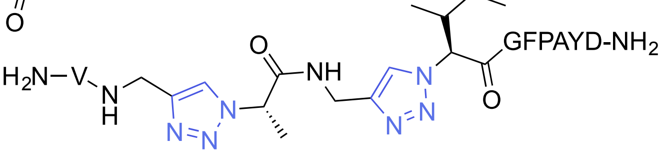

P24

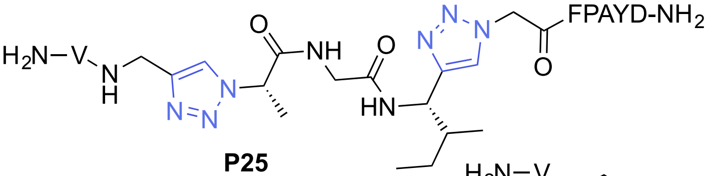

P25

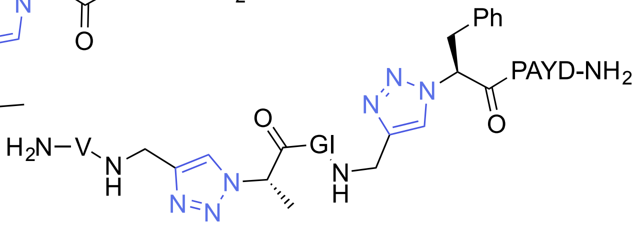

P26

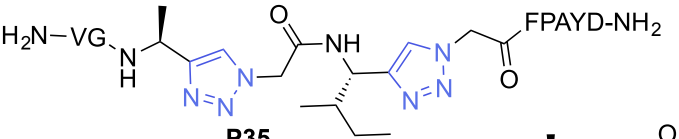

P35

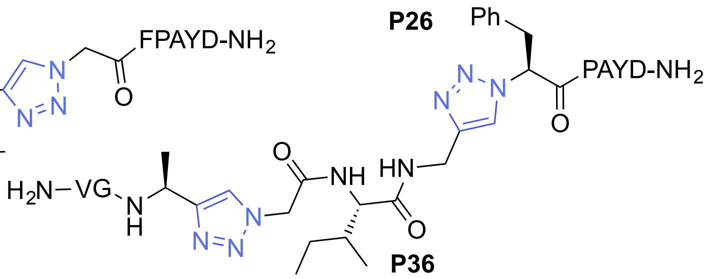

P36

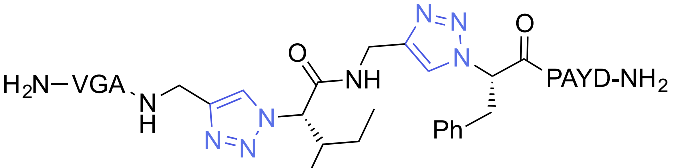

P46

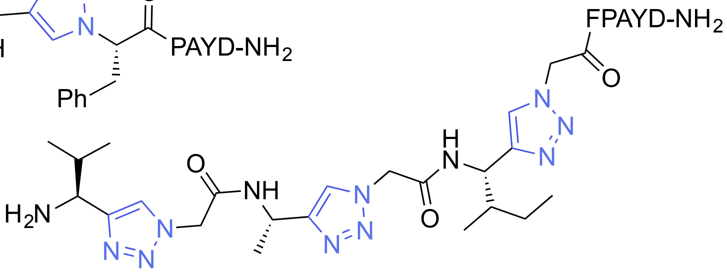

P135

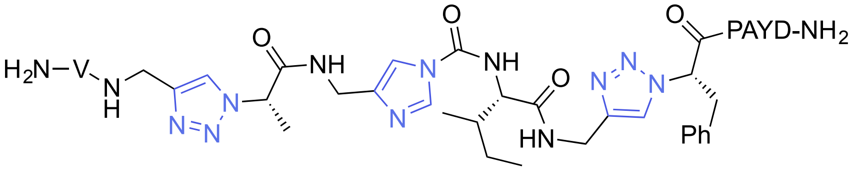

P246

2. Cyclic peptides CP

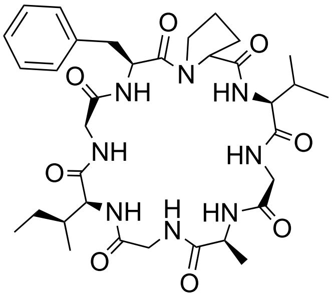

CP0

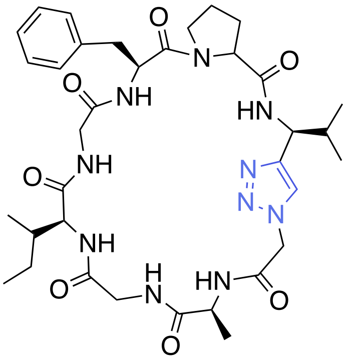

CP1

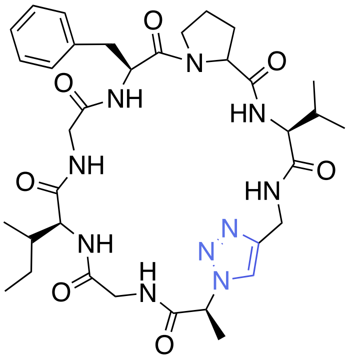

CP2

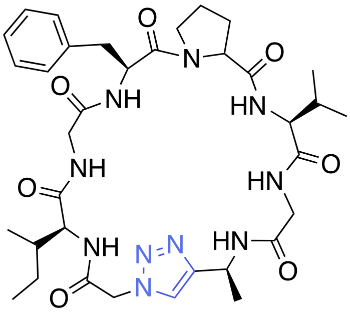

CP3

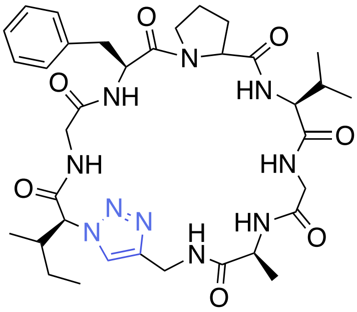

CP4

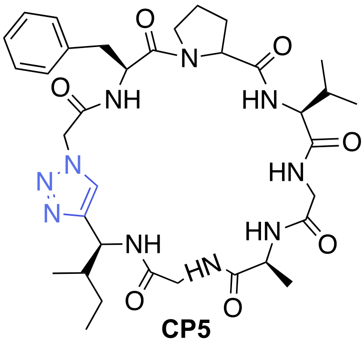

CP5

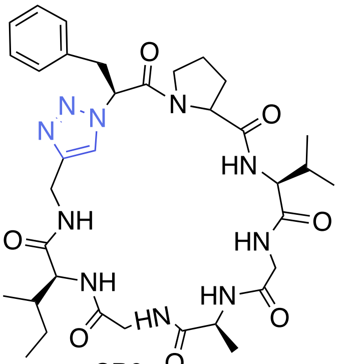

CP6

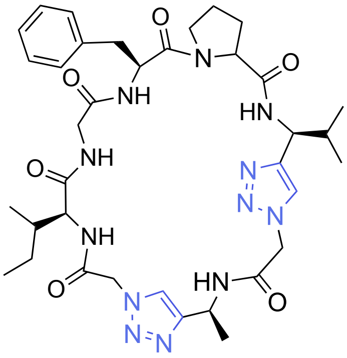

CP13

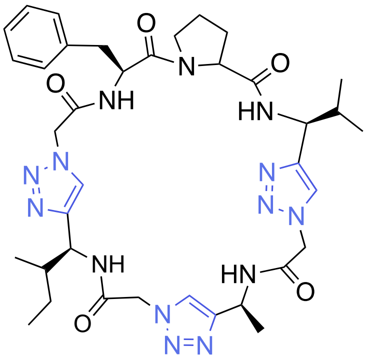

CP135

II. General information and materials

The HPLC grade acetonitrile (MeCN) was purchased from Fisher. Aqueous buffers and aqueous mobile-phases for HPLC were prepared using water purified with an Elga® Purelab® Milli-Q water purification system (purified to 18.2 MΩ.cm). DMSO, THF and dichloromethane (CH_2Cl_2) were purchased from Fisher. Amino acids and coupling reagents were purchased commercially from different sources. Other chemicals and reagents were purchased from Sigma and used without any further purification.

NMR spectra (^{1}H, 2D) were recorded on a Bruker Ascend 700 spectrometer {δH (700 MHz), δC (175 MHz)} at ambient temperature in the deuterated solvent stated. Chemical shifts are expressed in parts per million (ppm) from DMSO-d6 (δH = 2.50).[1] Multiplicities are described as s (singlet), d (doublet), q (quadruplet), qt (quintuplet), dd (doublet of doublets), t (triplet), m (multiplet), br (broad). Coupling constants *J* are quoted in Hertz (Hz) to the nearest 0.1 Hz. Signals of protons and carbons were assigned, as far as possible, by using the following two dimensional NMR spectroscopy techniques: [^{1}H, ^{1}H] COSY (Correlation Spectroscopy), [^{1}H, ^{1}H] TOCSY (Total Correlation Spectroscopy), [^{1}H, ^{1}H] ROESY (Rotating-frame NOE Spectroscopy), [^{1}H, ^{13}C] HSQC (Heteronuclear Single Quantum Coherence) and long range [^{1}H, ^{13}C] HMBC (Heteronuclear Multiple Bond Connectivity). EXSY (Exchange Spectroscopy) experiment was used to identify equilibrium chemical exchange either at rt or with heating.

Low-resolution mass spectra were obtained with an Agilent 6130 single quad apparatus equipped with an electrospray ionization source. High-resolution mass spectra (HRMS) were obtained with a Thermo Exactive Orbitrap mass spectrometer.

Reactions performed in the enzymatic media were monitored using MALDI-MS acquired using a 4800 MALDI TOF/TOF Analyser (ABSciex, Foster City, CA) equipped with a Nd:YAG 355 nm laser and calibrated using a mixture of peptides. The spot was analysed in positive MS mode between 500 and 4000 m/z, by averaging 1000 laser spots. The samples, diluted in water to reduce the buffer concentration, (0.5 µL) were applied to the MALDI target along with alpha-cyano-4-hydroxycinnamic acid matrix (0.5 µL, 10 mg/mL in 50:50 acetonitrile:0.1% TFA) and allowed to dry. MSMS data were acquired using a TripleTOF 5600+. The sample was

subjected to chromatography on an Acclaim PepMap 100 C18 trap and an Acclaim PepMap RSLC C18 column (ThermoFisher Scientific), using a nano-LC Ultra 2D plus loading pump and nano-LC as-2 autosampler (Eksigent). The sample was injected at neutral pH to avoid acid catalyzed ring opening. The trap was washed with 2% acetonitrile, 0.05% trifluoroacetic acid, and the patellamide was then eluted with a gradient of increasing acetonitrile, containing 0.1 % formic acid (15-40% acetonitrile in 5 min, 40-95% in a further 1 min, followed by 95% acetonitrile to clean the column, before re-equilibration to 15% acetonitrile). The eluent was sprayed into a TripleTOF 5600+ electrospray tandem mass spectrometer (Sciex) operating with standard nanospray conditions, and analyzed in Product Ion Scan mode isolating the m/z of interest. The collision energy was adjusted to give optimal fragmentation. The MSMS fragmentation pattern was interrogated for diagnostic peaks.

Automated solid-phase peptide synthesis (SPPS) was accomplished on a Biotage® Syro Wave™ system in polypropylene (PP) syringe with a PTFE frit. Manual SPPS was performed using a vacuum manifold and a shaker. Final cleavage and deprotection were completed manually.

Analytical RP-HPLC was performed on an Agilent infinity 1260 series equipped with either a VWD or an MWD detector and a single quadrupole MS using a Macherey-Nagel Nucleodur C18 column (10 µm x 4.6 x 250 mm) using the following chromatographic system: 1 mL/min flow rate with MeCN and 0.1 % aqueous TFA [95% TFA (5 min), linear gradient from 5 to 95% of MeCN (35 min), 95% MeCN (40 min)] and UV detection at 220 nm.

Semi-preparative RP-HPLC was performed on an Agilent infinity 1260 series equipped with a VWD or an MWD detector using a Macherey-Nagel Nucleodur C18 column (10 µm x 10 x 250 mm at 4 mL/min, a 10 µm x 16 x 250 mm at 10 mL/min, or 10 µm x 21 x 250 mm at 16 mL/min). Several chromatographic systems were used; System Pa: MeCN and 0.1 % aqueous TFA [95% TFA (5 min), linear gradient from 5 to 95% of MeCN (35 min), 95% MeCN (40 min)] and UV detection at 220 nm. System Pb: MeCN and 0.1 % aqueous TFA [95% TFA (5 min), linear gradient from 5 to x-3% of MeCN (1% /min), x+12% MeCN (0.1% /min)] and UV detection at 220 nm; where x is the % of MeCN at which the corresponding peak elutes on the analytical run of a similar column type. The runs were stopped as soon as the peptide eluted off the column.

III. General procedures

***Pat*G$_{mac}$ cloning, expression and purification:**

The *Pat*G$_{mac}$ enzyme was cloned from genomic DNA *(Prochlon sp.*) into the pHISTEV vector, expressed in *Escherichia coli* BL21 (DE3) cells grown on autoinduction medium, and purified as previously described by Koehnke *et al.*.[2] However, subsequent to the Nickel column eluting with 250 mM imidazole, the remaining purification steps were replaced with dialysis in a bicine buffered solution [20 mM Bicine, 150 mM NaCl, pH = 8.1] to remove the imidazole and the reducing agent.

Solid-phase peptide synthesis

The different precursor peptides were synthesized on solid-phase (SPPS) on a Rink amide resin (RAR) using the Fmoc strategy and Fmoc-protected amino acids (aa). Both automatic (A) and manual (M) procedures were employed. The introduction of the triazole moiety was accomplished by first reacting azido acids to the growing peptide (A or M) and then by manually introducing the triazole through a copper-catalyzed azide alkyne cycloaddition (CuAAC) between the azide-terminal peptide and the Fmoc-protected alkyne amino acid analogue (Scheme S1).

1- Resin preparation

The rink amide resin (RAR) is weighed into the fritted PP-syringe then swelled in DMF for half an hour.

2- Automated SPPS

A double coupling strategy was used for all amino acids using the following conditions for each step:

5 eq. HBTU in DMF; 8 eq. DIEA in NMP; 5 eq. aa in DMF Reaction time of 30 min with mixing/shaking for 15 s every two minutes at rt followed by 4x DMF washing cycles.

Fmoc deprotection is done in 40% piperidine/DMF in two steps:

3 min reaction with mixing/shaking for 10 s every minute at rt then 12 min reaction with mixing/shaking for 10 s every two minutes at rt followed by 6x DMF washing cycles.

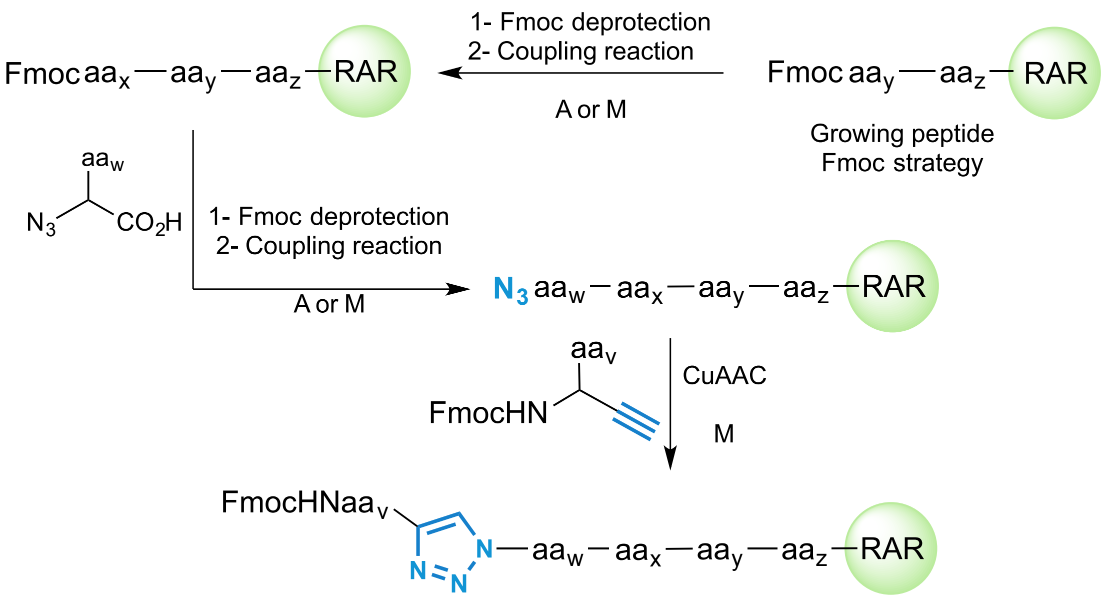

Scheme S1: General SPPS strategy for the synthesis of triazole-containing peptides.

3- Manual SPPS

A double coupling strategy was used for all amino acids using the following conditions for each step:

5 eq. HBTU in DMF; 10 eq. DIEA in NMP; 5 eq. aa in DMF Reaction time of 1h 30 min with shaking at rt followed by 4x DMF, 4x CH_2Cl_2, and 4x DMF washing cycles. Fmoc deprotection is done in 20% piperidine/DMF for 10 min followed by 4x DMF, 4x CH_2Cl_2, and 4x DMF washing cycles.

4- Manual CuAAC reaction; solid phase (SP)

After the coupling of an azido acid to the growing peptidic chain (A or M), the resin is washed with 5x THF. The corresponding amino alkyne (5 eq.) is diluted in THF and added to the resin. DIEA (10 eq.) is then added followed by CuI (5 eq.). The mixture is bubbled with dry nitrogen for 1 min then sealed, protected from light, and left overnight with shaking. After the reaction is complete (mini work up of a few

beads; repeat the procedure if reaction not complete), the resin is washed with 4x THF, 4x H_2O, 4x THF, and 4x DMSO. The resin is swelled in DMSO for 10 min before the Fmoc deprotection step (if needed) with 20% piperidine/DMF for 10 min followed by 4x DMF, 4x CH_2Cl_2, and 4x DMF washing cycles.

5- Final cleavage

The resin is washed with CH_2Cl_2 and dried for a minute on the vacuum manifold. The beads are then transferred into a flacon tube and the cleavage cocktail is added: 95% TFA, 3.5% H_2O, 1.5% TIS. The mixture is vortexed to mix everything and the flacon tube is left on the shaker for 2h. The resin is then filtered, washed with 3x CH_2Cl_2 and the filtrate is evaporated under vacuum until a few milliliters of TFA are left (< 2mL).

6- Peptide precipitation and HPLC purification

To a falcon tube containing cold diethyl ether, the remaining TFA solution is added dropwise. The peptide precipitates upon contact with the cold ether. The falcon tube is placed for 5 min in the cold and centrifuged at 4 °C and 4000 rpm for 10 min. The supernatant is decanted and the remaining solid left a few minutes under nitrogen flow to dry. The crude is then solubilized in a minimum volume of 20% acetic acid and MeCN (ultrasonic bath helps) and immediately purified by HPLC.
Peptides **P0**, **P13**, **P14**, **P15**, **P24**, **P25**, **P26**, **P35**, **P36**, **P46**, **P135**, and **P246** were purified using system Pa and peptides **P1**, **P2**, **P3**, **P4**, **P5**, **P6**, and **P16** were purified using system Pb.

Pat Gmac macrocylisation reaction

The reactions are conducted in 20 mM bicine buffer, 500 mM NaCl, and 5% DMSO solution, pH = 8.1 and incubated at 37 °C (without shaking) until full consumption of the starting peptide (MALDI monitoring). The reaction set-ups are prepared in the following order; final concentrations:

1- A solution of the linear peptide in DMSO (between 10 and 50 mM); 100 μM
2- DMSO; 5%
3- 20 mM Bicine, 150 mM NaCl, pH=8.1 buffer

4- 5 mM NaCl; 500 mM

5- *Pat*G_{mac} enzyme; 60 µM

The large scale reaction mixture is then extracted 3 times with *n*-butanol (BuOH): H_2O (1/1, v/v). BuOH is added to the aqueous reaction, vigorously mixed, and then centrifuged for 10 min at high speed to help separate the two phases. The combined BuOH fractions are evaporated under reduced pressure to dryness. The crude is solubilized in a minimum volume of H_2O/MeCN and immediately purified by HPLC using system Pa.

Synthesis of amino acids analogues

1- Synthesis of imidazole-1-sulfonyl azide hydrochloride

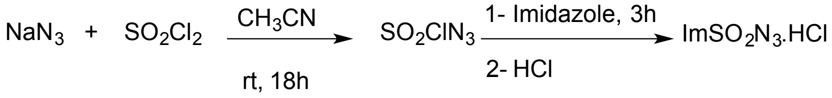

Scheme S2: Synthesis of the diazotransfer reagent.

A suspension of NaN_3 (2g, 0.03 mol) in anhydrous MeCN (30 mL) is cooled to 0 °C. Sulfuryl chloride (2.5 mL, 0.03 mol) is then added drop-wise and the mixture stirred overnight at room temperature. Imidazole (3.98 g, 0.06 mmol) is added slowly to the reaction at 0 °C and the mixture is stirred for 3 h at room temperature. EtOAc (60 mL) is added and the reaction is washed with H_2O (2x 40 mL) and saturated aqueous $NaHCO_3$ (2x 40 mL). The organic fraction is then dried over $MgSO_4$ and filtered. In a separate flask, a solution of anhydrous HCl in EtOH is prepared at 0 °C by the dropwise addition of acetyl chloride (3.28 mL, 0.046 mol) to dry ethanol (7.5 mL).

The EtOAc solution is cooled to 0 °C and while stirring, the HCl solution in EtOH is added dropwise to precipitate a white solid. The precipitate is then filtered, washed with EtOAc (3x 15 mL), and dried under pressure to give imidazole-1-sulfonyl azide hydrochloride as white solid (5g, 76%). The NMR spectroscopic data were in agreement with those described in the literature.[3] **Caution!** Imidazole-1-sulfonyl azide and its salts (including chloride salt) are known to be energetic materials that are sensitive to chock and friction. Care should be taken while handling this material

and proper safety precautions should be used. Plastic container and materials are recommended for use.[4]

2- General procedure for the synthesis of azido acids 1a, 1b, and 1d

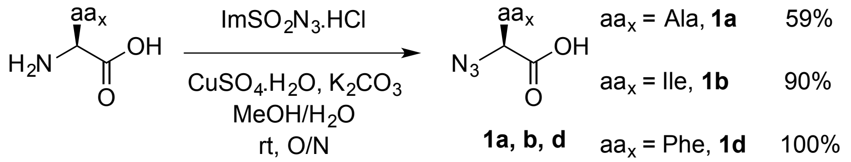

Scheme S3: Synthesis of azido acids **1**.

To a solution of the amino acid (1 eq.), K_2CO_3 (2.7 eq.), and $CuSO_4.5H_2O$ (0.01 eq.) in a MeOH/H2O mixture (5/1, v/v), $ImSO_2N_3$.HCl is added and the mixture stirred vigorously for 18h at room temperature. The reaction is then concentrated under reduced pressure, diluted with H_2O, acidified with 1M HCl to pH = 1, and extracted with 3x CH_2Cl_2. The organic layer is dried over $MgSO_4$, filtered, and concentrated. The crude obtained is then diluted in saturated aqueous $NaHCO_3$ and washed with Et_2O. The aqueous phase is acidified to pH with conc. HCl solution and extracted with 3x CH_2Cl_2. The combined organic layers are dried over $MgSO_4$, filtered, and concentrated. The corresponding azido acids are then used without any further purification.[3]

1a: *L*-Ala (1g, 11.2 mmol) in MeOH/H_2O (50 mL/10 mL) was treated according to the abovementioned procedure. Compound **1a** was obtained as pale yellow oil (755 mg, 59%). The NMR spectroscopic data were in agreement with those described in the literature.[5]

1b: *L*-Ile (1g, 7.6 mmol) in MeOH/H_2O (34 mL/7 mL) was treated according to the abovementioned procedure. Compound **1b** was obtained as colourless oil (1.03 g, 86%). The NMR spectroscopic data were in agreement with those described in the literature.[5]

1d: *L*-Phe (1g, 6.05 mmol) in MeOH/H_2O (27 mL/5.5 mL) was treated according to the abovementioned procedure. Compound **1d** was obtained as colourless oil (1.1 g, 100%). The NMR spectroscopic data were in agreement with those described in the literature.[5]

3- General procedure for the synthesis of amino alkynes 2a, 2b, and 2c[6]

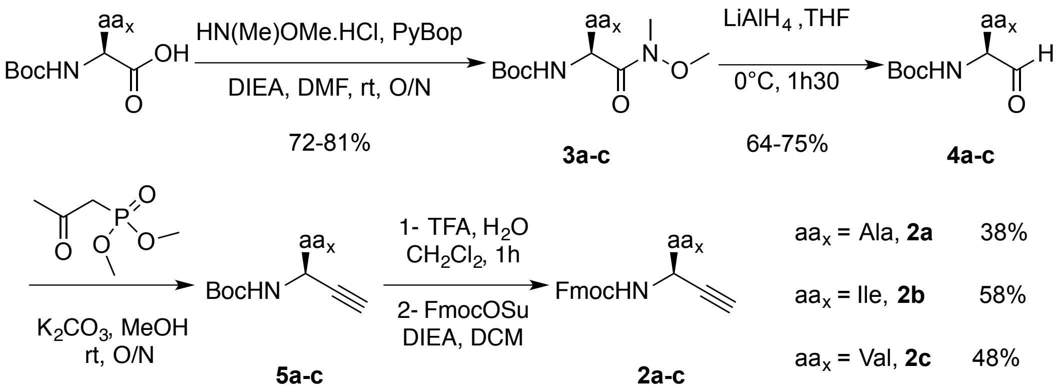

Scheme S4: Synthesis of Fmoc protected amino alkynes **2**.

3a-c: To a solution of BOC-protected amino acids (1 eq.) and DIEA (1.5 eq.) in DMF is added PyBop (1.1 eq.) and the reaction is stirred at rt for 0.5 h. Weinreb amine (1.1 eq.), DIEA (1.1 eq.) and DMF are then added to the reaction mixture and left at room temperature overnight. The reaction mixture is diluted with H_2O and extracted with 3x EtOAc. The combined organic fractions are washed successively with saturated aqueous $NaHCO_3$, $KHSO_4$ 1M, and brine. The organic fraction is dried over $MgSO_4$, filtered, and evaporated under reduced pressure. Purification over silica gel afforded the corresponding weinreb amide **3a-c**.

3a: Boc-*L*-Ala (1.5g, 7.9 mmol) in DMF (16 mL; 10 mL) was treated according to the abovementioned procedure. Purification over silica gel (30% EtOAc/70% CH_2Cl_2) afforded compound **3a** as white solid (1.3 g, 72%). The NMR spectroscopic data were in agreement with those described in the literature.[7]

3b: Boc-*L*-Ile (1g, 4.3 mmol) in DMF (8.5 mL; 5.5 mL) was treated according to the above mentioned procedure. Purification over silica gel (15% EtOAc/85% CH_2Cl_2) afforded compound **3b** as colourless oil (964 mg, 81%). The NMR spectroscopic data were in agreement with those described in the literature.[7]

3c: Boc-*L*-Val (1g, 4.6 mmol) in DMF (9 mL; 6 mL) was treated according to the abovementioned procedure. Purification over silica gel (20% EtOAc/80% CH_2Cl_2) afforded compound **3c** as colourless oil (919 mg, 77%). The NMR spectroscopic data

were in agreement with those described in the literature.[7]

4a-c: To a solution of the Weinreb amides **3a-c** (1 eq.) in anhydrous THF at 0 °C, $LiAlH_4$ (1.2 eq.) is added slowly and the reaction is stirred at 0 °C for 1.5 h. The reaction mixture is quenched by the addition of a solution of HCl 0.1M and extracted with 3x EtOAc. The combined organic fractions are washed with brine, dried over $MgSO_4$, filtered, and evaporated under reduced pressure. Purification over silica gel afforded the corresponding aldehydes **4a-c**.

4a: Weinreb amide **3a** (116 mg, 0.5 mmol) in THF (1.5 mL) was treated according to the abovementioned procedure. Purification over silica gel (10% EtOAc/90% CH_2Cl_2) afforded compound **4a** as white solid (69 mg, 75%). The NMR spectroscopic data were in agreement with those described in the literature.[7]

4b: Weinreb amide **3b** (116 mg, 0.5 mmol) in THF (1.5 mL) was treated according to the abovementioned procedure. Purification over silica gel (5% EtOAc/95% CH_2Cl_2) afforded compound **4b** as colourless oil (66 mg, 69%). The NMR spectroscopic data were in agreement with those described in the literature.[7]

4c: Weinreb amide **3c** (121 mg, 0.5 mmol) in THF (1.5 mL) was treated according to the abovementioned procedure. Purification over silica gel (5% EtOAc/95% CH_2Cl_2) afforded compound **4c** as colourless oil (60 mg, 64%). The NMR spectroscopic data were in agreement with those described in the literature.[7]

5a-c: To a solution of the aldehyde **4a-c** (1 eq.) and K_2CO_3 (2 eq.) in anhydrous MeOH, the Bestmann-Ohira reagent (dimethyl (1-diazo-2-oxopropyl)phosphonate; 1.2 eq.) is added and the reaction is stirred at rt overnight. The reaction mixture is diluted with Et_2O and washed successively with 5% aqueous $NaHCO_3$ and H_2O, dried over $MgSO_4$, filtered, and evaporated under reduced pressure. Purification over silica gel afforded the corresponding alkynes **5a-c**.

5a: Aldehyde **4a** (40 mg, 0.23 mmol) in MeOH (3.5 mL) was treated according to the abovementioned procedure. Purification over silica gel (CH_2Cl_2) afforded compound **5a** as white solid (24 mg, 61%). The NMR spectroscopic data were in agreement with those described in the literature.[8]

5b: Aldehyde **4b** (66 mg, 0.31 mmol) in MeOH (4.8 mL) was treated according to the abovementioned procedure. Purification over silica gel (CH_2Cl_2) afforded compound

5b as colourless oil. The NMR spectroscopic data were in agreement with those described in the literature.[8]

5c: Aldehyde **4c** (57 mg, 0.28 mmol) in MeOH (4.3 mL) was treated according to the abovementioned procedure. Purification over silica gel (10% pentane/90% CH_2Cl_2) afforded compound **5c** as white solid. The NMR spectroscopic data were in agreement with those described in the literature.[8]

6a-c: Alkyne **5a-c** (1 eq.) is diluted in CH_2Cl_2 (V) and TFA/H_2O (95:5; 2V) is added and stirred at rt until full deprotection of the compound. The solvent is evaporated and resuspended in toluene and evaporated 3 times until dryness. The crude is diluted in CH_2Cl_2 (V) and DIEA (1.1 eq.) and FmocOSu (2 eq.) are added and the reaction stirred at room temperature overnight. The mixture is washed successively with 3x HCl 1M, 3x $NaHCO_3$, and brine. The organic layer is dried over $MgSO_4$, filtered, and evaporated under reduced pressure. Purification over silica gel afforded the Fmoc protected alkyne **2a-c**.

2a: Boc-protected alkyne **5a** (345 mg, 2.04 mmol) in CH_2Cl_2 (11.9 mL) was treated according to the abovementioned procedure. Purification over silica gel (10% pentane/90% CH_2Cl_2) afforded compound **2a** as white solid (419 mg, 71%). The NMR spectroscopic data were in agreement with those described in the literature.[9]

2b: Boc-protected alkyne **5b** (1.27 g, 6 mmol) in CH_2Cl_2 (35 mL) was treated according to the abovementioned procedure. Purification over silica gel (10% pentane/90% CH_2Cl_2) afforded compound **2b** as white solid (1.64 g, 82%). The NMR spectroscopic data were in agreement with those described in the literature.[10]

2c: Boc-protected alkyne **5c** (527 mg, 2.74 mmol) in CH_2Cl_2 (16.1 mL) was treated according to the abovementioned procedure. Purification over silica gel (10% pentane/90% CH_2Cl_2) afforded compound **2c** as white solid (599 mg, 69%). The NMR spectroscopic data were in agreement with those described in the literature.[11]

IV. MS and HPLC data of starting and final cyclic peptides

Table S1: MS data, HRMS, retention time, and HPLC purities of starting peptides **P** (used for large scale synthesis of CP) and the MS data for the corresponding macrocyclic peptides **CP**

Ref	Peptide	MS $[M+H]^+$	HRMS*	rt	Purity**	MS of CP $[M+H]^+$
**P0**	VGAGIGFPAYD	1065.4	1065.5372	16.97	99%	699.4
**P1**	V^{Tz}GAGIGFPAYD	1089.4	1089.5473	17.32	99%	723.4
**P2**	VG^{Tz}AGIGFPAYD	1089.4	1089.5487	17.26	99%	723.4
**P3**	VGA^{Tz}GIGFPAYD	1089.4	1089.5473	17.38	99%	723.4
**P4**	$VGAG^{Tz}$IGFPAYD	1089.4	1089.5477	17.61	93%	723.4
**P5**	$VGAGI^{Tz}$GFPAYD	1089.4	1089.5468	17.7	97%	723.4
**P6**	$VGAGIG^{Tz}$FPAYD	1089.4	1111.5291	17.51	98%	723.4
**P13**	$V^{Tz}GA^{Tz}$GIGFPAYD	1113.4	1113.5600	17.64	96%	747.4
**P135**	$V^{Tz}GA^{Tz}GI^{Tz}$GFPAYD	1137.4	1137.5712	18.07	97%	771.4

* Calc. for **P0** $C_{50}H_{72}N_{12}O_{14}$ $[M+H]^+$: 1065.5364; Calc. for **P1-6** $C_{51}H_{72}N_{14}O_{13}$ $[M+H]^+$: 1089.5476; $[M+Na]^+$: 1111.5295; Calc. for **P13** $C_{52}H_{72}N_{16}O_{12}$ $[M+H]^+$: 1113.5588; Calc. for **P135** $C_{53}H_{72}N_{18}O_{11}$ $[M+H]^+$: 1137.5701.** Purity assessed by analytical HPLC at 220 nm UV absorption. LCMS traces of all precursor peptides **P** can be found in section IX.

Table S2: MS data, retention time, and HPLC purities of the remaining starting peptides **P** (used for small scale macrocyclisation reactions) and the MS data for the corresponding macrocyclic peptides.

Ref	Peptide	MS $[M+H]^+$	rt	Purity**	MS of CP $[M+H]^+$
**P14**	$V^{Tz}GAG^{Tz}$IGFPAYD	1113.4	17.86	98%	747.4
**P15**	$V^{Tz}GAGI^{Tz}$GFPAYD	1113.4	17.61	99%	747.4
**P16**	$V^{Tz}GAGIG^{Tz}$FPAYD	1113.4	17.59	99%	747.4
**P24**	$VG^{Tz}AG^{Tz}$IGFPAYD	1113.4	17.91	98%	747.4
**P25**	$VG^{Tz}AGI^{Tz}$GFPAYD	1113.4	17.69	98%	747.4
**P26**	$VG^{Tz}AGIG^{Tz}$FPAYD	1113.4	17.58	99%	747.4
**P35**	$VGA^{Tz}GI^{Tz}$GFPAYD	1113.4	17.96	98%	747.4
**P36**	$VGA^{Tz}GIG^{Tz}$FPAYD	1113.4	17.90	94%	747.4
**P46**	$VGAG^{Tz}IG^{Tz}$FPAYD	1113.4	18.00	97%	747.4
**P246**	$VG^{Tz}AG^{Tz}IG^{Tz}$FPAYD	1137.4	18.28	99%	771.4

.** Purity assessed by analytical HPLC at 220 nm UV absorption. LCMS traces of all precursor peptides **P** can be found in section IX.

Table S3: HRMS, HPLC data, and yields of the final cyclic peptides **CP**

	Cyclic Peptide	Ratio HPLC	Molecular formula	Expected $[M+H]^+$	Found $[M+H]^+$	Expected $[M+Na]^+$	Found $[M+Na]^+$	rt	Purity	Yield
**CP0**	VGAGIGFP	100%	$C_{34}H_{50}N_8O_8$	699.3824	**699.3816**	721.3644	**721.3629**	20.27	99%	32%
**CP1**	V^{Tz}GAGIGFP	100%	$C_{35}H_{50}N_{10}O_7$	723.3937	**-**	745.3756	**745.3754**	20.45	99%	43%
**CP2**	VG^{Tz}AGIGFP	100%	$C_{35}H_{50}N_{10}O_7$	723.3937	**723.3929**	745.3756	**745.3747**	21.08	91%	48%
**CP3**	VGA^{Tz}GIGFP	76%	$C_{35}H_{50}N_{10}O_7$	723.3937	**-**	745.3756	**745.3756**	18.37	99%	55%
		24%			**723.3942**		**745.3757**	20.63	97%	
**CP4**	$VGAG^{Tz}$IGFP	100%	$C_{35}H_{50}N_{10}O_7$	723.3937	**723.3927**	745.3756	**745.3744**	20.45	98%	34%
**CP5**	$VGAGI^{Tz}$GFP	76%	$C_{35}H_{50}N_{10}O_7$	723.3937	**723.3941**	745.3756	**745.3756**	20.54	93%	58%
**CP6**	$VGAGIG^{Tz}$FP	100%	$C_{35}H_{50}N_{10}O_7$	723.3937	**723.3927**	745.3756	**745.3746**	20.63	98%	40%
**CP13**	$V^{Tz}GA^{Tz}$GIGFP	20%	$C_{36}H_{50}N_{12}O_6$	747.4049	**747.4054**	769.3868	**769.3873**	20.11	98%	54%
		80%			**747.4046**		**769.3863**	20.19	98%	
**CP135**	$V^{Tz}GA^{Tz}GI^{Tz}$GFP	15%	$C_{37}H_{50}N_{14}O_5$	771.4161	**771.4162**	793.3981	**793.3980**	21.32	91%	39%
		85%			**771.4159**		**793.3979**	21.54	97%	

V. NMR data of final cyclic peptides

Copies of the proton NMR spectra for each compound depicting the different species by color code as well as copies of the HSQC spectra can be found in section V. Copies of the exchange spectroscopy (EXSY) spectra can be found in section VI.

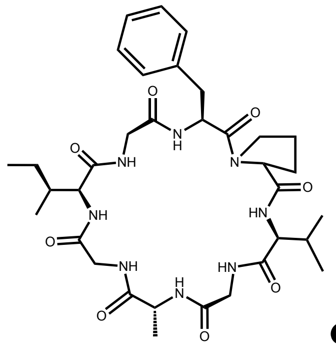

CP0; Cyclo(-VG$_a$AG$_b$IG$_c$FP-) **3:1** (conformers)

^{1}H NMR: (700 MHz, DMSO); HSQC and HMBC analysis (700 MHz, DMSO)*

Amino acid	Atom	^{1}H chemical shift	^{13}C chemical shift
Val	NH	8.10, d, *J* = 8.5	-
	α CH	3.76, t, *J* = 8.7	61.4
	β CH	2.02-1.96 m	29.3
	γ CH_3	0.82-0.78, m	15.5
			19.6
	CO		171.1
Gly (a)	NH	7.25-7.21, m	-
	α CH_2	3.92-3.86, m;<br>3.68-3.61, m	41.7
	CO	-	169.9
Ala	NH	8.56, d, *J* = 6.1	-
	α CH	4.12-4.07, m	49.3
	β CH_3	1.27, d, *J* = 7.2	17.5
	CO	-	173.3
Gly (b)	NH	8.47, t, *J* = 5.88	-
	α CH_2	3.97-3.92, m;<br>3.51-3.47, m	43.2
	CO	-	170.4
Ile	NH	7.85, d, *J* = 7.7	-
	α CH	4.02, d, *J* = 7.2	58.8
	β CH	1.81-1.75, m	36.2
	γ CH_2	1.42-1.35, m; 1.20-1.15, m	25.0
	γ CH_3	0.85-0.83, m	15.7
	δ CH_3	0.82-0.79, m	11.2
	CO	-	170.8
Gly (c)	NH	4.32, dd, *J* = 4.9, *J* = 6.8	-
	α CH_2	3.92-3.86, m;<br>3.68-3.61, m	41.7
	CO		170.7

Phe	NH	8.41, d, *J* = 3.5	-
	α CH	4.50, qt, *J* = 5.1	53.8
	β CH_2	3.07, dd, *J* = 5.7, *J* = 12.9; 2.86, dd, *J* = 10.4, *J* = 12.8	37.5
	Ar CH	7.33, t, *J* = 7.4	128.9
		7.28, t, *J* = 7.3	127.5
		7.25-7.21, m	129.9
	Ar C		136.4
	CO	-	170.4
Pro	α CH	3.61-3.58, m	60.7
	β CH_2	1.89-1.82, m; 0.87-0.84, m	30.2
	γ CH_2	1.63-1.56, m; 1.34-1.28, m	21.7
	δ CH_2	3.33-3.25, m; 3.23-3.18, m	46.2
	CO	-	170.4

*NMR data of the major conformer

^{1}H NMR: (700 MHz, DMSO); HSQC and HMBC analysis (700 MHz, DMSO)*

**Amino acid***	**Atom**	**^{1}H chemical shift**	**^{13}C chemical shift**
Val	NH	7.68, d, *J* = 7.5	-
	α CH	4.12-4.07, m	58.3
	β CH	2.09-2.03 m	30.7
	γ CH_3	0.85-0.83, m;	19.6
			18.2
	CO		171.5
Gly (a)	NH	8.24, t, *J* = 5.5	-
	α CH_2	3.82, t, *J* = 5.3; 3.58-3.53, m	43.0
	CO	-	169.2
Ala	NH	8.03, d, *J* = 7.5	-
	α CH	4.18, qt, *J* = 7.2	48.9
	β CH_3	1.22, d, *J* = 7.1	17.5
	CO	-	172.1
Gly (b)	NH	7.79, t, *J* = 5.6	-
	α CH_2	3.80, t, *J* = 5.7; 3.71-3.68, m	42.2
	CO	-	169.8
Ile	NH	8.18, d, *J* = 7.0	-
	α CH	3.97-3.95, m	58.1
	β CH	1.76-1.72, m	35.3
	γ CH_2	1.53-1.48, m; 1.12-1.05, m	24.9
	γ CH_3	0.83-0.80, m;	15.5
	δ CH_3	0.83-0.81, m	11.2
	CO	-	ND
Gly (c)	NH	8.63, t, *J* = 5.6	-
	α CH_2	3.97-3.92, m; 3.29-3.25, m	42.8
	CO		172.3
Phe	NH	7.61, d, *J* = 8.4	-
	α CH	4.71, dt, *J* = 4.4, *J* = 9.0	52.5
	β CH_2	2.95, dd, *J* = 4.2, *J* = 14.2; 2.78, dd, *J* = 9.6, *J* = 14.2	37.6

	Ar CH	7.25-7.21, 7.20-7.17 m	128.6, 129.4, 126.5
	Ar C	-	137.8
	CO	-	171.2
Pro	α CH	4.34, dd, $J = 5.0$, $J = 7.7$	61.2
	β CH_2	1.96-1.93, m; 1.25-1.23, m	28.0
	γ CH_2	1.89-1.82, m; 1.48-1.43, m	25.0
	δ CH_2	3.58-3.53, m	47.4
	CO	-	171.4

*NMR data of the minor conformer

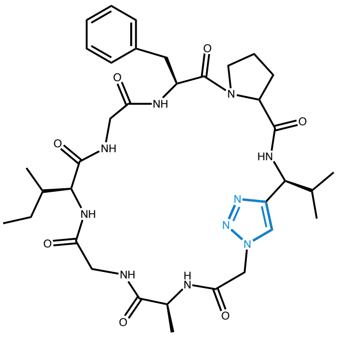

CP1; Cyclo(-VTzGaAG$_b$IG$_c$FP-) **4:1**

^{1}H NMR: (700 MHz, DMSO); HSQC and HMBC analysis (700 MHz, DMSO)*

**Amino acid***	**Atome**	**[1]H chemical shift**	**[13]C chemical shift**
Val	NH	8.51, d, $J = 9.3$	-
	α CH	4.63, t, $J = 9.6$	53.0
	β CH	2.02-1.98 m	33.0
	γ CH_3	0.65, d, $J = 6.7$;	20.3
		0.82, d, $J = 6.6$	19.8
	Tz CH	7.75, s	124.4
	Tz C		148.9
Gly (a)	α CH_2	5.16, s	51.6
	CO	-	167.2
Ala	NH	9.10, d, $J = 6.1$	-
	α CH	3.93, q, $J = 6.9$	50.2
	β CH_3	1.29, d, $J = 7.0$	16.6
	CO	-	171.9
Gly (b)	NH	8.39, dd, $J = 5.8$, $J = 6.4$	-
	α CH_2	4.01-3.98, m;	42.8
		3.40, dd, $J = 5.0$, $J = 16.3$	
	CO	-	169.3
Ile	NH	7.31	-
	α CH	4.01-3.98, m	57.7
	β CH	1.58-1.54, m	35.8
	γ CH_2	1.35-1.31, m; 1.02-0.98, m	25.2
	γ CH_3	0.88, d, $J = 6.8$;	15.9
	δ CH_3	0.69, t, $J = 7.4$	10.8
	CO	-	171.6
Gly (c)	NH	7.95-7.92, m	-
	α CH_2	3.83, dd, $J = 7.4$, $J = 16.7$;	41.9
		3.70, dd, $J = 4.2$, $J = 16.8$	

	CO		169.8
Phe	NH	8.49, d, *J* = 3.6	-
	α CH	4.22-4.19, m;	54.0
	β CH_2	2.98, dd, *J* = 6.1, *J* = 13.2;<br>2.83, dd, *J* = 10.0, *J* = 13.2	37.5
	Ar CH	7.32-7.18, m	129.9, 129.5, 129.0
	Ar C	-	136.4
	CO	-	ND
Pro	α CH	3.52-3.51, m	60.9
	β CH_2	2.02-1.97, m; 0.77-0.71, m	29.8
	γ CH_2	1.64-1.60, m; 1.32-1.27, m	21.9
	δ CH_2	3.38-3.31, m;<br>3.17, t, *J* = 10.0	46.3
	CO	-	169.3

*NMR data of the major conformer

^{1}H NMR: (700 MHz, DMSO); HSQC and HMBC analysis (700 MHz, DMSO)*

**Amino acid***	**Atome**	**^{1}H chemical shift**	**^{13}C chemical shift**
Val	NH	8.89, d, *J* = 7.6	-
	α CH	4.79, dd, *J* = 7.2, *J* = 8.8	51.6
	β CH	2.15-2.06 m	32.4
	γ CH_3	0.86-0.83, m;<br>0.81-0.77, m;	19.3<br>15.6
	Tz CH	7.65, s	123.5
	Tz C		147.7
Gly (a)	α CH_2	5.06, dd, *J* = 15.9, *J* = 52.9	51.9
	CO	-	165.3
Ala	NH	8.64, d, *J* = 7.7	-
	α CH	4.40, t, *J* = 7.0	48.8
	β CH_3	1.21, d, *J* = 6.8	18.4
	CO	-	172.1
Gly (b)	NH	8.56-8.55, m	-
	α CH_2	4.02-3.98, m; 3.86-3.85, m	41.7
	CO	-	ND
Ile	NH	7.44, d, *J* = 8.2	-
	α CH	2 t, *J* = 7.9	57.5
	β CH	1.66-1.64, m	35.9
	γ CH_2	1.48-1.43, m; 1.11-1.05, m	25.1
	CH_3	0.86-0.83, m;	14.5
	CH_3	0.81-0.77, m	11.0
	CO	-	ND
Gly (c)	NH	8.05-8.03, m	-
	α CH_2	3.55-3.53,m;<br>3.24, dd, *J* = 3.8, *J* = 16.3	42.4
	CO		ND
Phe	NH	7.93-7.92, m	-
	α CH	4.67-4.66, m;	52.4
	β CH_2	2.78-2.77, m; 2.99-2.97, m	36.5
	Ar CH	7.31-7.15, m	129.3, 128.5, 126.7

	Ar C	-	136.4
	CO	-	ND
Pro	α CH	4.32, dd, *J* = 4.0, *J* = 7.8	50.9
	β CH_2	2.03-2.02, m; 1.48-1.43, m	27.0
	γ CH_2	1.94-1.91, m	28.9
	δ CH_2	3.62-3.53, m	47.5
	CO	-	ND

*NMR data of the minor conformer

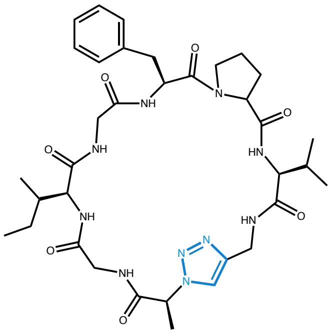

CP2; Cyclo(-VG$_a$TzAG$_b$IG$_c$FP-) **1:1**

^{1}H NMR: (700 MHz, DMSO); HSQC and HMBC analysis (700 MHz, DMSO)*

Amino acid*	Atome	[1]H chemical shift	[13]C chemical shift
Val	NH	7.75, d, *J* = 8.6	-
	α CH	3.80-3.76, m	61.6
	β CH	1.97-1.92 m	29.6
	γ CH_3	0.86-0.79, m	19.8/18.7
	CO		170.9
Gly (a)	NH	7.43, d, *J* = 6.7	-
	α CH_2	4.53, dd, *J* = 7.7, *J* = 14.6; 4.06-3.98, m	34.2
	Tz CH	8.12, s	122.3
	Tz C		144.1
Ala	α CH	5.63, q, *J* = 6.5	57.9
	β CH_3	1.67, d, *J* = 6.8	19.4
	CO	-	170.9
Gly (b)	NH	9.42-9.38, m	-
	α CH_2	4.08-4.06, m; 3.65, dd, *J* = 6.1, *J* = 14.2	43.7
	CO	-	171.9
Ile	NH	8.61, d, *J* = 5.9	-
	α CH	4.06-3.98, m	59.3
	β CH	1.92-1.88, m	35.8
	γ CH_2	1.78-1.74, m; 1.35-1.28, m	24.5
	γ CH_3	0.92, d, *J* = 6.8;	15.9
	δ CH_3	0.86-0.79, m	12.2
	CO	-	170.5
Gly (c)	NH	7.87-7.85, m	-
	α CH_2	3.76-3.71, m; 2.50	42.4
	CO		168.8
Phe	NH	7.98-7.97, m	-

	α CH	4.68-4.63, m;	52.6
	β CH_2	2.87-2.82, m; 2.70-2.65, m	36.6
	Ar CH	7.22-7.13, m	129.7, 128.5, 126.3
	Ar C		138.7
	CO	-	ND
Pro	α CH	3.43, d, J = 7.6	60.5
	β CH_2	1.78-1.74, m; 0.72-0.64, m	30.1
	γ CH_2	1.59-1.53, m; 1.35-1.28, m	21.7
	δ CH_2	3.30-3.25, m; 3.22-3.15, m	46.1
	CO	-	172.8

*NMR data of the first conformer

^{1}H NMR: (700 MHz, DMSO); HSQC and HMBC analysis (700 MHz, DMSO)*

**Amino acid***	**Atome**	**^{1}H chemical shift**	**^{13}C chemical shift**
Val	NH	7.66, d, J = 8.8	-
	α CH	4.06-3.98, m	59.3
	β CH	2.08-2.03, m	30.1
	γ CH_3	0.86-0.79, m	19.8/14.4
	CO		170.9
Gly (a)	NH	8.19-8.15, m	
	α CH_2	4.48, dd, J = 6.3, J = 15.5; 4.15, dd, J = 4.5, J = 15.0	34.9
	Tz CH	7.98, s	122.7
	Tz C		145.3
Ala	α CH	5.43, q, J = 6.8	58.4
	β CH_3	1.62, d, J = 6.8	18.9
	CO	-	169.0
Gly (b)	NH	8.41-8.45, m	-
	α CH_2	4.11, dd, J = 7.5, J = 16.9; 3.58, d, J = 15.6	41.7
	CO	-	169.3
Ile	NH	8.39, d, J = 7.1	-
	α CH	4.06-3.98, m	58.1
	β CH	1.74-1.69, m	36.1
	γ CH_2	1.50-1.42, m; 1.13-1.09, m	25.1
	γ CH_3	0.86-0.79, m	15.6
	δ CH_3	0.86-0.79, m	11.2
	CO	-	172.1
Gly (c)	NH	8.63-8.66, m	-
	α CH_2	4.06-3.98, m; 3.25-3.22, m	42.4
	CO	-	170.7
Phe	NH	7.38-7.35, m	-
	α CH	4.08-4.06, m	53.5
	β CH_2	2.93, dd, J = 3.8, J = 12.5; 2.87-2.82, m	37.9
	Ar CH	7.39-7.34, m; 7.33-7.29, m	130.0, 129.2, 127.6
	Ar C		136.6

	CO	-	170.5
Pro	α CH	4.31, d, $J = 8.4$	60.9
	β CH_2	1.88-1.82, m; 1.73-1.69, m	29.3
	γ CH_2	ND	ND
	δ CH_2	3.49-3.47, m; 2.50	47.2
	CO	-	171.6

*NMR data of the second conformer

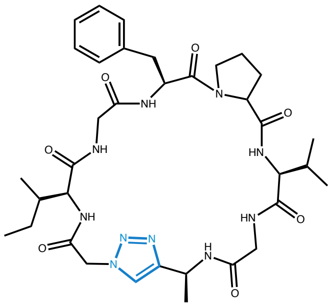

CP3; Cyclo(-VGaATzG$_b$IG$_c$FP-) **1:0.9**

^{1}H NMR: (700 MHz, DMSO); HSQC and HMBC analysis (700 MHz, DMSO)*

Amino acid*	Atome	^{1}H chemical shift	^{13}C chemical shift
Val	NH	7.89-7.83, m	-
	α CH	3.82-3.79, m	60.0
	β CH	1.95-1.92 m	29.1/29.6
	γ CH_3	0.96, d, $J = 6.6$;	19.6
		0.90-0.86, m	19.6
	CO		171.9
Gly (a)	NH	8.46-8.41, m	-
	α CH_2	4.09, dd, $J = 8.4$, $J = 16.8$;<br>3.44-3.38, m	42.4
	CO		168.6
Ala	NH	8.34, d, $J = 9.3$	-
	α CH	5.12-5.07, m	41.4
	β CH_3	1.37, d, $J = 6.9$	21.5
	Tz CH	7.69, s	123.2
	Tz C	-	150.3
Gly (b)	α CH_2	5.12-5.07, m	51.4
	CO	-	166.9
Ile	NH	8.79-8.76, m	-
	α CH	3.82-3.79, m	60.0
	β CH	1.77-1.73, m	35.2
	γ CH_2	1.65-1.58, m; 1.24-1.21, m	25.4
	γ CH_3	0.91-0.86, m	15.6
	δ CH_3	0.90-0.84, m	11.0
	CO	-	171.9
Gly (c)	NH	8.79-8.76, m	-
	α CH_2	3.92-3.87, m;<br>3.21, dd, $J = 4.5$, $J = 16.9$	42.4
	CO		168.4
Phe	NH	7.65, d, $J = 9.2$	-

	α CH	4.55-4.51, m	52.8
	β CH_2	2.70-2.66, m;<br>2.13-2.07, m	36.6
	Ar CH	7.22-7.13, m; 7.04-7.00, m	129.5, 128.3, 126.3
	Ar C		139.0
	CO	-	ND
Pro	α CH	4.30, t, *J* = 7.4	60.2
	β CH_2	2.19-2.13, m; 1.75-1.68, m	29.4
	γ CH_2	1.92-1.89, m; 1.87-1.83, m	25.6
	δ CH_2	3.66-3.59, m; 3.50-3.46, m	47.7
	CO	-	168.6

*NMR data of the major conformer

^{1}H NMR: (700 MHz, DMSO); HSQC and HMBC analysis (700 MHz, DMSO)*

**Amino acid***	**Atome**	**^{1}H chemical shift**	**^{13}C chemical shift**
Val	NH	8.65-8.61, m	-
	α CH	3.70, dd, *J* = 6.3, *J* = 9.0	61.6
	β CH	1.95-1.92 m	29.1/29.6
	γ CH_3	0.94, d, *J* = 6.6;<br>0.87-0.85, m	20.0<br>19.5
	CO		172.3
Gly (a)	NH	8.13-8.07, m	-
	α CH_2	3.77, dd, *J* = 6.0, *J* = 17.3;<br>3.66-3.59, m	42.0
	CO		168.7
Ala	NH	7.96, d, *J* = 8.4	-
	α CH	5.25-5.17, m	42.6
	β CH_3	1.26, d, *J* = 7.0	23.3
	Tz CH	8.01, s	122.3
	Tz C	-	150.7
Gly (b)	α CH_2	5.25-5.17, m;<br>5.15-5.12, m	51.9
	CO	-	166.7
Ile	NH	8.79-8.76, m	-
	α CH	3.94-3.90, m	59.3
	β CH	1.81-1.77, m	34.9
	γ CH_2	1.57-1.52, m; 1.21-1.17, m	25.0
	s	0.89-0.83, m	15.6
	δ CH_3	0.90-0.84, m	11.0
	CO	-	171.6
Gly (c)	NH	8.65-8.61, m	-
	α CH_2	3.87-3.83, m;<br>3.54, dd, *J* = 5.8, *J* = 16.9	43.1
	CO		169.8
Phe	NH	7.45, d, *J* = 5.8	-
	α CH	4.55-4.51, m	52.8
	β CH_2	2.75, dd, *J* = 8.3, *J* = 12.8;<br>2.69-2.62, m	38.2
	Ar CH	7.30-7.20, m; 7.10-7.06, m	130.0, 128.8, 127.2

	Ar C		136.40
	CO	-	169.8
Pro	α CH	3.97-3.94, m	59.5
	β CH_2	1.80-1.76, m; 1.53-1.48, m	31.0
	γ CH_2	1.65-1.58, m; 1.51-1.46, m	22.2
	δ CH_2	3.29-3.25, m; 3.17-3.12, m	46.8
	CO	-	172.6

*NMR data of the minor conformer

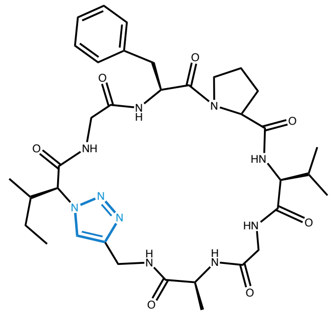

CP4; Cyclo(-VGaAG$_b$TzIG$_c$FP-) **1:1**

^{1}H NMR: (700 MHz, DMSO); HSQC and HMBC analysis (700 MHz, DMSO)*

**Amino acid***	**Atome**	**^{1}H chemical shift**	**^{13}C chemical shift**
Val	NH	7.88-7.85, m	-
	α CH	4.08, dd, J = 6.4, J = 8.3	58.4
	β CH	2.04-1.95, m	30.3
	γ CH_3	0.82, d, J = 6.7;	19.8
		0.81, d, J = 6.7	18.4
	CO		171.4
Gly (a)	NH	8.16-8.14, m	-
	α CH_2	3.93, dd, J = 6.4, J = 16.2;	42.6
		3.40-3.38, m	
	CO		168.4
Ala	NH	7.98-7.96, m	-
	α CH	4.32, quintet, J = 7.2	48.4
	β CH_3	1.20, d, J = 7.1	19.1
	CO	-	172.4
Gly (b)	NH	8.53, dd, J = 5.0, J = 7.0	-
	α CH_2	4.59-4.58, m; 4.05-4.00, m	34.4
	Tz CH	8.02, s	121.4
	Tz C	-	145.3
Ile	α CH	5.19, d, J = 10.1	66.8
	β CH	2.16-2.09, m	37.7
	γ CH_2	1.01-0.95, m; 0.86-0.83, m	24.7
	γ CH_3	0.87, d, J = 6.7	15.4
	δ CH_3	0.78-0.73, m	10.5
	CO	-	168.1
Gly (c)	NH	8.99, dd, J = 3.5, J = 7.9	-
	α CH_2	4.22, dd, J = 7.9, J = 15.5;	42.6
		3.25-3.22, m	
	CO		168.8

Phe	NH	8.44, d, J = 7.8	-
	α CH	4.50-4.46, m	53.7
	β CH_2	2.99, dd, J = 4.1, J = 14.0; 2.78-2.74, m	37.0
	Ar CH	7.31-7.29, m; 7.28-7.24, m; 7.22-7.19, m	129.7, 128.6, 127.0
	Ar C		137.6
	CO	-	170.0
Pro	α CH	4.46-4.44, m	60.5
	β CH_2	2.04-1.95, m; 1.80-1.72, m	27.9
	γ CH_2	1.80-1.72, m	25.1
	δ CH_2	3.53-3.50, m; 3.43-3.40, m	47.2
	CO	-	171.2

*NMR data of the first conformer

^{1}H NMR: (700 MHz, DMSO); HSQC and HMBC analysis (700 MHz, DMSO)*

Amino acid*	Atome	^{1}H chemical shift	^{13}C chemical shift
Val	NH	8.01, d, J = 6.5	-
	α CH	3.87, t, J = 6.5	59.6
	β CH	1.92-1.85, m	30.3
	γ CH_3	0.78-0.73, m; 0.72, d, J = 6.8	19.4, 18.9
	CO		171.4
Gly (a)	NH	8.00-7.98, m	-
	α CH_2	3.67, ddd, J = 5.4, J = 16.4, J = 50.3	42.8
	CO		169.3
Ala	NH	7.87-7.85, m	-
	α CH	4.17-4.13, m	48.9
	β CH_3	1.20, d, J = 7.1	17.7
	CO	-	172.1
Gly (b)	NH	8.20, dd, J = 5.4, J = 6.7	-
	α CH_2	4.57-4.53, m; 4.01-3.99, m	34.5
	Tz CH	7.85, s	121.8
	Tz C	-	145.6
Ile	α CH	5.07, d, J = 10.1	67.4
	β CH	2.16-2.09, m	37.7
	γ CH_2	1.01-0.95, m; 0.86-0.83, m	24.7
	γ CH_3	0.87, d, J = 6.7	15.4
	δ CH_3	0.78-0.73, m	10.5
	CO	-	167.7
Gly (c)	NH	8.62, dd, J = 4.1, J = 7.2	-
	α CH_2	4.05-4.03, m; 3.40-3.37, m	41.9
	CO		167.9
Phe	NH	8.15-8.12, m	-
	α CH	4.57-4.53, m	52.3
	β CH_2	2.85, dd, J = 7.3, J = 13.3; 2.80-2.77, m	39.1

	Ar CH	7.28-7.24, m; 7.22-7.19, m	129.7, 128.3, 126.3
		7.19-7.18, m	137.6
	Ar C		
	CO	-	169.8
Pro	α CH	4.17-4.13, m	59.5
	β CH_2	1.92-1.85, m; 1.66-1.57, m	31.6
	γ CH_2	1.66-1.57, m	22.1
	δ CH_2	3.36-3.32, m; 3.27-3.25, m	46.8
	CO	-	171.9

*NMR data of the second conformer

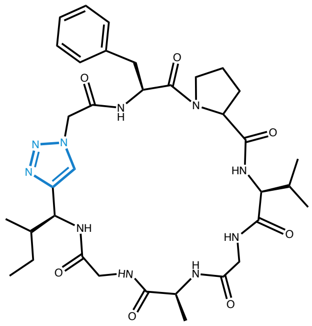

CP5; Cyclo(-VGaAG$_b$ITzG$_c$FP-) **1:0.6**

^{1}H NMR: (700 MHz, DMSO); HSQC and HMBC analysis (700 MHz, DMSO)*

Amino acid*	Atome	^{1}H chemical shift	^{13}C chemical shift
Val	NH	7.86, d, J = 8.8	-
	α CH	4.13, dd, J = 5.5, J = 8.7	58.1
	β CH	2.15-2.09, m	29.7
	γ CH_3	0.67, t, J = 7.1;	19.5
		0.44, d, J = 6.9	17.3
	CO		172.4
Gly (a)	NH	8.05, t, J = 4.7	-
	α CH_2	3.79-3.74, m;	44.4
		3.64, dd, J = 5.2, J = 14.8	
	CO		171.5
Ala	NH	8.76, d, J = 5.0	-
	α CH	4.06-4.00, m	50.6
	β CH_3	1.29, d, J = 7.4	16.7
	CO	-	172.9
Gly (b)	NH	8.43, t, J = 6.3	-
	α CH_2	3.82, dd, J = 6.8, J = 16.9;	43.1
		3.44-3.40, m	
	CO	-	168.3
Ile	NH	7.30-7.27, m	-
	α CH	5.15-5.09, m	49.2
	β CH	1.67-1.64, m	40.4
	γ CH_2	1.35-2.30, m; 1.00-0.96, m	25.6
	γ CH_3	0.77, d, J = 6.8	14.5
	δ CH_3	0.84, t, J = 7.4	11.8
	Tz CH	8.18, s	124.5
	Tz C	-	147.8

Gly (c)	α CH_2	5.15-5.09, m	51.3
	CO		166.2
Phe	NH	9.04-9.00, m	-
	α CH	4.70, q, J = 7.4	52.7
	β CH_2	2.96-2.90, m	38.0
	Ar CH	7.36-7.21, m	128.9, 127.4, 129.8
	Ar C		136.4
	CO	-	170.7
Pro	α CH	3.90-3.86, m	60.5
	β CH_2	1.91-1.84, m; 1.68-1.58, m	30.2
	γ CH_2	1.63-1.58, m; 1.41-1.36, m	21.9
	δ CH_2	3.34-3.22, m	46.1
	CO	-	171.2

*NMR data of the major species

^{1}H NMR: (700 MHz, DMSO); HSQC and HMBC analysis (700 MHz, DMSO)*

**Amino acid***	**Atome**	**^{1}H chemical shift**	**^{13}C chemical shift**
Val	NH	7.94, d, J = 7.7	-
	α CH	3.79-3.74, m	60.1
	β CH	1.84-1.78, m	29.5
	γ CH_3	0.75, d, J = 6.8;	19.8
		0.67, t, J = 7.1;	19.5
	CO		171.5
Gly (a)	NH	7.58, t, J = 5.7	-
	α CH_2	3.90-3.86, m;	42.6
		3.59, dd, J = 5.0, J = 16.2	
	CO		170.3
Ala	NH	8.50, d, J = 6.0	-
	α CH	3.95-3.90, m	49.8
	β CH_3	1.25, d, J = 7.0	16.9
	CO	-	172.4
Gly (b)	NH	8.36, dd, J = 5.7, J = 6.7	-
	α CH_2	3.98-3.92, m; 3.38-3.33, m	43.0
	CO	-	168.5
Ile	NH	7.63, d, J = 9.6	-
	α CH	4.88, dd, J = 7.7, J = 9.3	50.1
	β CH	1.74-1.70, m	39.3
	γ CH_2	1.44-1.41, m; 1.07-1.02, m	24.9
	γ CH_3	0.71, d, J = 6.7	15.7
	δ CH_3	0.80, t, J = 7.5	11.6
	Tz CH	7.98, s	124.1
	Tz C	-	148.6
Gly (c)	α CH_2	5.28, d; 5.02, d, J = 16.8	51.3
	CO		165.6
Phe	NH	9.04-9.00, m	-
	α CH	4.54, q, J = 7.2	53.3
	β CH_2	2.96-2.90, m	38.4
	Ar CH	7.36-7.21, m	128.9, 127.4, 129.8

	Ar C		136.4
	CO	-	170.1
Pro	α CH	3.90-3.86, m	61.0
	β CH_2	1.91-1.84, m; 1.68-1.58, m	30.4
	γ CH_2	1.63-1.58, m; 1.51-1.42, m	24.9
	δ CH_2	3.34-3.22, m	46.1
	CO	-	170.9

*NMR data of the minor species

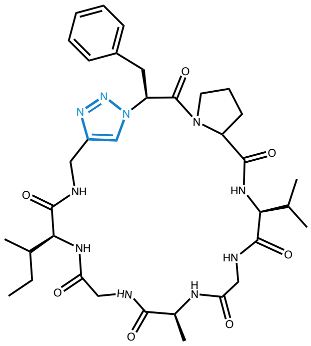

CP6; Cyclo(-VGaAG$_b$IG$_c$TzFP-) **2:1**

^{1}H NMR: (700 MHz, DMSO); HSQC and HMBC analysis (700 MHz, DMSO)*

Amino acid*	Atome	[1]H chemical shift	[13]C chemical shift
Val	NH	7.80-7.78, m	-
	α CH	4.20-4.17, m	58.1
	β CH	2.06-2.00, m	31.4
	γ CH_3	0.88-0.85, m	19.8
		0.87-0.84, m	18.6
	CO		171.3
Gly (a)	NH	8.51, t, J = 5.7	-
	α CH_2	3.93, d, J = 15.5;<br>3.50-3.49, m	43.3
	CO		168.9
Ala	NH	8.07, d, J = 7.8	-
	α CH	4.32-4.25, m	48.6
	β CH_3	1.24, d, J = 7.1	18.4
	CO	-	172.5
Gly (b)	NH	8.12-8.10, m	-
	α CH_2	3.87, dd, J = 6.7, J = 16.6;<br>3.63, dd, J = 4.8, J = 16.7	42.6
	CO	-	168.7
Ile	NH	7.89, d, J = 8.5	-
	α CH	4.11-4.07, m	57.5
	β CH	1.80-1.76, m	36.3
	γ CH_2	1.50-1.43, m; 1.08-1.03, m	24.9
	γ CH_3	0.87-0.83, m	15.8
	δ CH_3	0.84-0.80, m	11.4
	CO	-	171.3
Gly (c)	NH	8.58, dd, J = 4.6, J = 7.3	-
	α CH_2	4.59, dd, J = 7.7, J = 15.3;	34.7

		3.93, dd, $J = 3.2$, $J = 15.4$	
	Tz CH	8.10, s	123.0
	Tz C	-	145.4
Phe	α CH	5.85, dd, $J = 3.8$, $J = 11.6$	62.8
	β CH_2	3.47-3.45, m;	38.0
		3.18, dd, $J = 11.8$, $J = 14.5$	
	Ar CH	7.24-7.21, m;	127.4
		7.20-7.18, m;	128.8
		7.11, d, $J = 7.1$	129.3
	Ar C		135.8
	CO	-	167.8
Pro	α CH	4.45-4.42, m	61.0
	β CH_2	2.06-2.00, m; 1.97-1.91, m	27.9
	γ CH_2	1.97-1.88, m	25.2
	δ CH_2	3.84-3.78, m	47.7
	CO	-	170.4

*NMR data of the major conformer

^{1}H NMR: (700 MHz, DMSO); HSQC and HMBC analysis (700 MHz, DMSO)*

**Amino acid***	**Atome**	**^{1}H chemical shift**	**^{13}C chemical shift**
Val	NH	8.33, d, $J = 5.1$	-
	α CH	3.73, dd, $J = 5.3$, $J = 7.5$	61.0
	β CH	1.94-1.89, m	29.3
	γ CH_3	0.94, d, $J = 6.7$;	19.8
		0.88-0.85, m	19.8
	CO		171.3
Gly (a)	NH	8.27-8.22, m	-
	α CH_2	3.78-3.75, m; 3.53-3.50, m	42.6
	CO		168.7
Ala	NH	7.82, d, $J = 7.6$	-
	α CH	4.32-4.25, m	48.6
	β CH_3	1.11, d, $J = 7.2$	18.0
	CO	-	173.2
Gly (b)	NH	8.31, t, $J = 5.4$	-
	α CH_2	3.88, dd, $J = 5.6$, $J = 15.7$;	43.3
		3.45-3.42, m	
	CO	-	169.4
Ile	NH	7.74, d, $J = 8.4$	-
	α CH	4.12-4.08, m	57.5
	β CH	1.89-1.82, m	36.5
	γ CH_2	1.36-1.31, m; 1.11-1.08, m	24.5
	γ CH_3	0.83-0.80, m	15.8
	δ CH_3	0.81-0.78, m	11.4
	CO	-	170.8
Gly (c)	NH	8.27-8.22, m	-
	α CH_2	4.48-4.45, m; 4.17-4.13, m	34.7
	Tz CH	7.79, s	123.0
	Tz C	-	144.7

Phe	α CH	5.26, t, *J* = 7.7	62.4
	β CH_2	3.49-3.47, m;	38.2
		3.35, dd, *J* = 7.9, *J* = 13.4	
	Ar CH	7.29-7.26, m;	128.8
		7.27-7.24, m	127.4
		7.18-7.16, m	129.7
	Ar C		135.8
	CO	-	166.4
Pro	α CH	4.17-4.13, m	59.6
	β CH_2	1.95-1.91, m; 1.87-1.83, m	32.1
	γ CH_2	1.63-1.55, m; 1.28-1.24, m	22.4
	δ CH_2	3.34-3.30, m; 3.26-3.22, m	47.3
	CO	-	171.8

*NMR data of the minor conformer

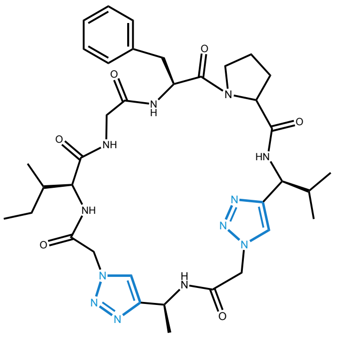

CP13; Cyclo(-VTz$_a$G$_a$ATz$_b$G$_b$IG$_c$FP-) **3:1**

^{1}H NMR: (700 MHz, DMSO); HSQC and HMBC analysis (700 MHz, DMSO)*

**Amino acid***	**Atome**	**^{1}H chemical shift**	**^{13}C chemical shift**
Val	NH	7.68, d, *J* = 8.6	-
	α CH	4.83, t, *J* = 7.5	51.4
	β CH	2.13-2.07, m	32.6
	γ CH_3	0.85-0.83, m	19.3
		0.83-0.80, m	18.9
	Tz CH	7.81, s	123.9
	Tz C	-	147.4
Gly (a)	α CH_2	5.16, d, *J* = 16.0	52.1
		5.01, d, *J* = 16.4	
	CO		164.9
Ala	NH	8.89, d, *J* = 7.7	-
	α CH	5.04, t, *J* = 7.4	41.7
	β CH_3	1.44, d, *J* = 6.9	21.0
	Tz CH	7.71, s	123.2
	Tz C	-	149.6
Gly (b)	α CH_2	4.71, d, *J* = 15.9;	51.2
		5.09, d, *J* = 16.4	
	CO	-	165.9
Ile	NH	8.65, d, *J* = 7.0	-
	α CH	4.08, t, *J* = 7.6	58.2
	β CH	1.72-1.66, m	36.3
	γ CH_2	1.49-1.45, m; 1.17-1.10, m	29.3

	γ CH_3	0.85-0.81, m	15.6
	δ CH_3	0.85-0.81, m	11.4
	CO	-	171.6
Gly (c)	NH	8.60-8.56, m	-
	α CH_2	4.07-4.02, m;<br>3.30, dd, *J* = 2.2, *J* = 16.5	42.4
	CO	-	168.9
Phe	NH	7. 09, d, *J* = 7.7	-
	α CH	4.67-4.62, m	52.4
	β CH_2	2.99, dd, *J* = 3.4, *J* = 14.2;<br>2.78, dd, *J* = 10.6, *J* = 14.2	36.5
	Ar CH	7.27-7.24, m;<br>7.21-7.18, m;<br>7.11-7.10, m	128.8<br>126.7<br>129.8
	Ar C		138.5
	CO	-	ND
Pro	α CH	4.31, d, *J* = 8.5	61.0
	β CH_2	2.06-1.97, m; 1.90-1.85, m	27.1
	γ CH_2	1.90-1.85, m; 1.84-1.80, m	24.9
	δ CH_2	3.60-3.56, m	47.4
	CO	-	170.6

*NMR data of the major conformer

^{1}H NMR: (700 MHz, DMSO); HSQC and HMBC analysis (700 MHz, DMSO)*

**Amino acid***	**Atome**	**^{1}H chemical shift**	**^{13}C chemical shift**
Val	NH	8.63, d, *J* = 9.4	-
	α CH	4.55, d, *J* = 9.3	
	β CH	2.13-2.07, m	
	γ CH_3	0.92, d, *J* = 6.5<br>0.74, d, *J* = 6.5	15.8<br>20.0
	Tz CH	7.52, s	
	Tz C	-	
Gly (a)	α CH_2	5.21, d, *J* = 16.5;<br>4.99-4.95, m	51.9
	CO		
Ala	NH	8.89-8.87	-
	α CH	5.06-5.03	
	β CH_3	1.51, d, *J* = 7.1	20.7
	Tz CH	7.81, s	
	Tz C	-	
Gly (b)	α CH_2	5.14-5.10, m	52.4
	CO	-	
Ile	NH	8.56, d, *J* = 4.5	-
	α CH	4.06-4.04	59.3
	β CH	1.80-1.76, m	36.1
	γ CH_2	1.50-1.43, m	
	γ CH_3	0.87-0.84, m	14.3
	δ CH_3	0.89-0.87, m	11.5

	CO	-	
Gly (c)	NH	8.52, t, *J* = 5.7	-
	α CH_2	3.82-3.78, m; 3.69-3.65, m	
	CO	-	
Phe	NH	7.69-7.67	
	α CH	4.13-4.09, m	
	β CH_2	2.66-2.61, m;<br>ND	
	Ar CH		
	Ar C		
	CO	-	
Pro	α CH	3.26-3.22, m	
	β CH_2	1.59-1.54, m	
	γ CH_2	1.31-1.27, m	
	δ CH_2	3.20-3.17, m; 3.14-3.13, m	
	CO	-	

*NMR data of the minor conformer

VI. NMR spectra of final cyclic peptides

CP0; Cyclo(-VG$_a$AG$_b$IG$_c$FP-); ^{1}H; DMSO-d6; 700 MHz; 3:1 ratio

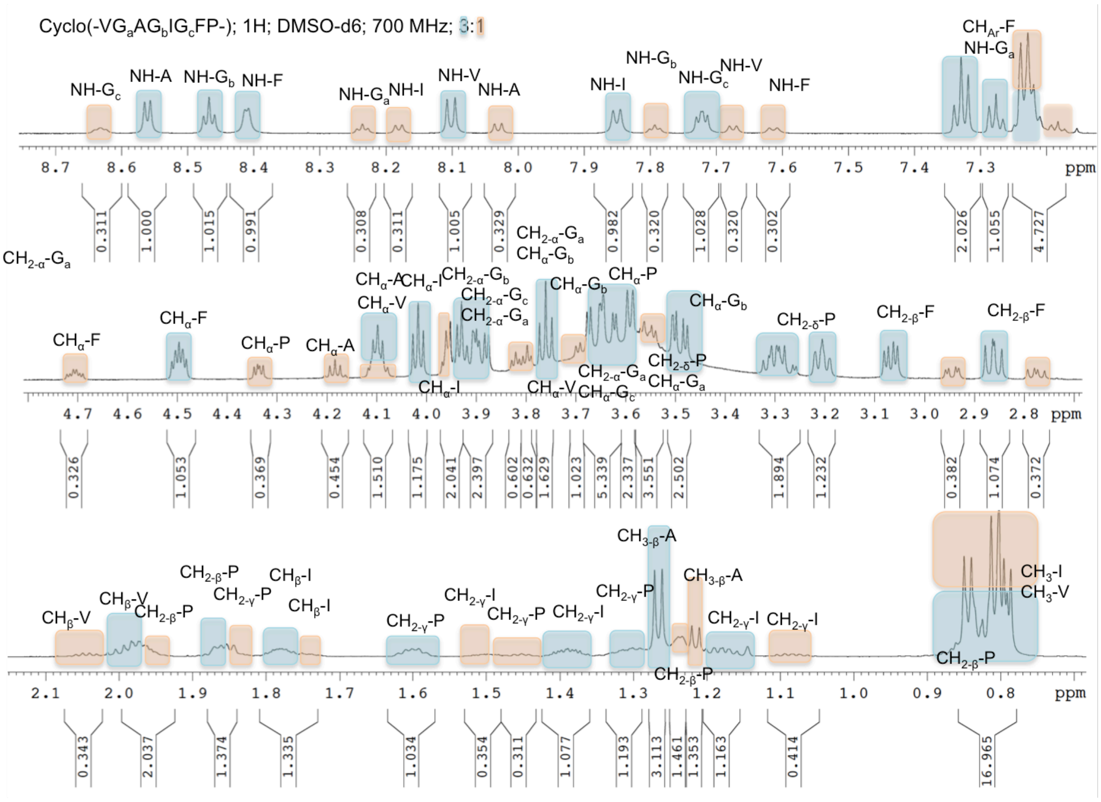

CP0; Cyclo(-VG$_a$AG$_b$IG$_c$FP-); HSQC; DMSO-d6; 700 MHz

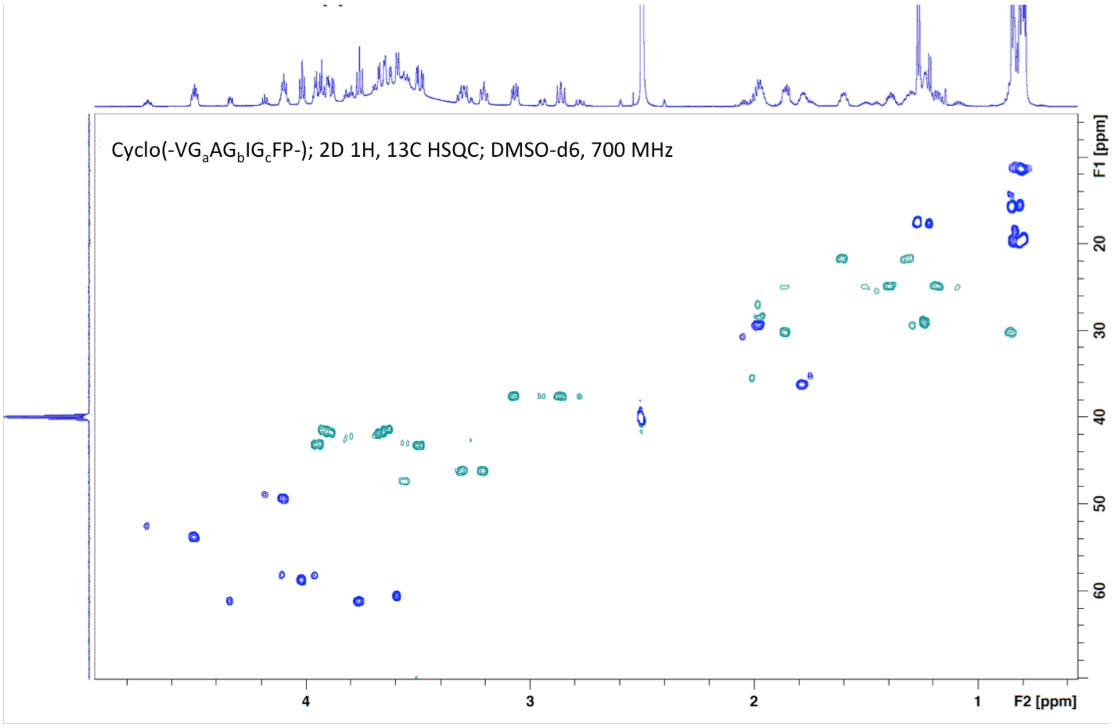

CP1; Cyclo(-VTzG$_a$AG$_b$IG$_c$FP-); ^{1}H; DMSO-d6; 700 MHz; 4:1 ratio

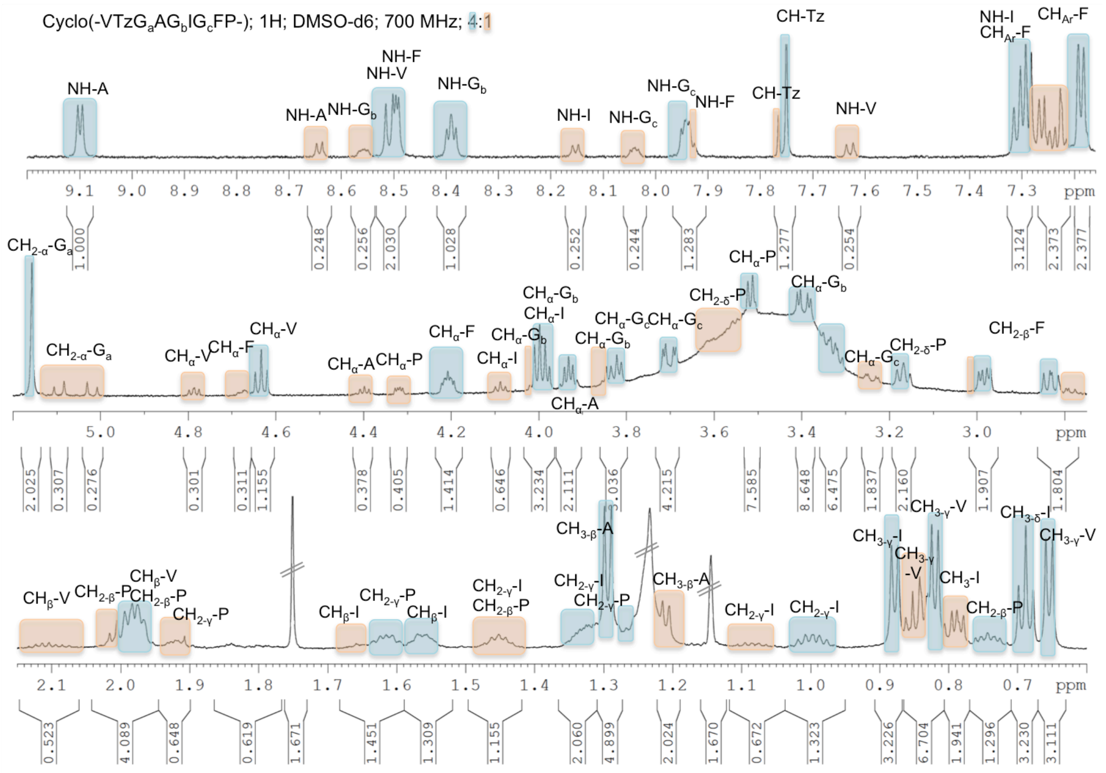

CP1; Cyclo(-VTzG$_a$AG$_b$IG$_c$FP-); HSQC; DMSO-d6; 700 MHz

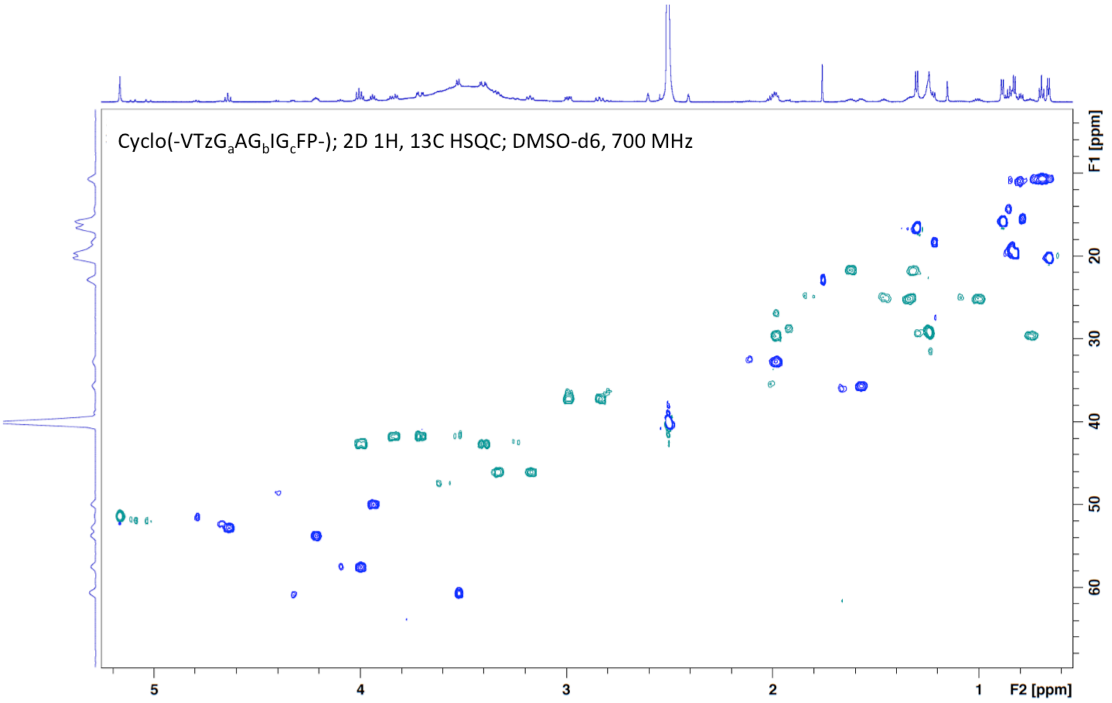

CP2; Cyclo(-VG$_a$TzAG$_b$IG$_c$FP-); ^{1}H; DMSO-d6; 700 MHz; 1:1 ratio

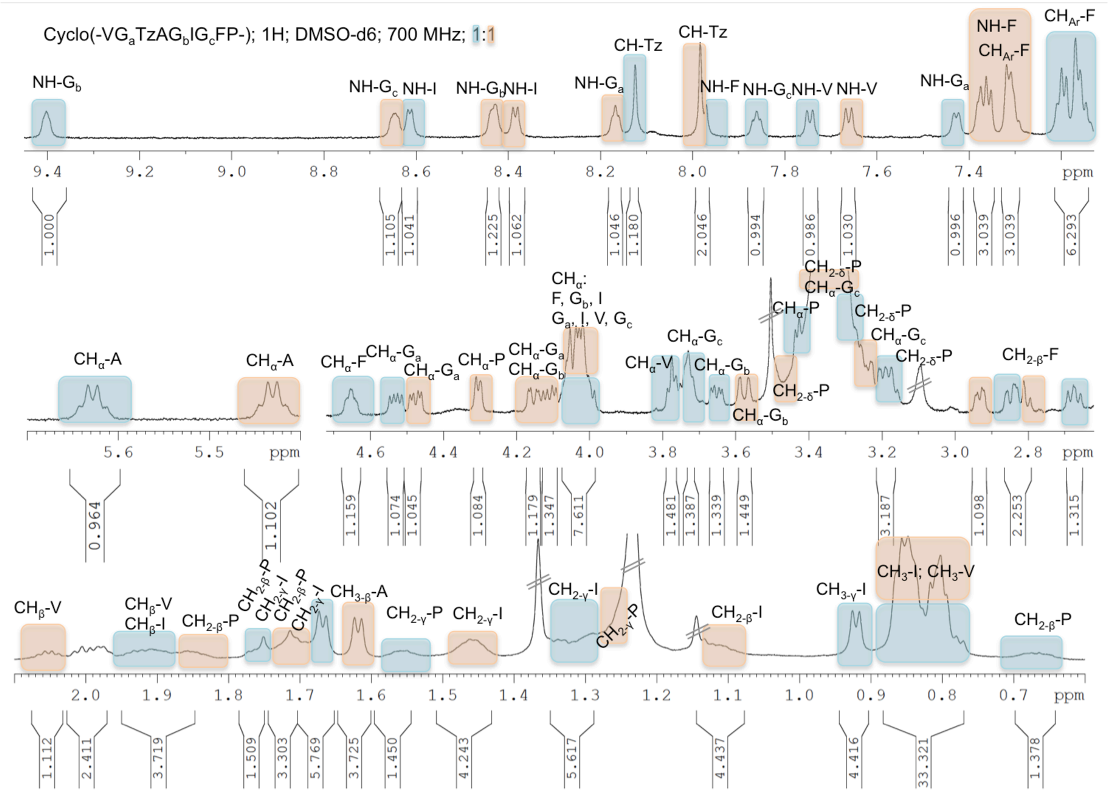

CP2; Cyclo(-VG$_a$TzAG$_b$IG$_c$FP-); HSQC; DMSO-d6; 700 MHz

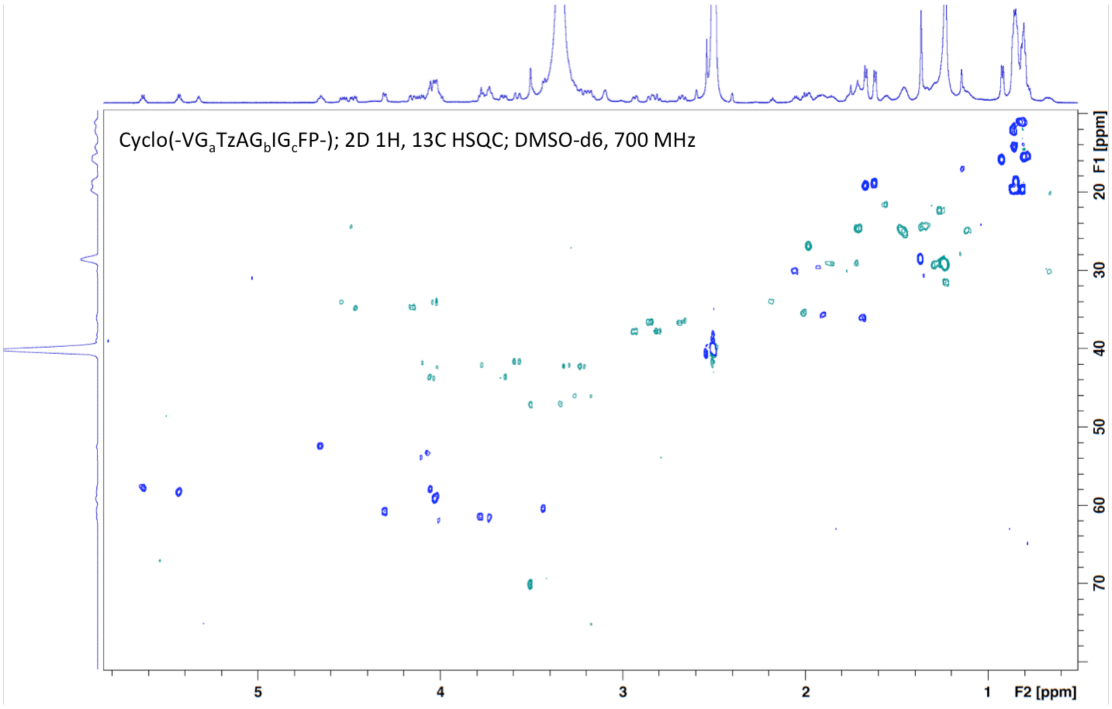

CP3; Cyclo(-VG$_a$ATzG$_b$IG$_c$FP-); ^{1}H; DMSO-d6; 700 MHz; 1:0.9 ratio

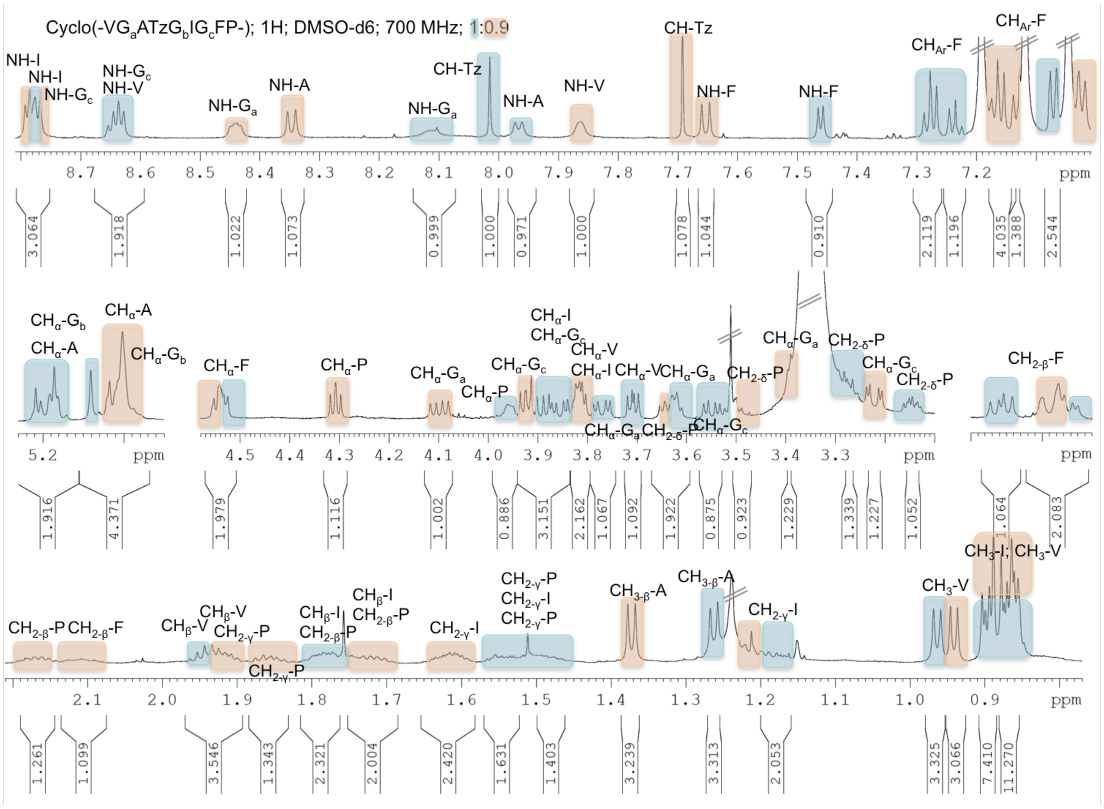

CP3; Cyclo(-VG$_a$ATzG$_b$IG$_c$FP-); HSQC; DMSO-d6; 700 MHz

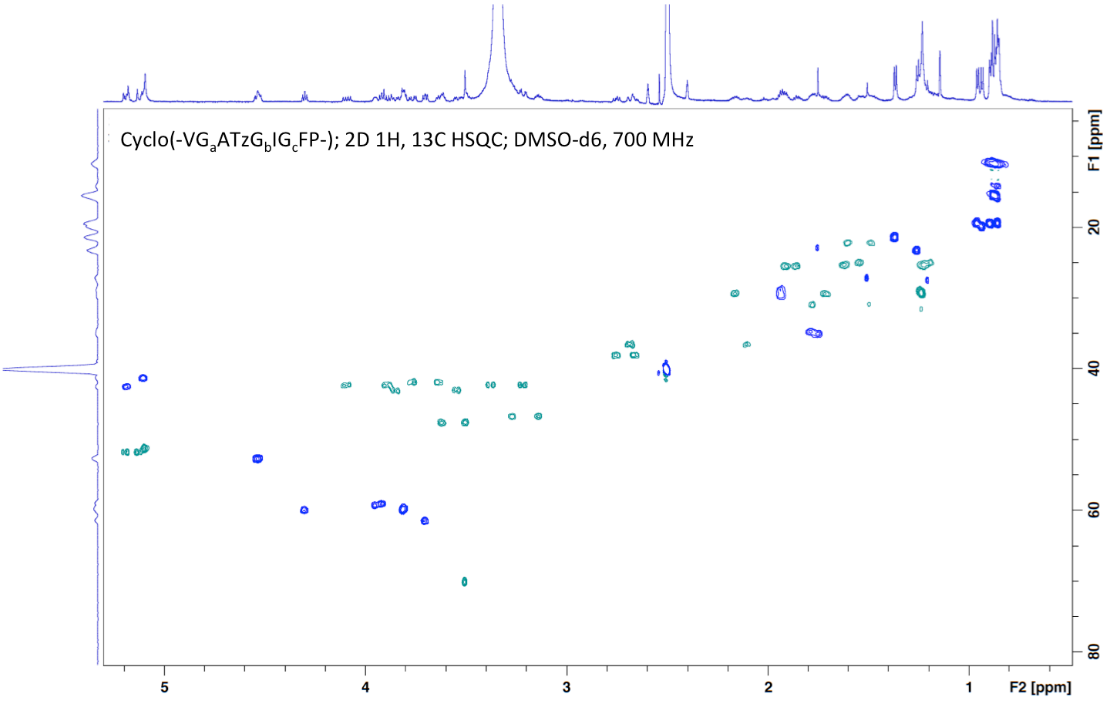

CP4; Cyclo(-VG$_a$AG$_b$TzIG$_c$FP-); ^{1}H; DMSO-d6; 700 MHz; 1:1 ratio

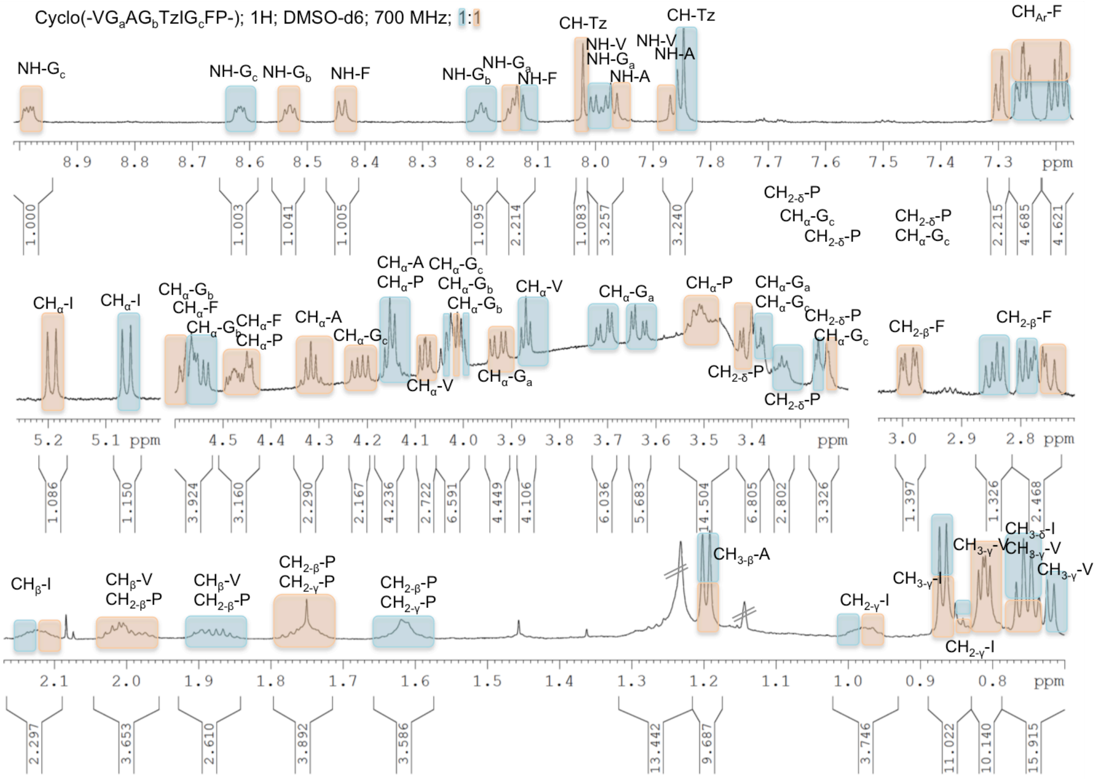

CP4; Cyclo(-VG$_a$AG$_b$TzIG$_c$FP-); HSQC; DMSO-d6; 700 MHz

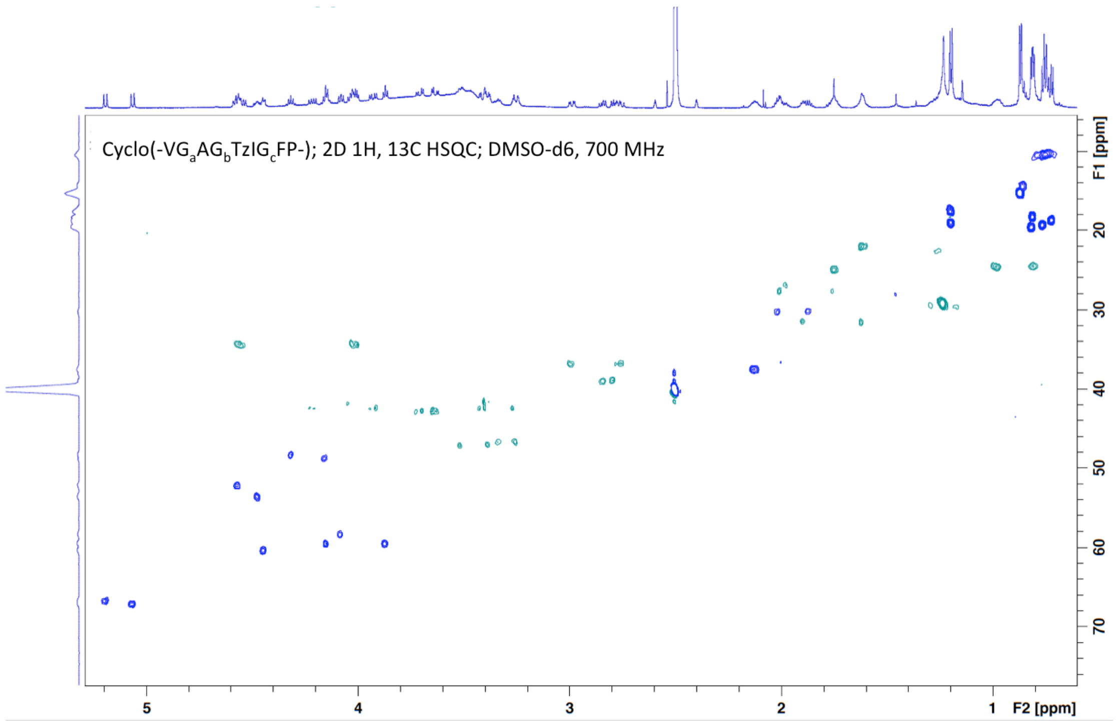

CP5; Cyclo(-VG$_a$AG$_b$ITzG$_c$FP-); ^{1}H; DMSO-d6; 700 MHz; 1:0.6 ratio

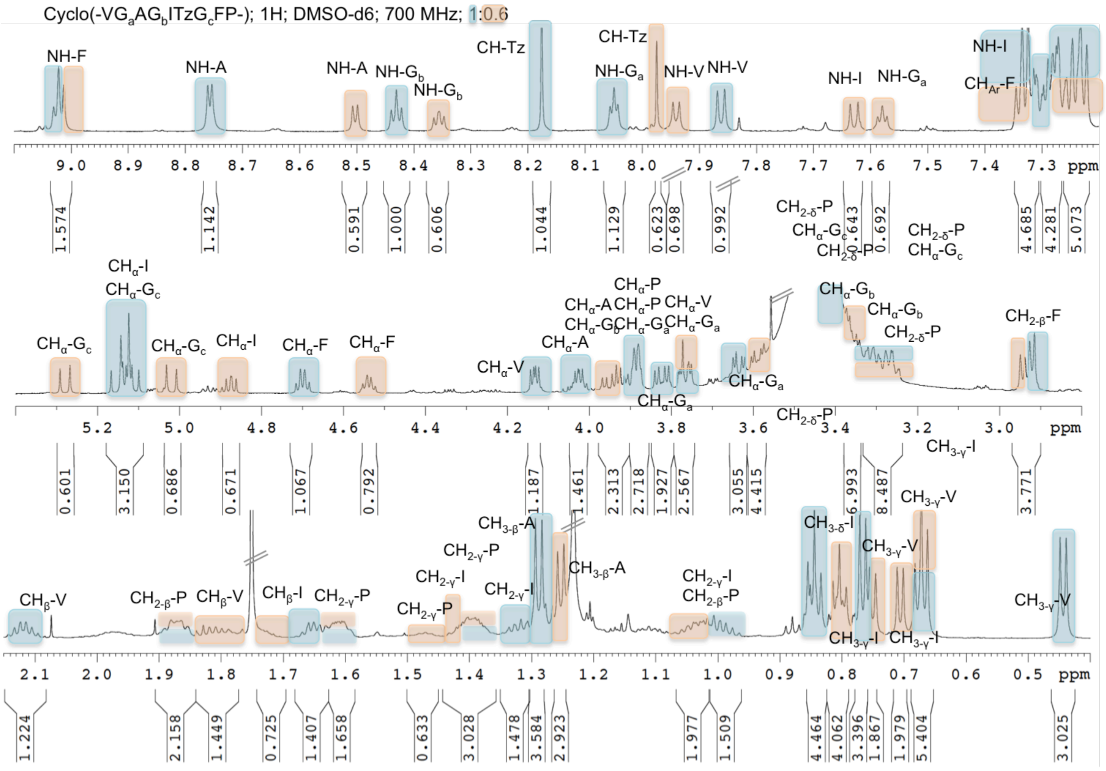

CP5; Cyclo(-VG$_a$AG$_b$ITzG$_c$FP-); HSQC; DMSO-d6; 700 MHz

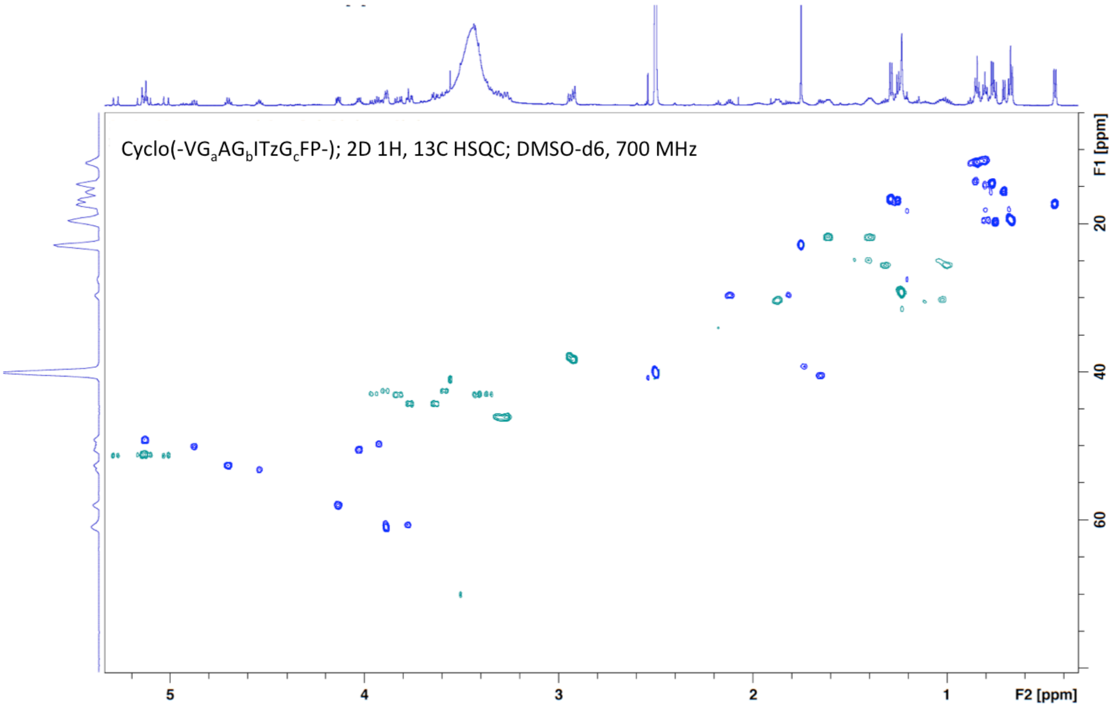

CP6; Cyclo(-VG$_a$AG$_b$IG$_c$TzFP-); ^{1}H; DMSO-d6; 700 MHz; 2:1 ratio

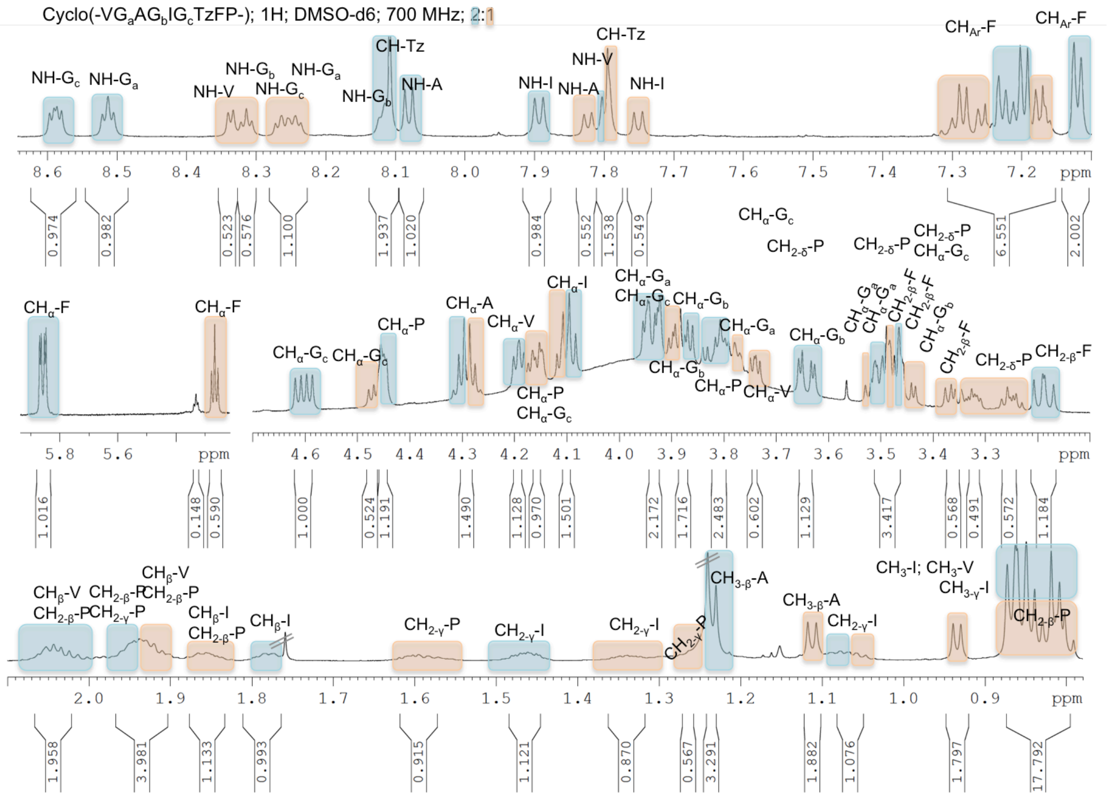

CP6; Cyclo(-VG$_a$AG$_b$IG$_c$TzFP-); HSQC; DMSO-d6; 700 MHz

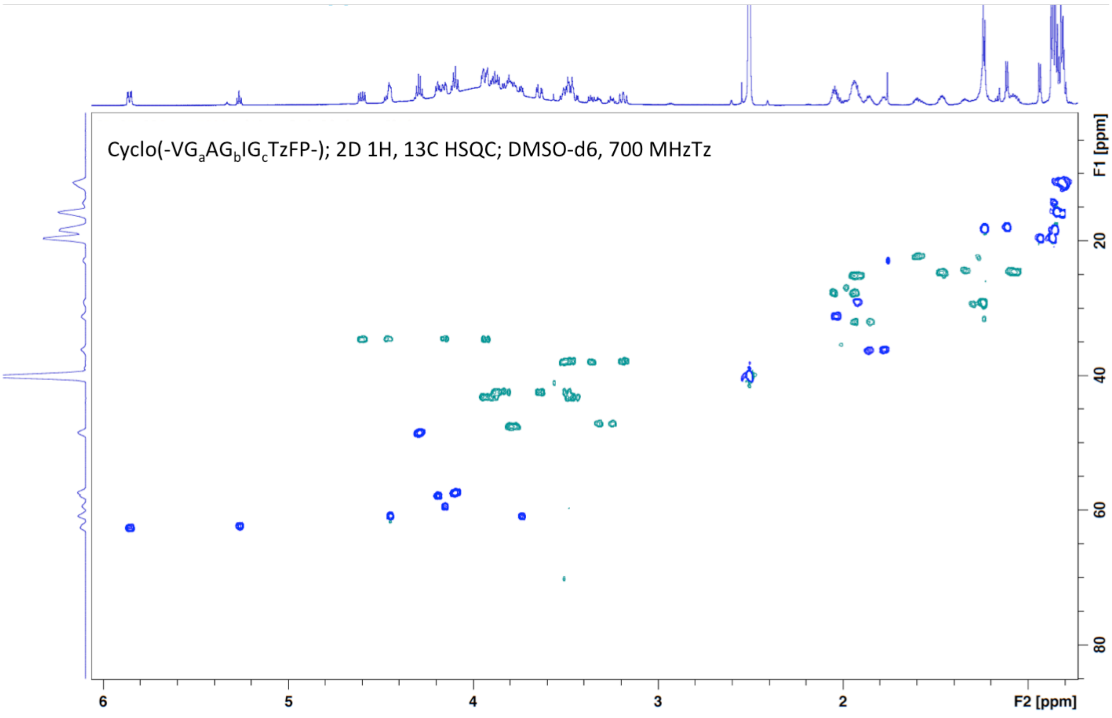

CP13; Cyclo(-VTzG$_a$ATzG$_b$IG$_c$FP-); ^{1}H; DMSO-d6; 700 MHz; 3:1 ratio

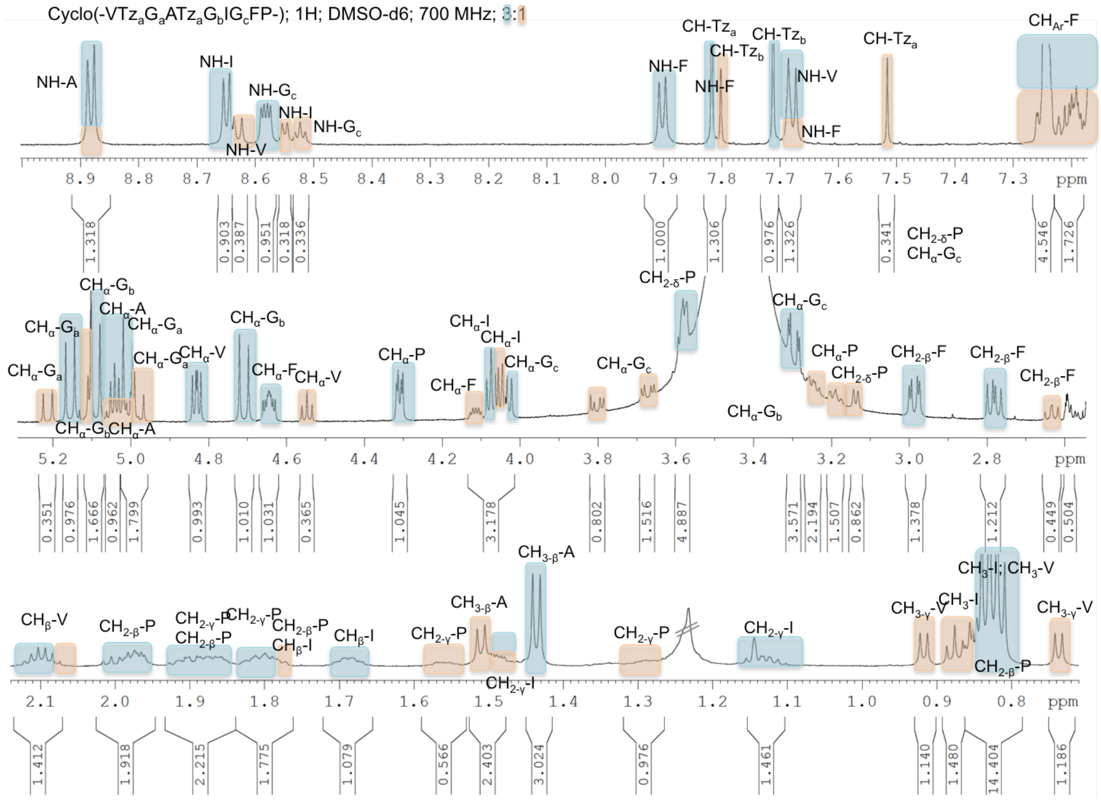

CP13; Cyclo(-VTzG$_a$ATzG$_b$IG$_c$FP-); HSQC; DMSO-d6; 700 MHz

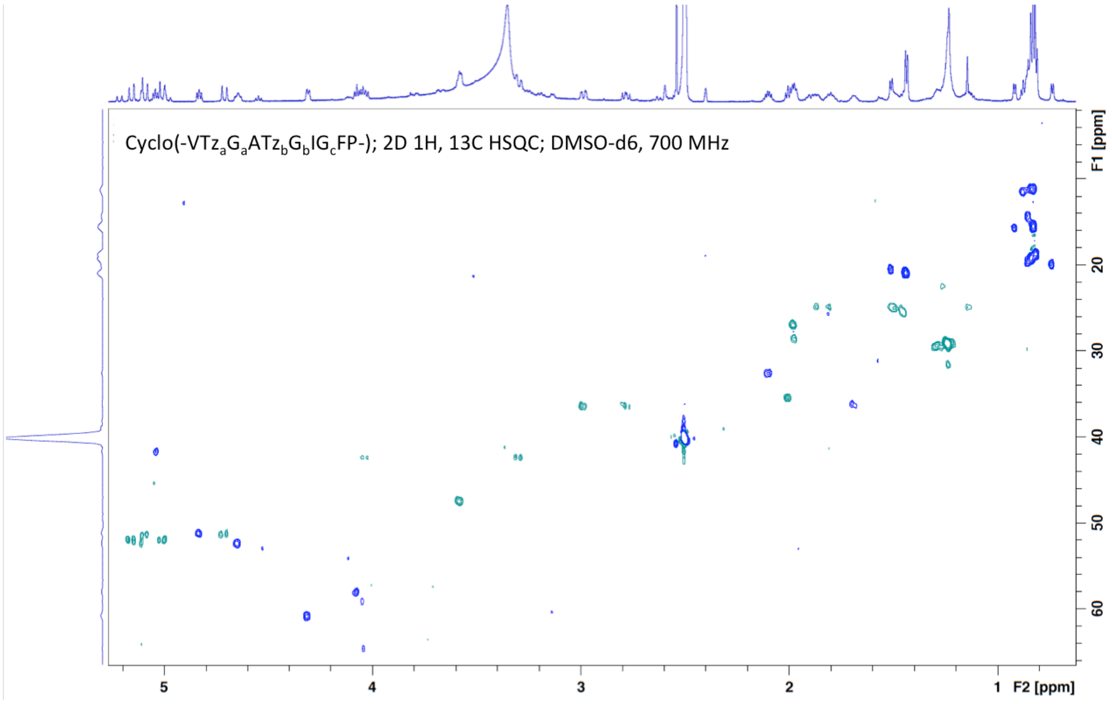

CP135; Cyclo(-VTzG$_a$ATzG$_b$ITzG$_c$FP-); ^{1}H; DMSO-d6; 700 MHz; 1:1.6:1.1:x ratio*

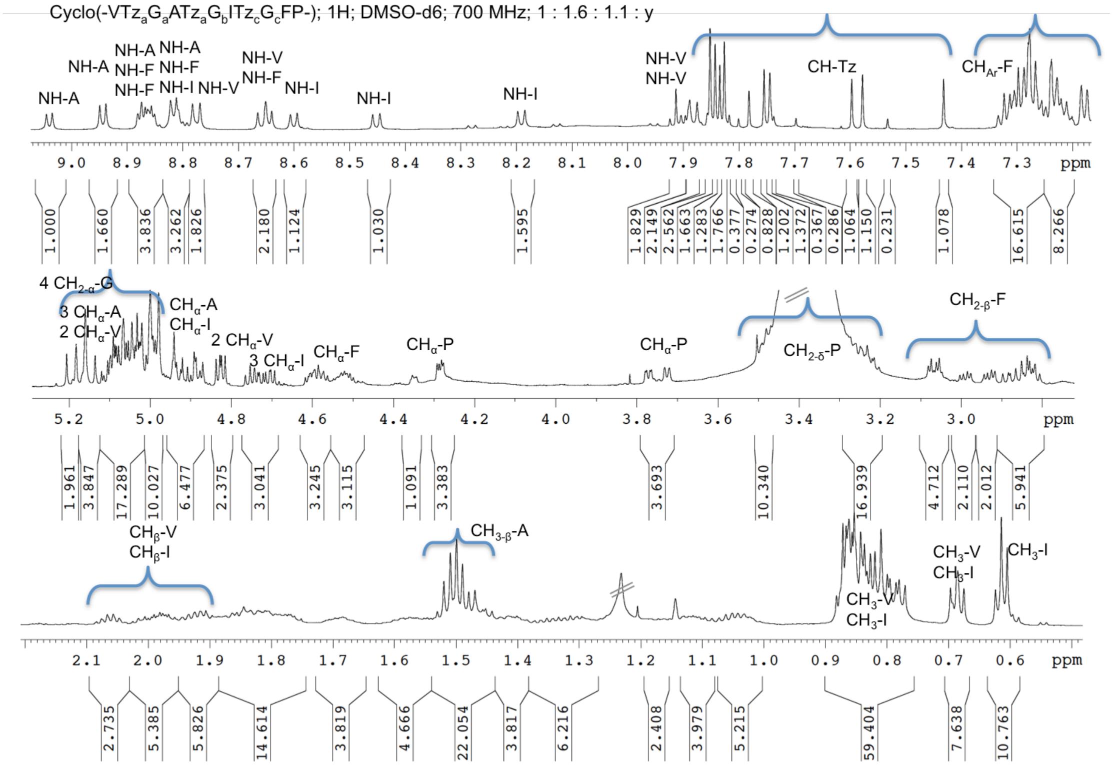

The full ratio of all four major species is not determined. The individual sequences of all four major species are not determined.

CP135; Cyclo(-VTzG$_a$ATzG$_b$ITzG$_c$FP-); HSQC; DMSO-d6; 700 MHz

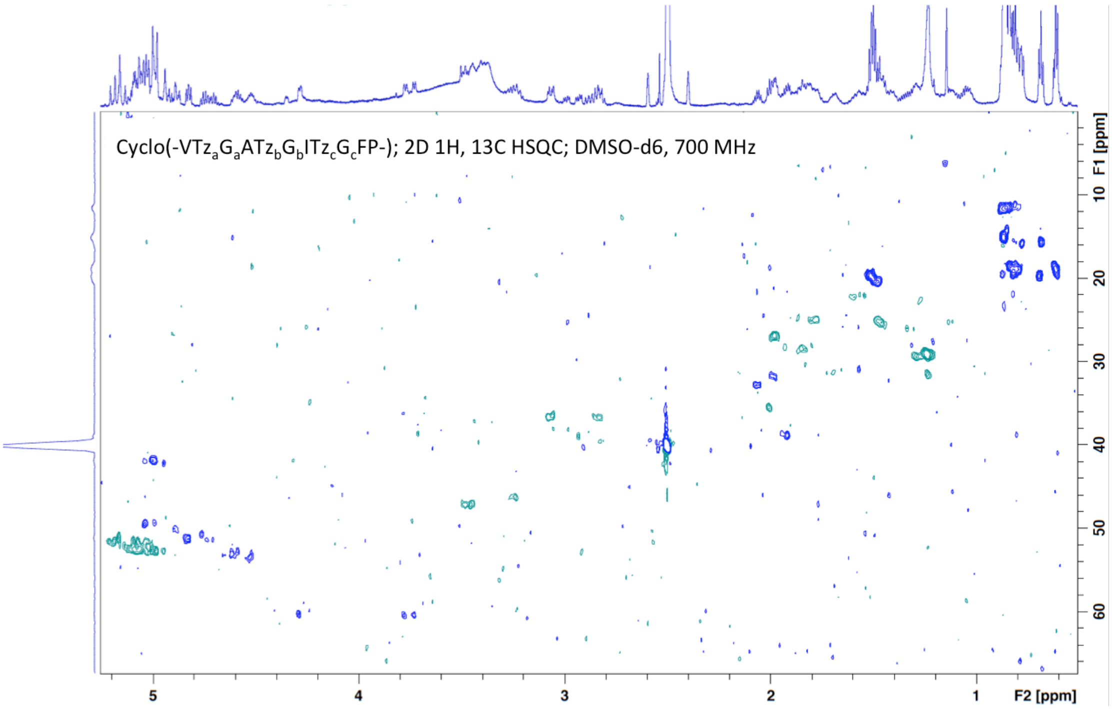

VII. EXSY NMR of final cyclic peptides

CP0; Cyclo(-VG$_a$AG$_b$IG$_c$FP-); EXSY

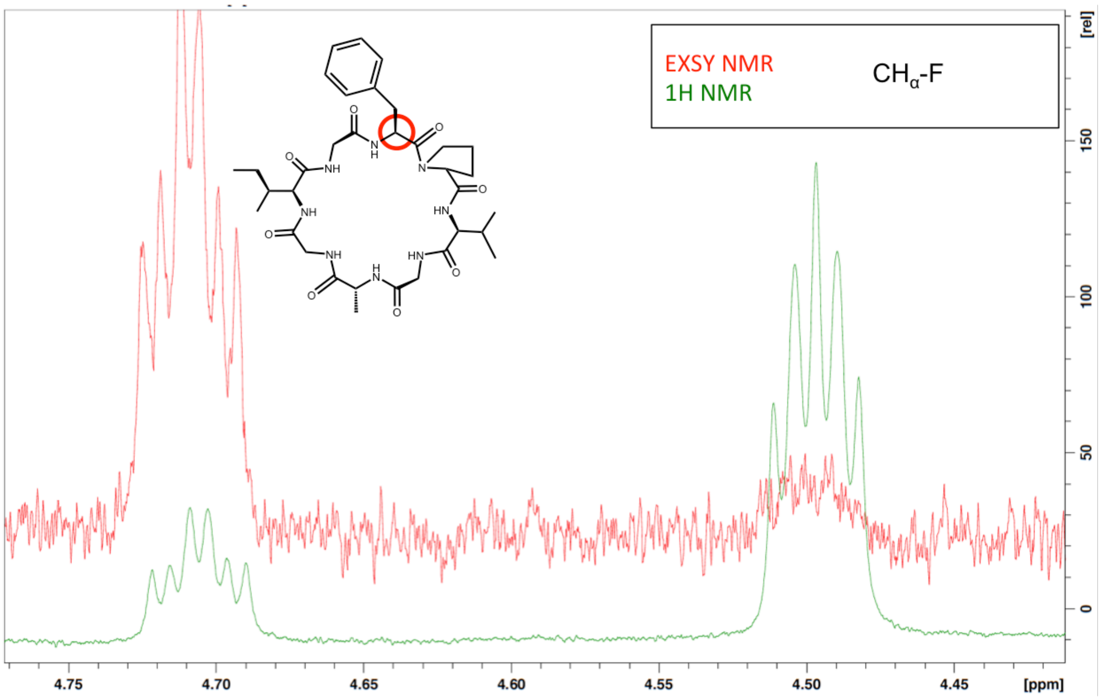

CP1; Cyclo(-VTzG$_a$AG$_b$IG$_c$FP-); EXSY

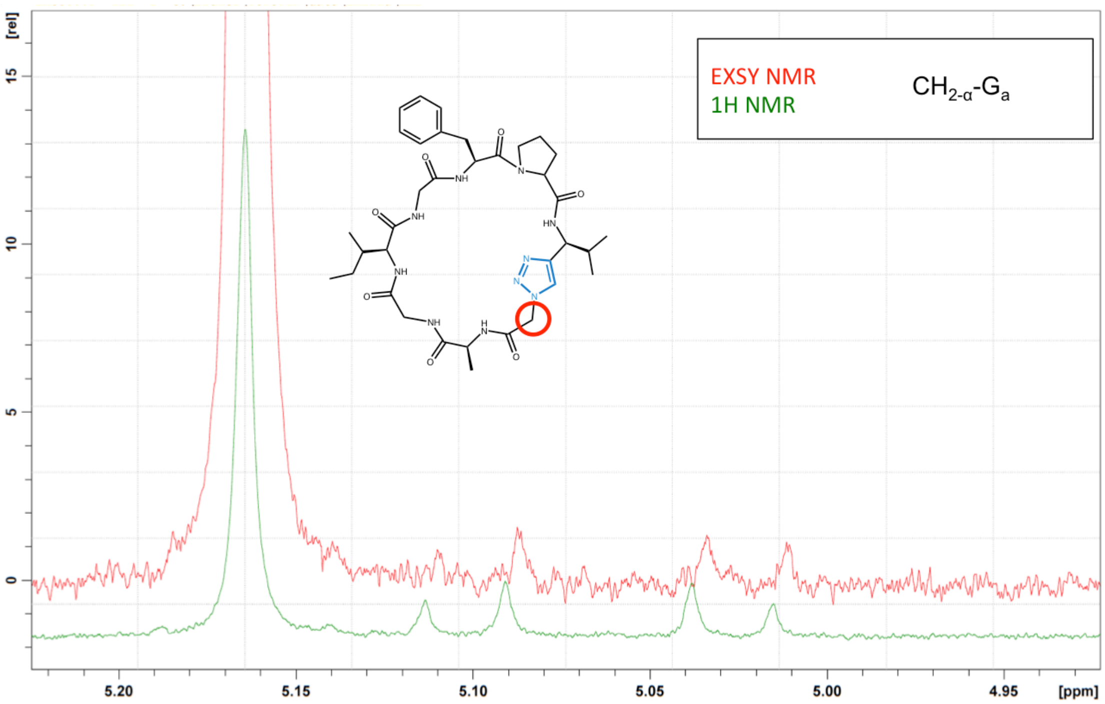

CP2; Cyclo(-VG$_a$TzAG$_b$IG$_c$FP-); EXSY

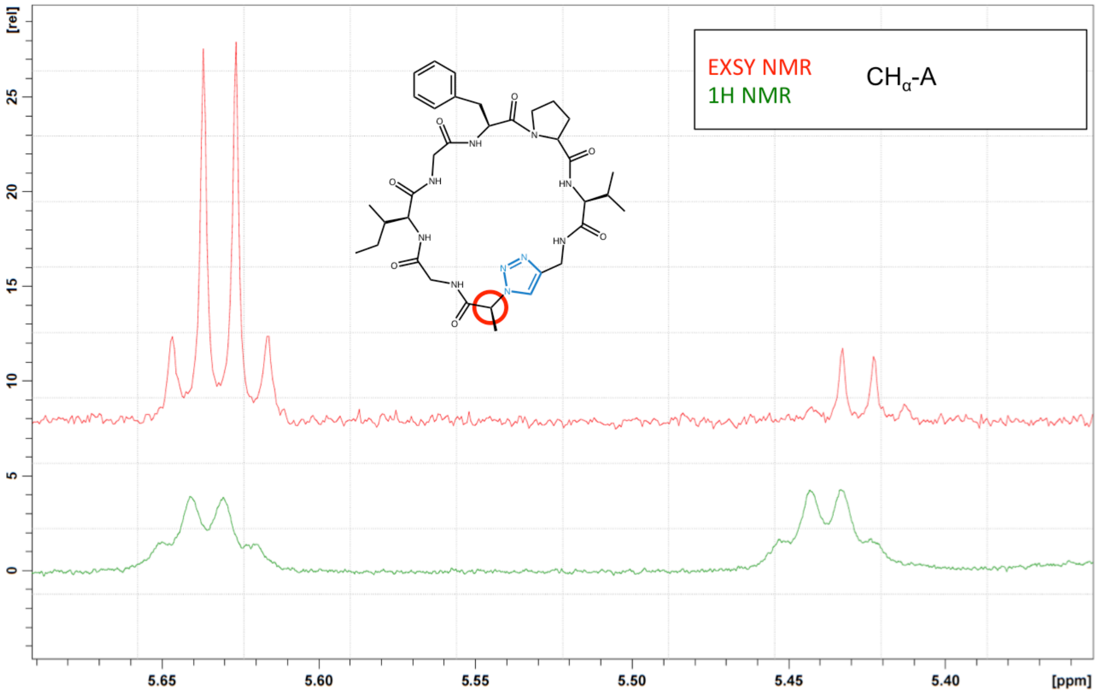

CP3; Cyclo(-VG$_a$ATzG$_b$IG$_c$FP-); EXSY

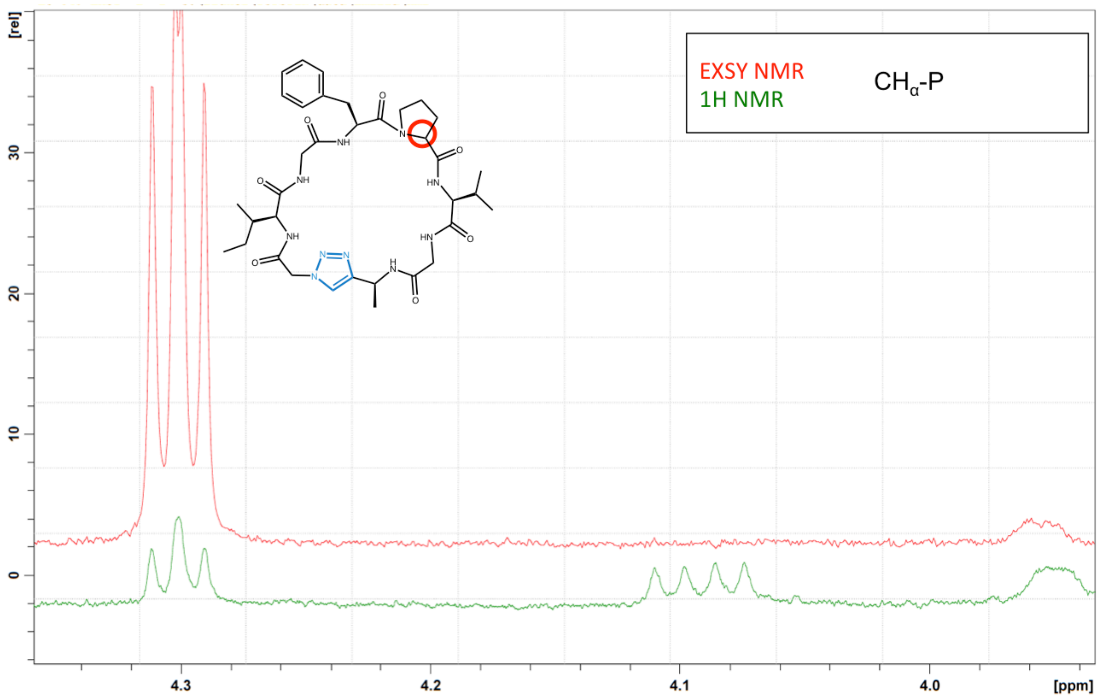

CP4; Cyclo(-VG$_a$AG$_b$TzIG$_c$FP-); EXSY

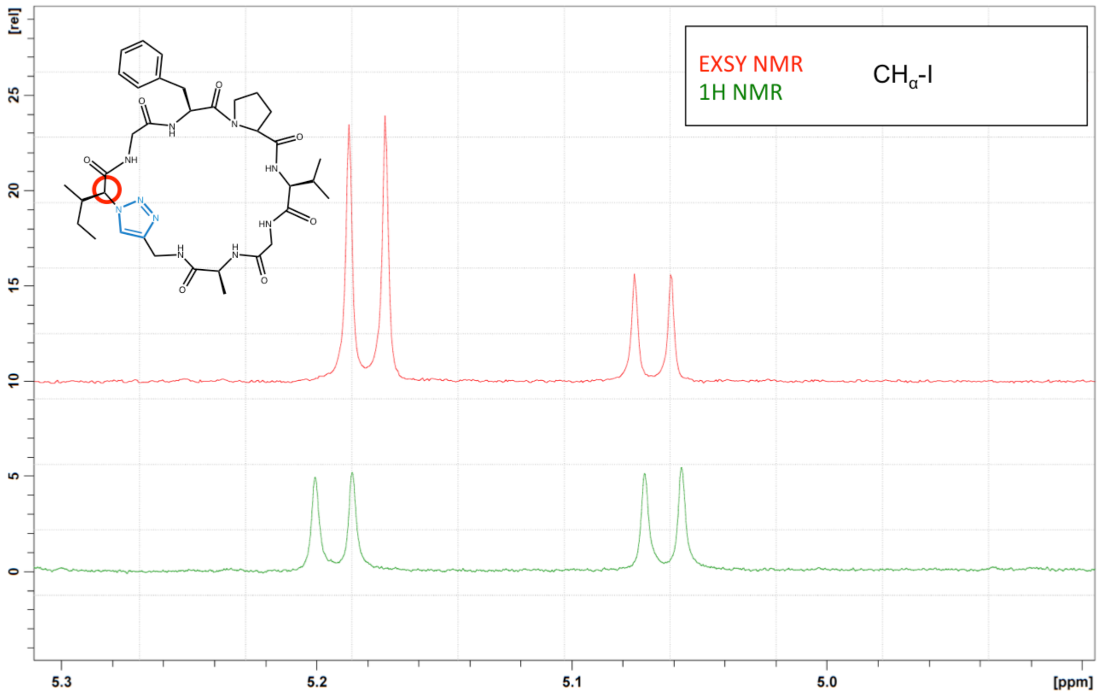

CP5; Cyclo(-VG$_a$AG$_b$ITzG$_c$FP-); EXSY

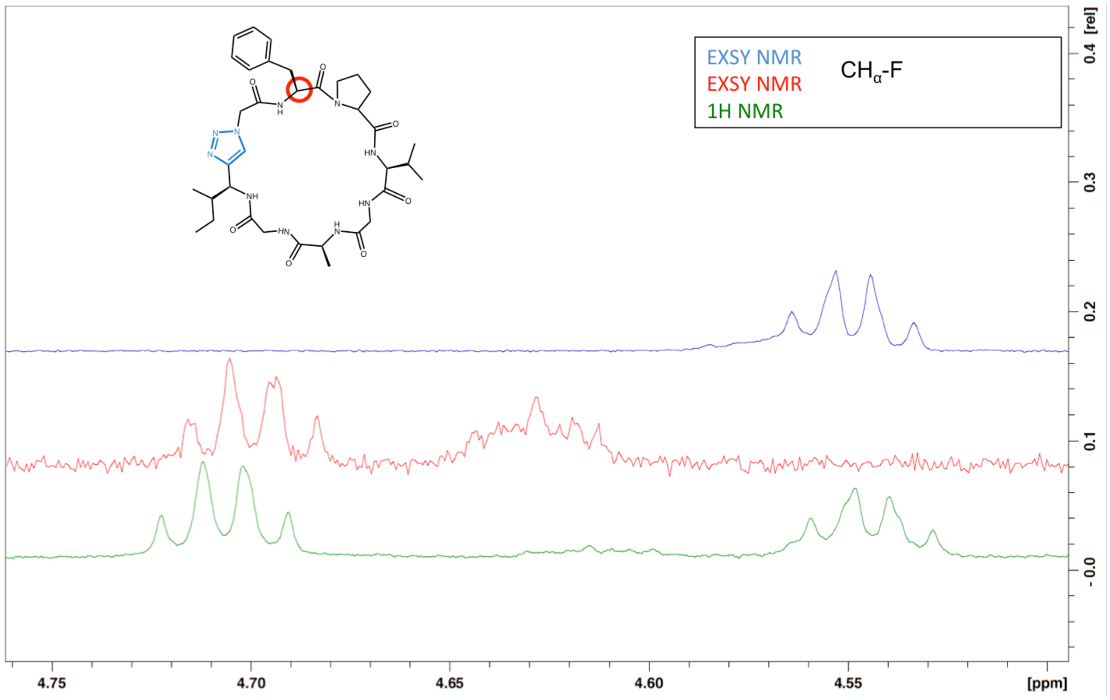

CP6; Cyclo(-VG$_a$AG$_b$IG$_c$TzFP-); EXSY

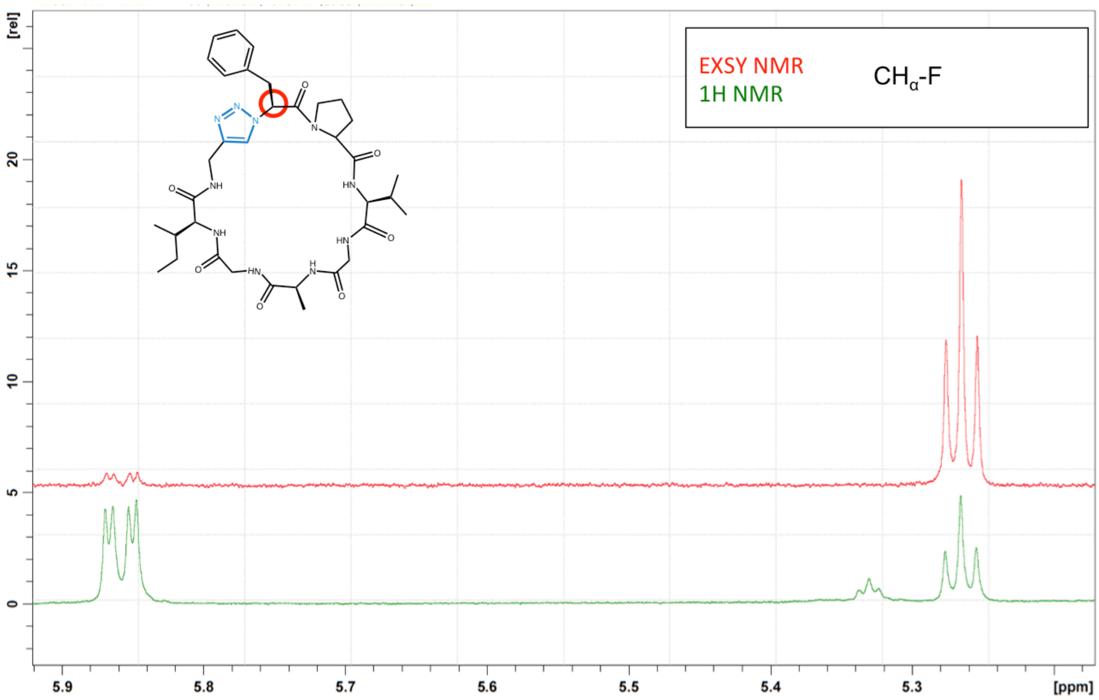

CP13; Cyclo(-VTzG$_a$ATzG$_b$IG$_c$FP-); EXSY

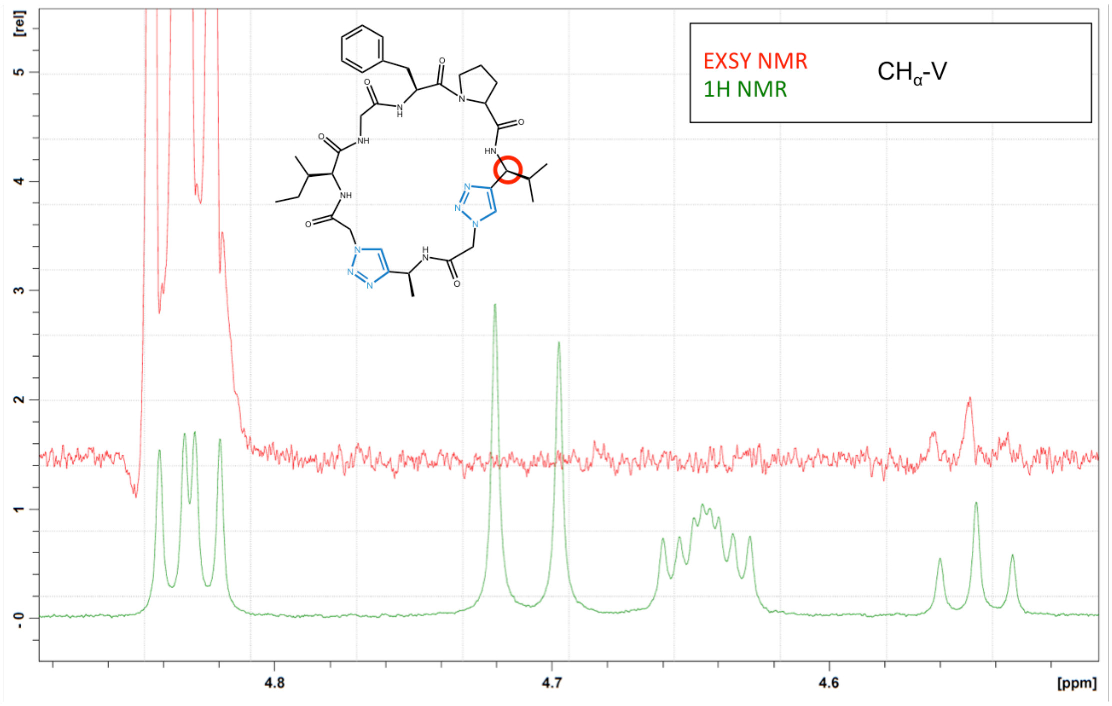

CP135; Cyclo(-VTzG$_a$ATzG$_b$ITzG$_c$FP-); EXSY

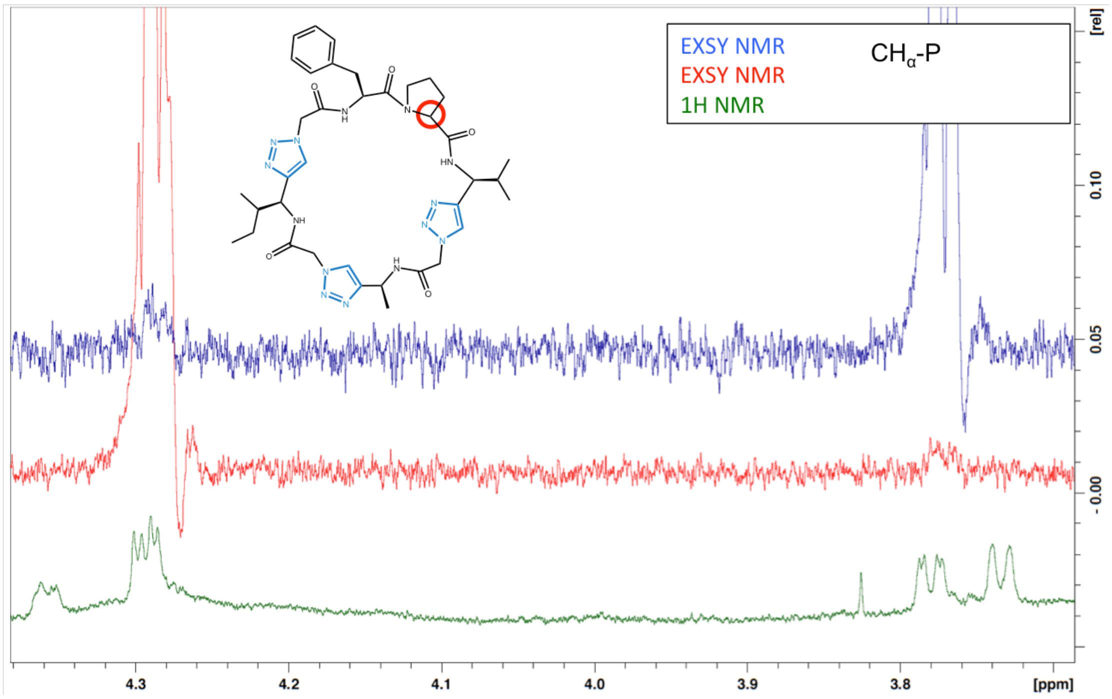

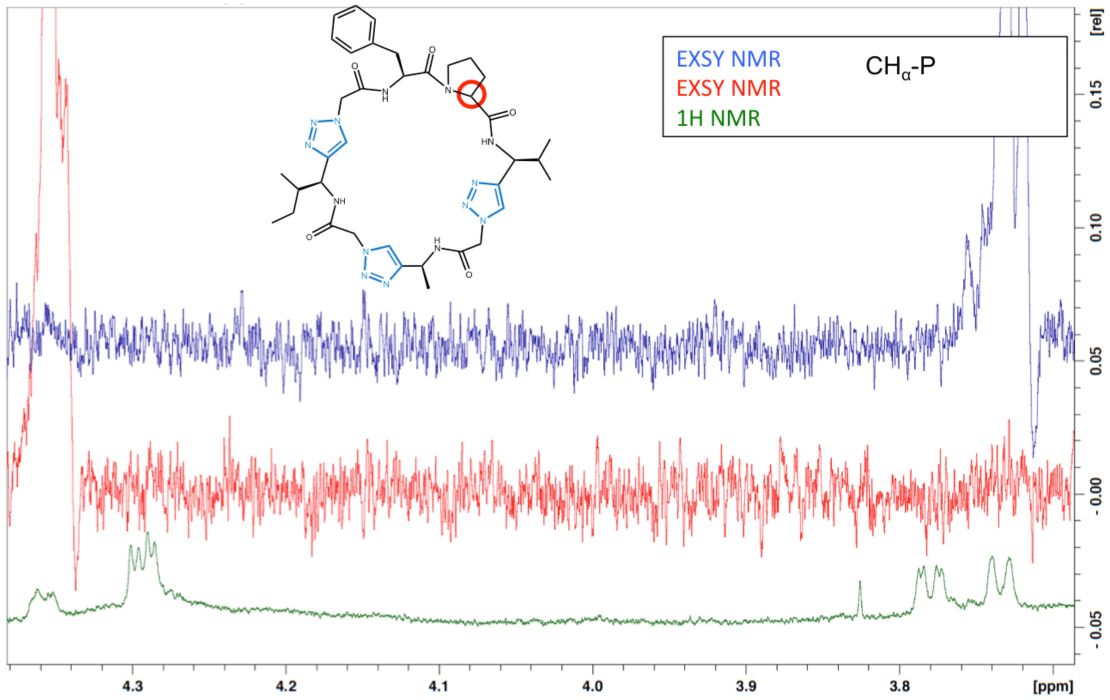

VIII. MS-MS data of final cyclic peptides

CP0; Cyclo(-VG$_a$AG$_b$IG$_c$FP-)

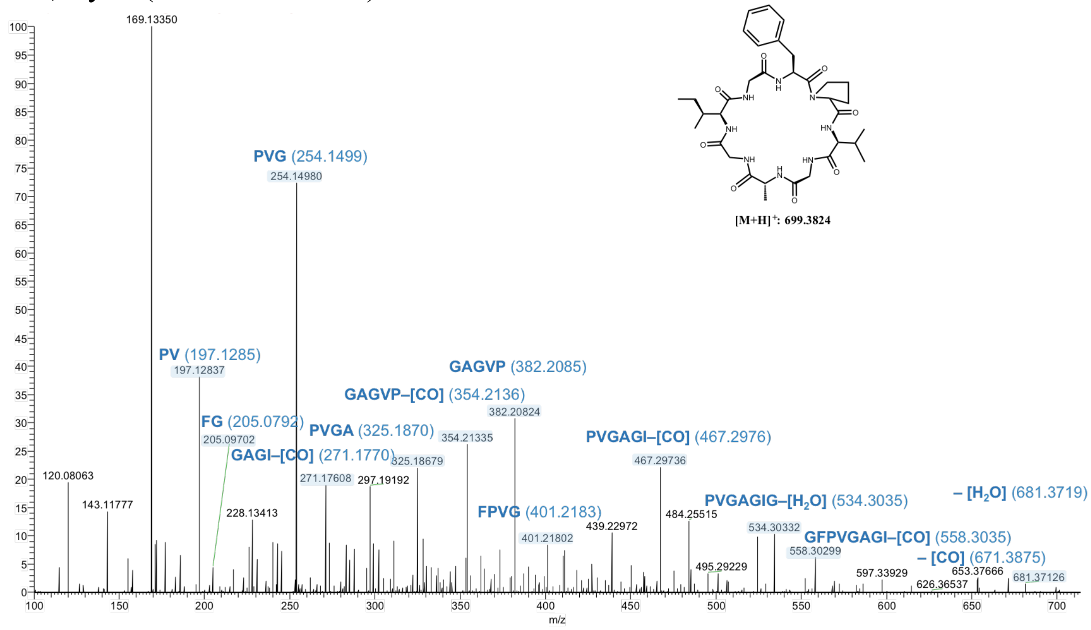

CP1; Cyclo(-VTzG$_a$AG$_b$IG$_c$FP-)

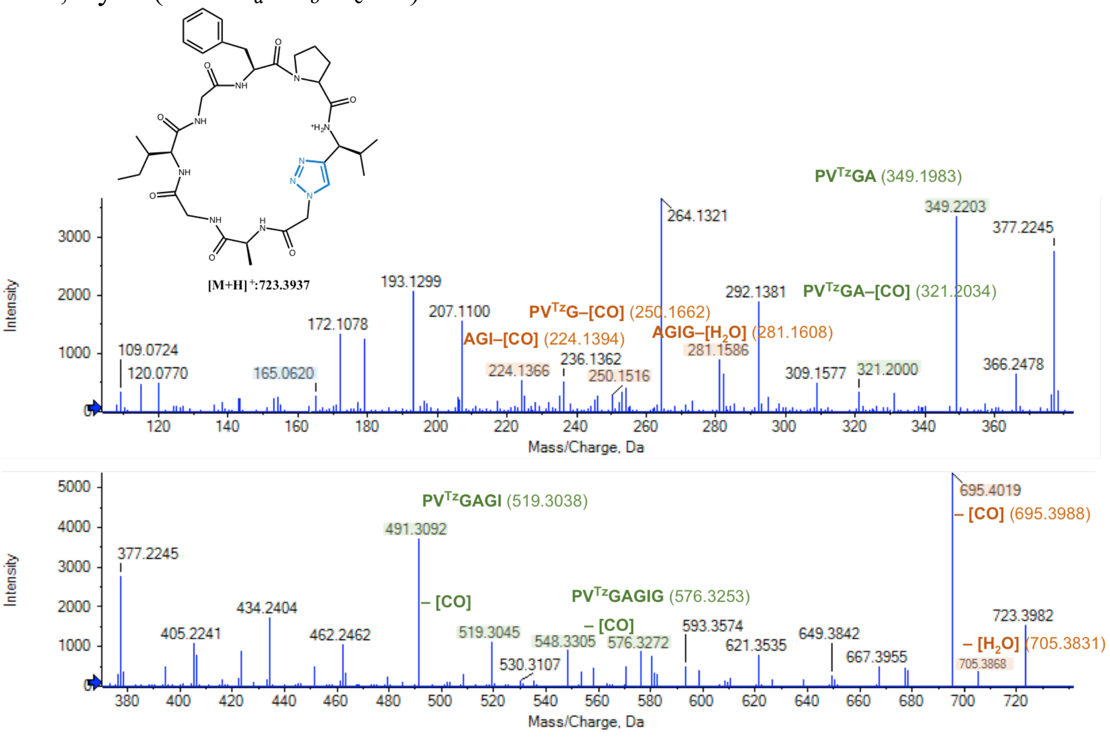

CP2; Cyclo(-VG$_a$TzAG$_b$IG$_c$FP-)

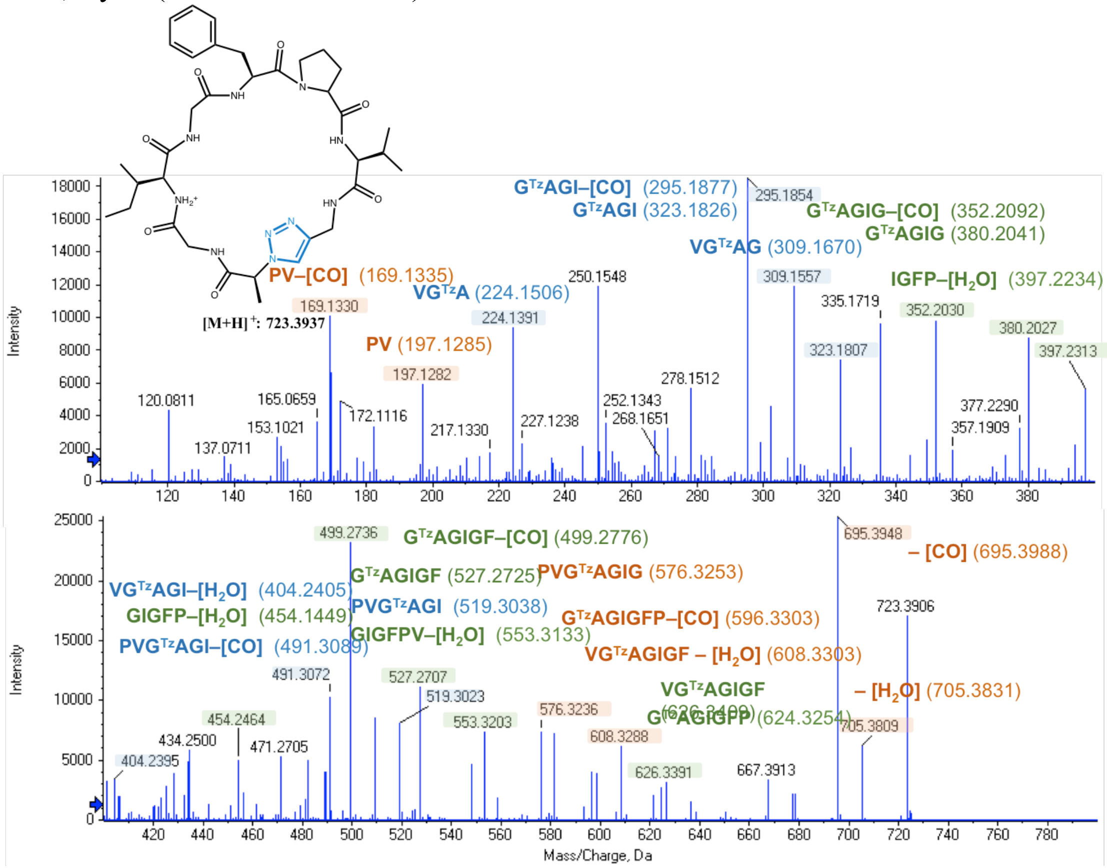

CP3; Cyclo(-VG$_a$ATzG$_b$IG$_c$FP-)

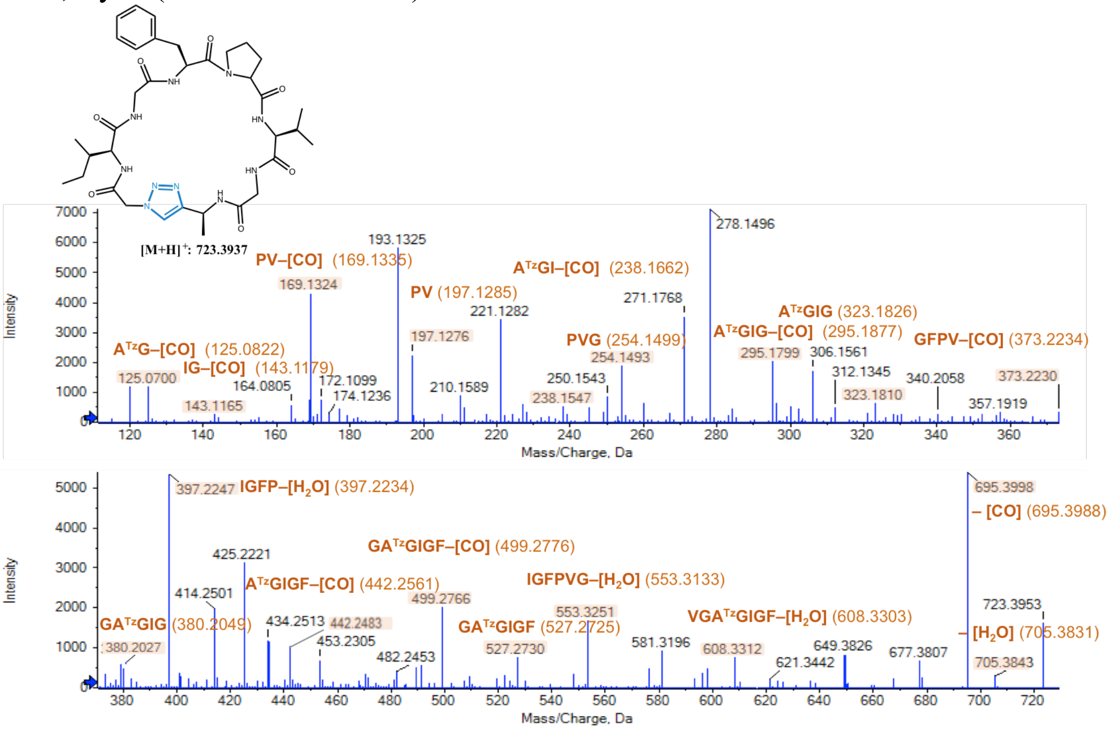

The MS-MS fragmentation data of both cyclic compounds observed in the crude mixture were identical.

CP4; Cyclo(-VG$_a$AG$_b$TzIG$_c$FP-)

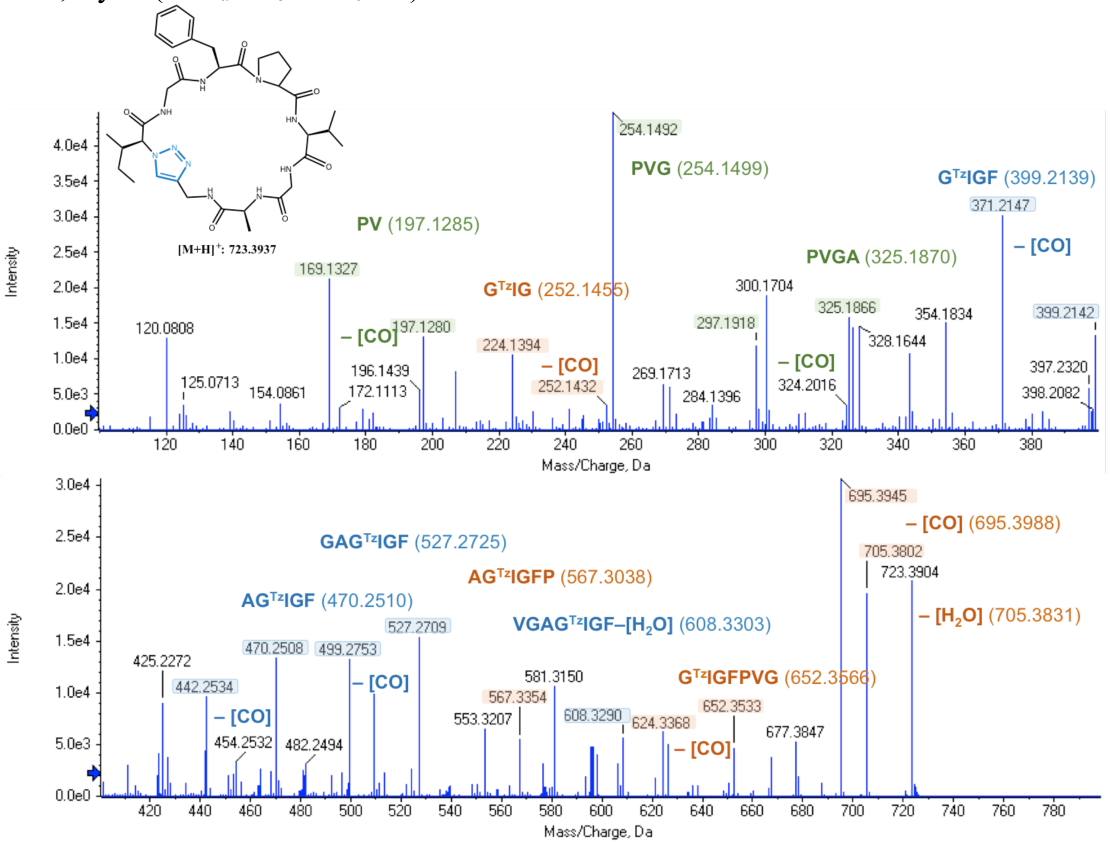

CP5; Cyclo(-VG$_a$AG$_b$ITzG$_c$FP-)

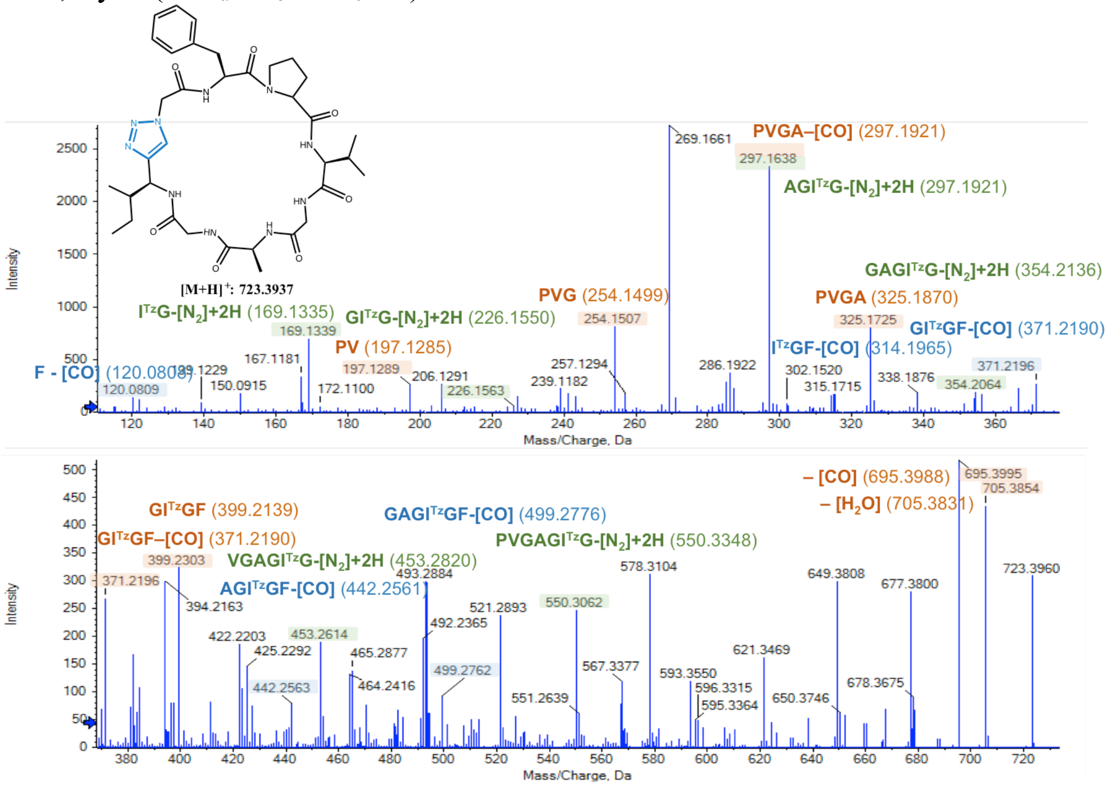

CP6; Cyclo(-VG$_a$AG$_b$IG$_c$TzFP-)

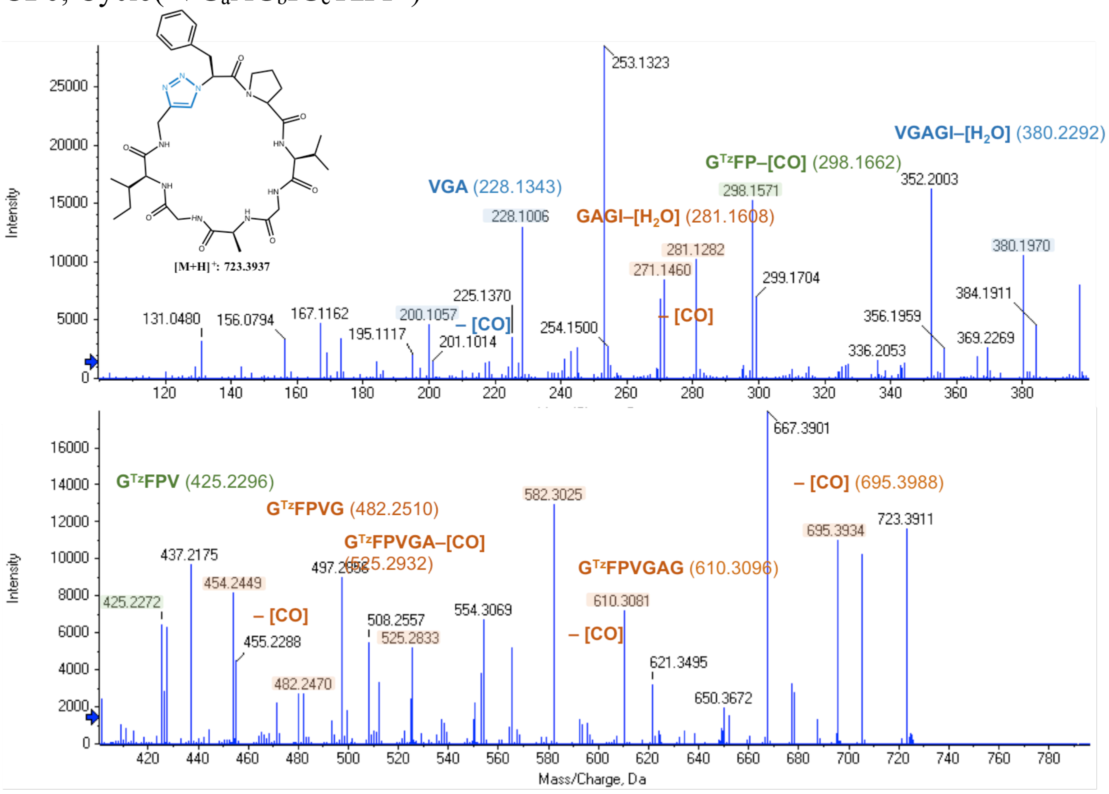

CP13; Cyclo(-VTzG$_a$ATzG$_b$IG$_c$FP-)

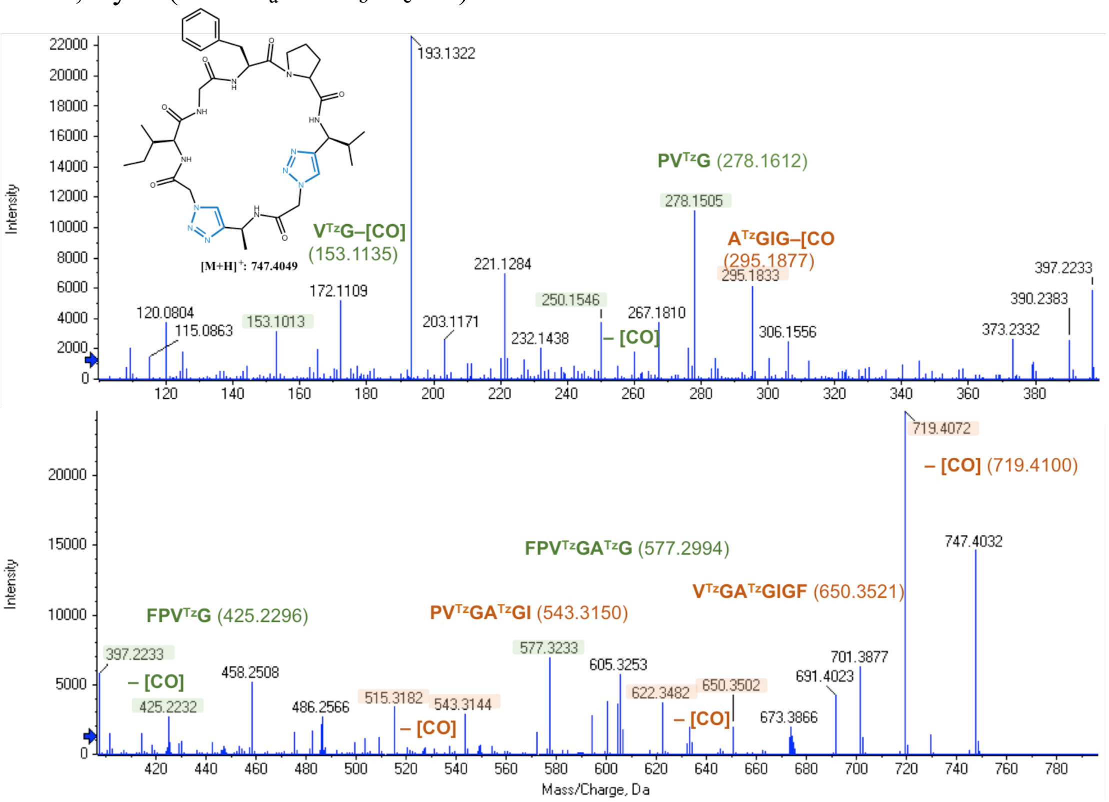

The MS-MS fragmentation data of both cyclic compounds observed in the crude mixture were identical.

CP135; Cyclo(-VTzG$_a$ATzG$_b$ITzG$_c$FP-)

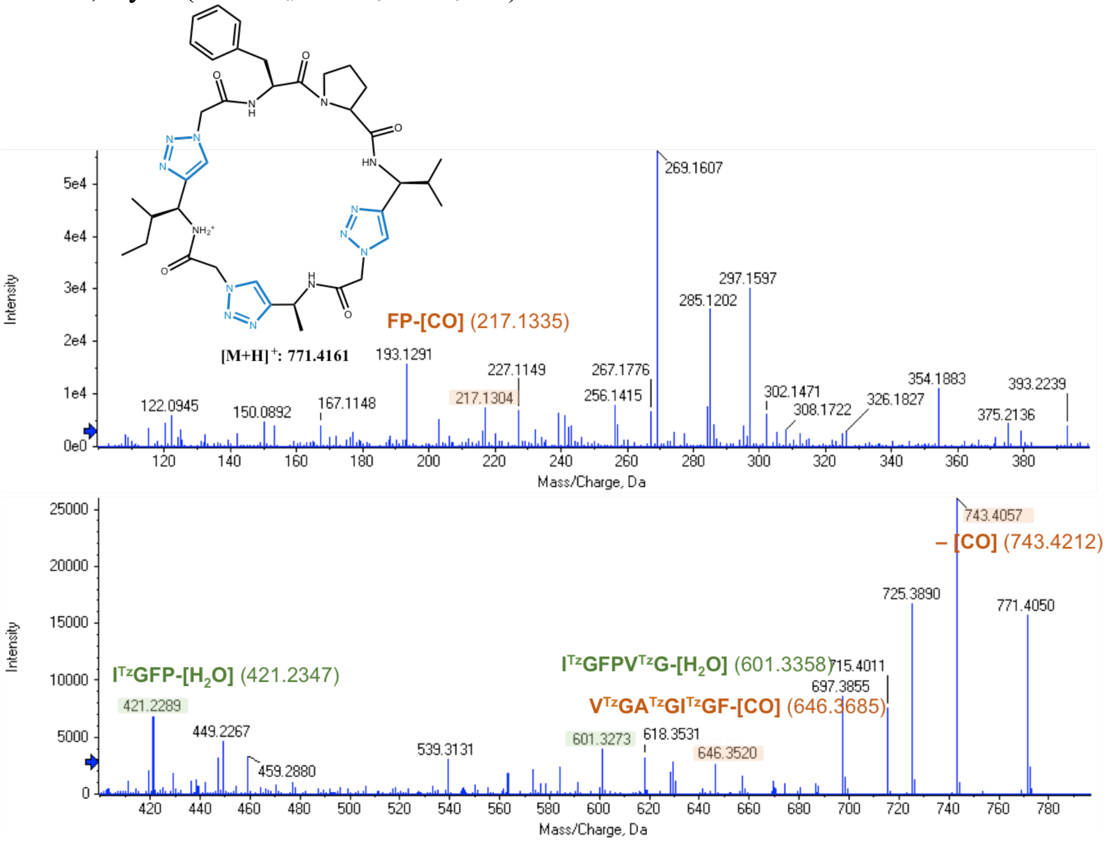

The MS-MS fragmentation data of both cyclic compounds observed in the crude mixture were identical.

IX. LC-MS traces of crude mixtures of CP3, CP13, and CP135

LC-MS trace of the crude reaction mixture of **CP3** showing two peaks with the same desired mass. Both of these peaks were determined to be the cyclic peptide after analysis by HRMS (Table S2) and MS-MS fragmentation (section VIII).

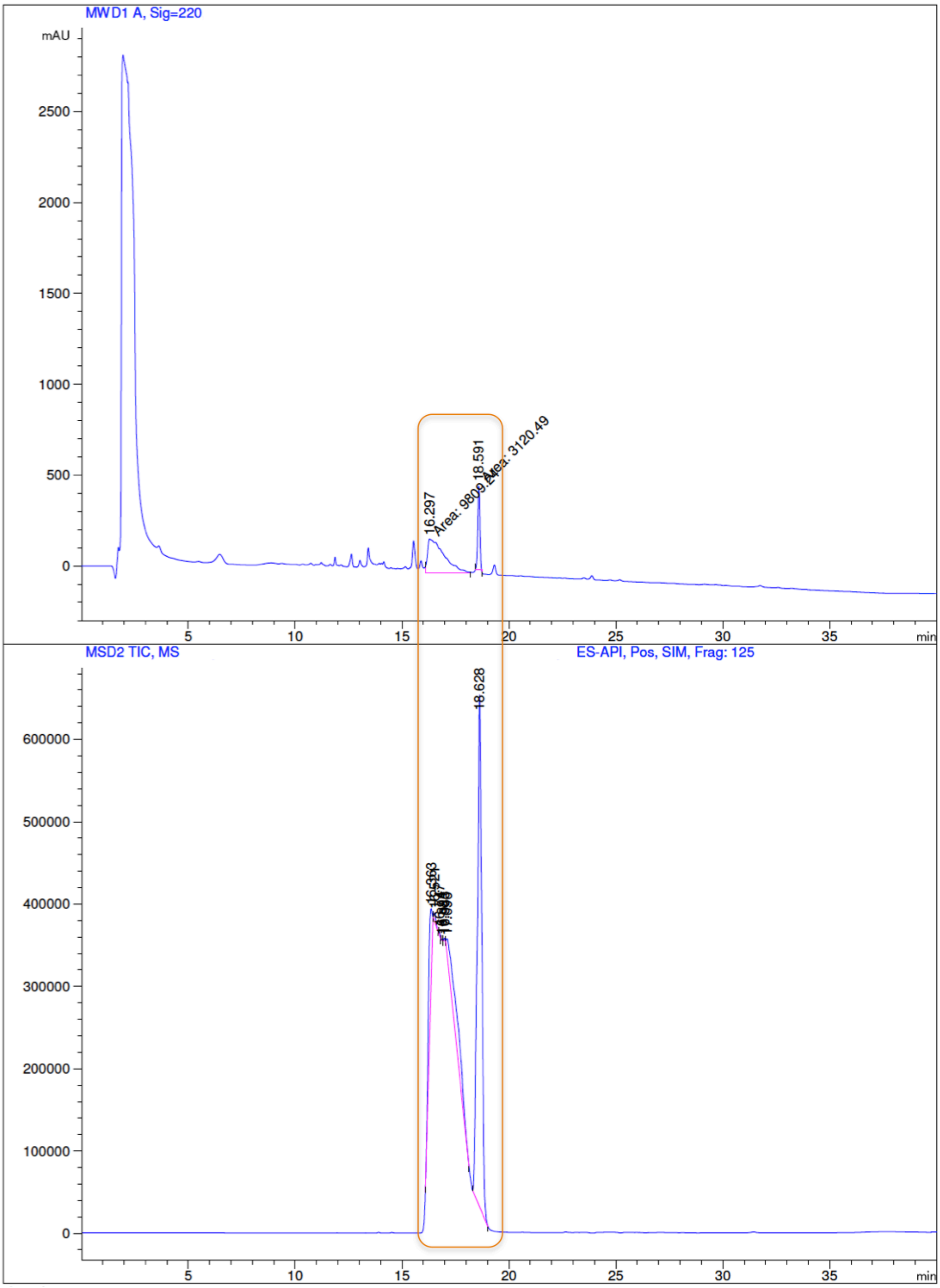

LC-MS trace of the crude reaction mixture of **CP13** showing two peaks with the same desired mass. Both of these peaks were determined to be the cyclic peptide after analysis by HRMS (Table S2) and MS-MS fragmentation (section VIII).

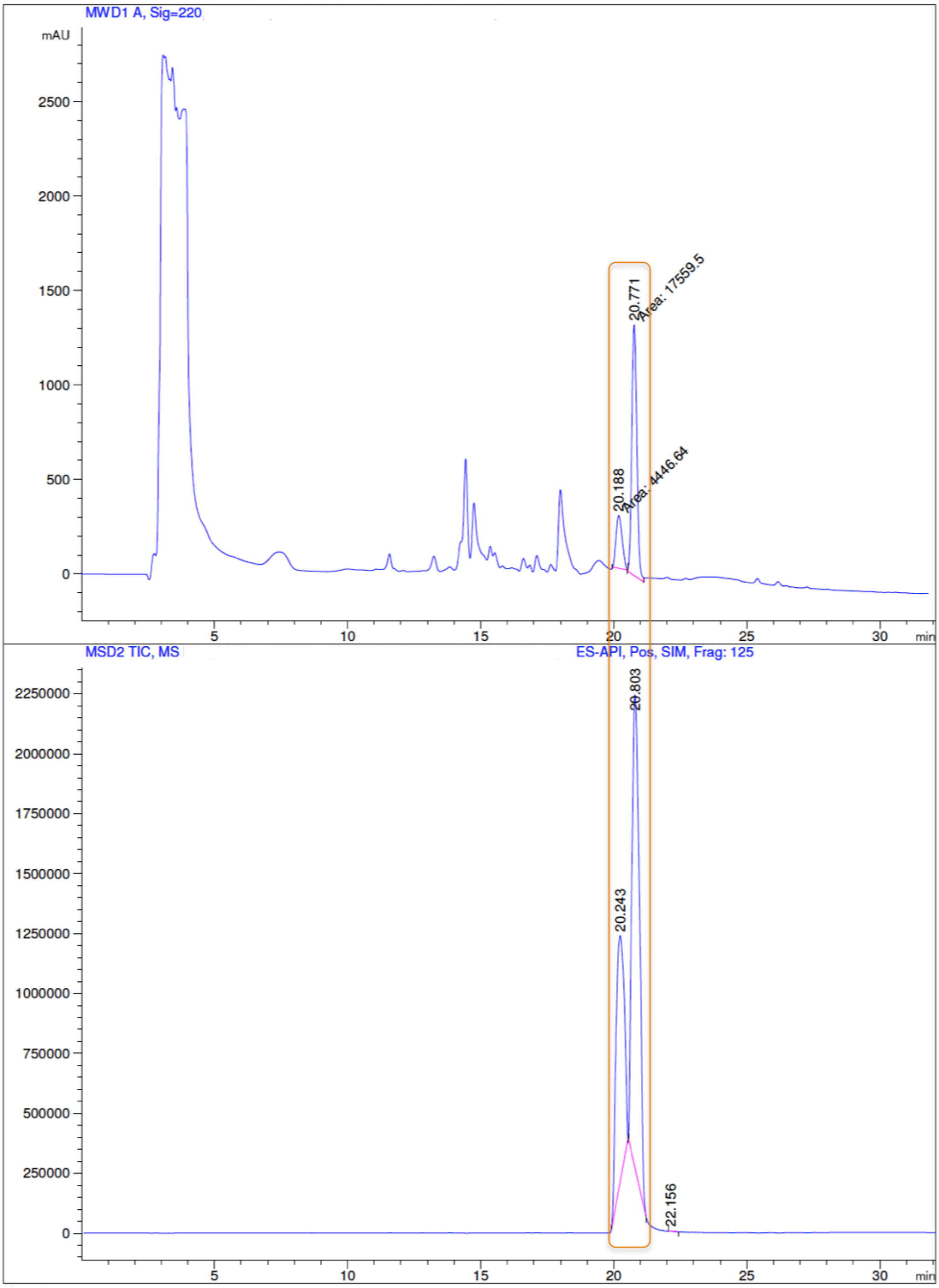

LC-MS trace of the crude reaction mixture of **CP135** showing two peaks with the same desired mass. Both of these peaks were determined to be the cyclic peptide after analysis by HRMS (Table S2) and MS-MS fragmentation (section VIII).

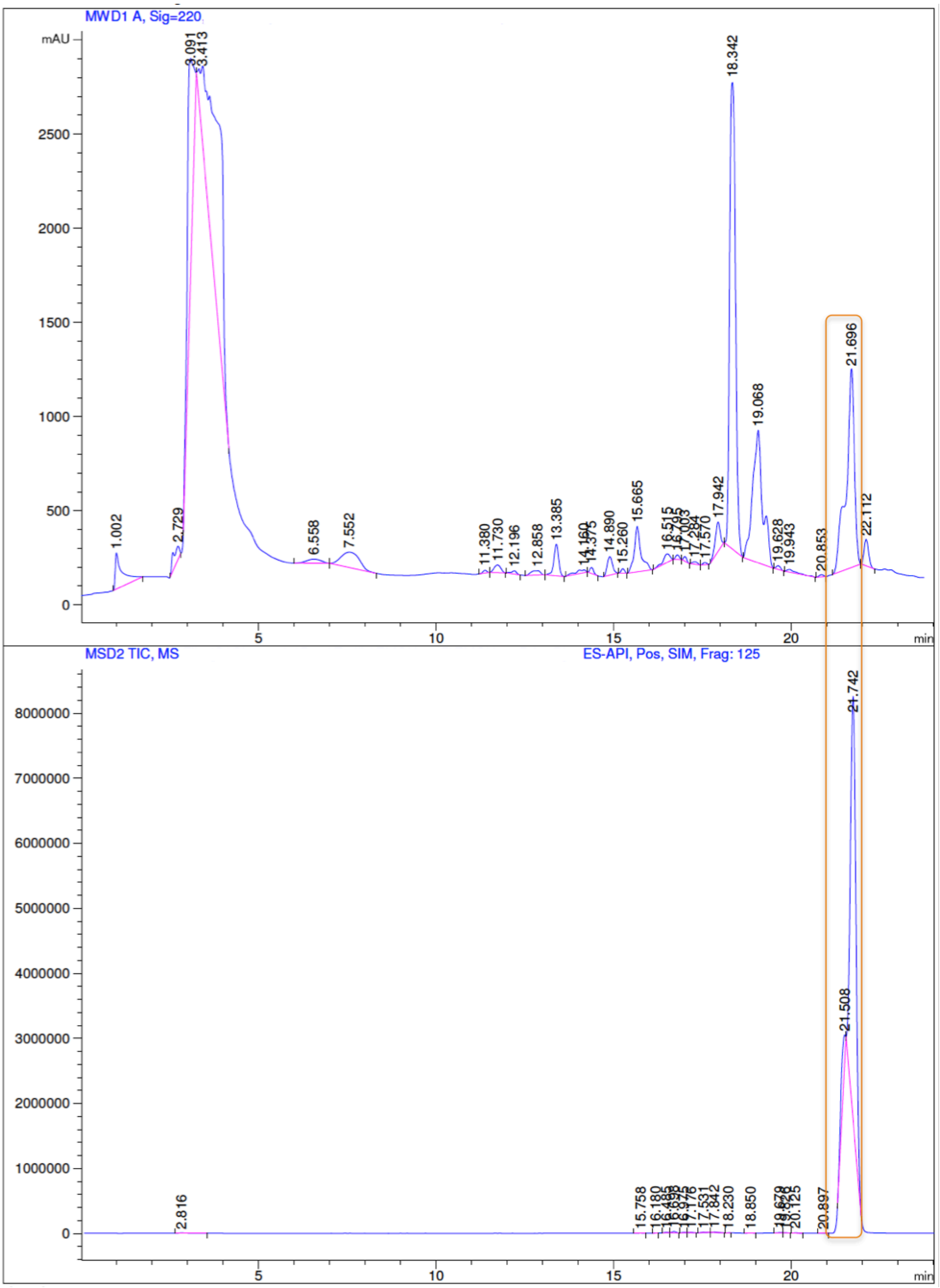

X. LC-MS traces of final cyclic peptides

For each of the cyclic peptides **CP**, the UV trace at 220 nm obtained by HPLC is complemented by its corresponding LCMS trace at the desired molecular weight (Single Ion Monitoring SIM mode) The HPLC methods used are described in section I.

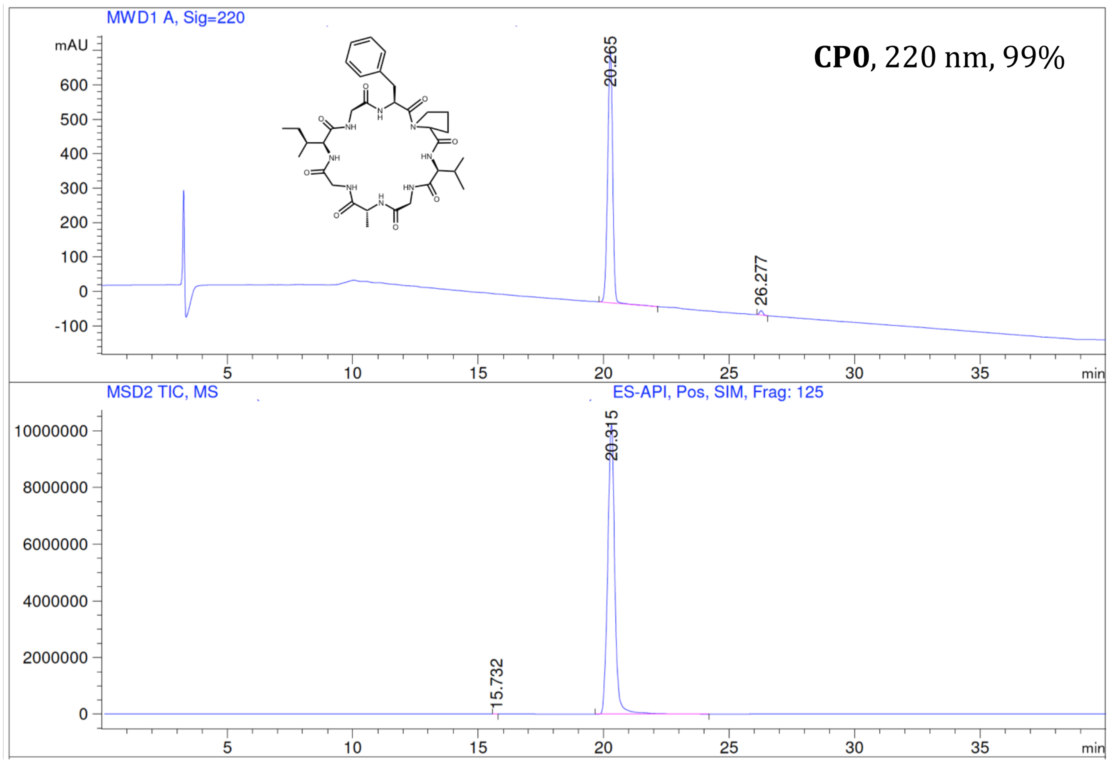

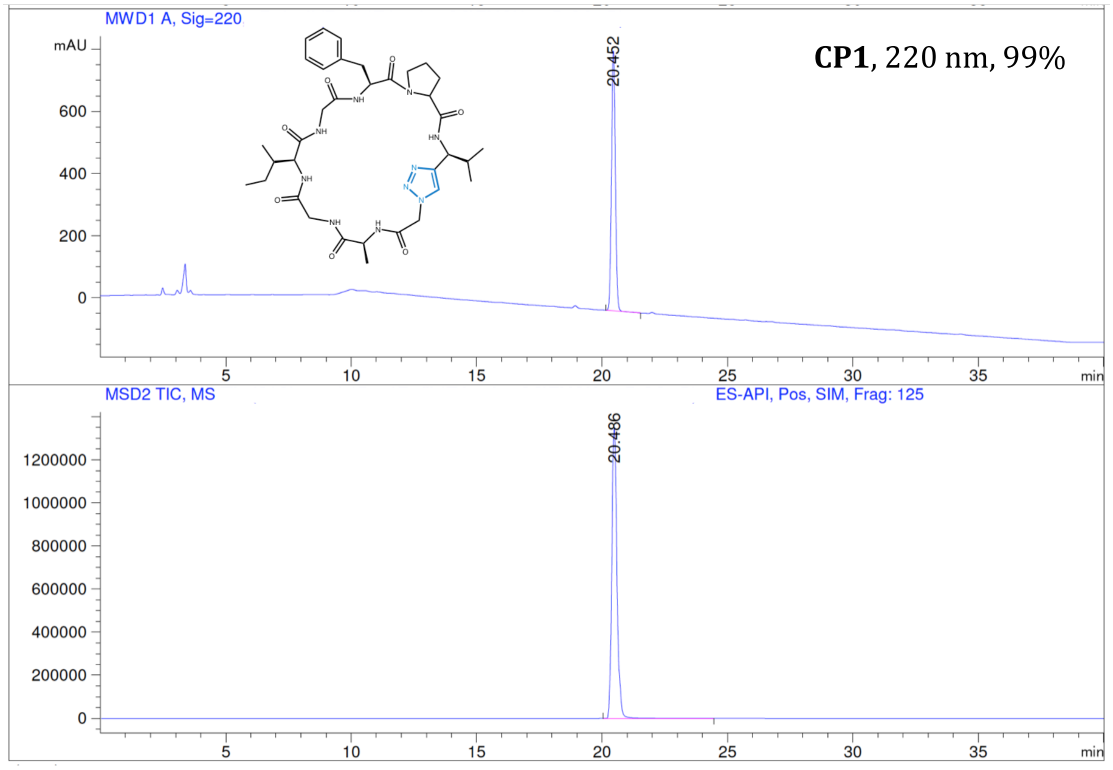

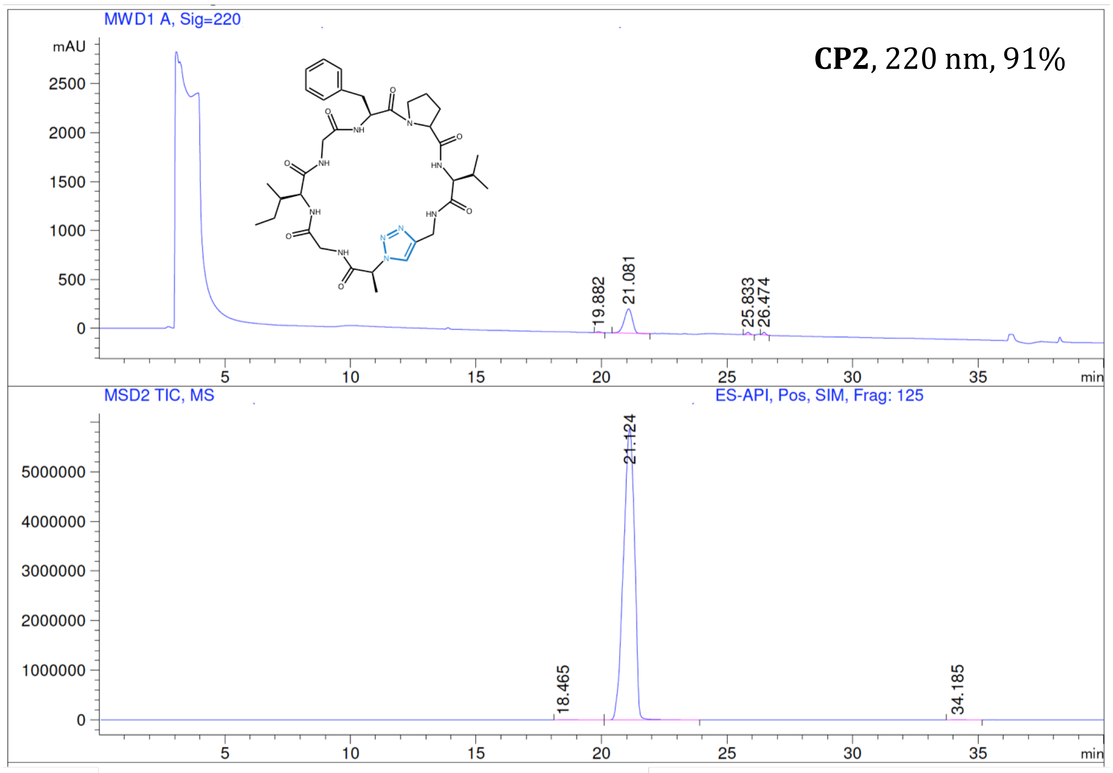
MWD1 A, Sig=220
mAU
CP2, 220 nm, 91%
19.882
21.081
25.833
26.474
min
MSD2 TIC, MS
ES-API, Pos, SIM, Frag: 125
21.124
18.465
34.185

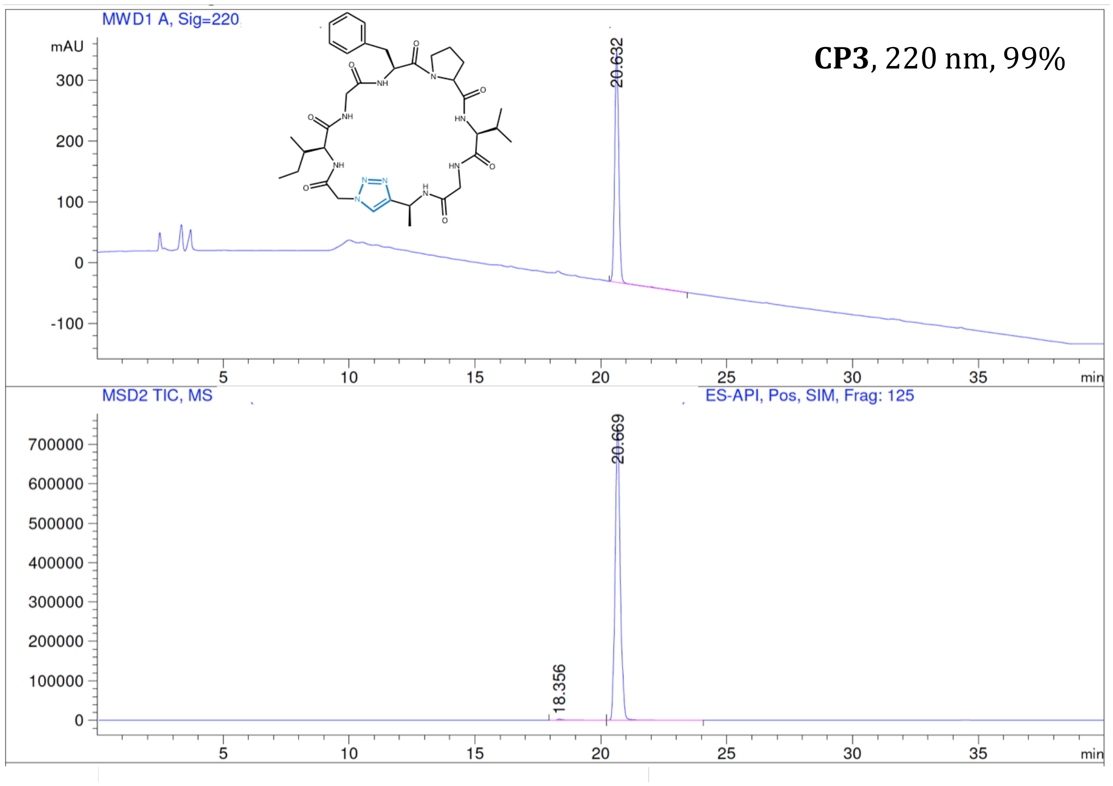
MWD1 A, Sig=220
mAU
CP3, 220 nm, 99%
20.632
min
MSD2 TIC, MS
ES-API, Pos, SIM, Frag: 125
20.669
18.356

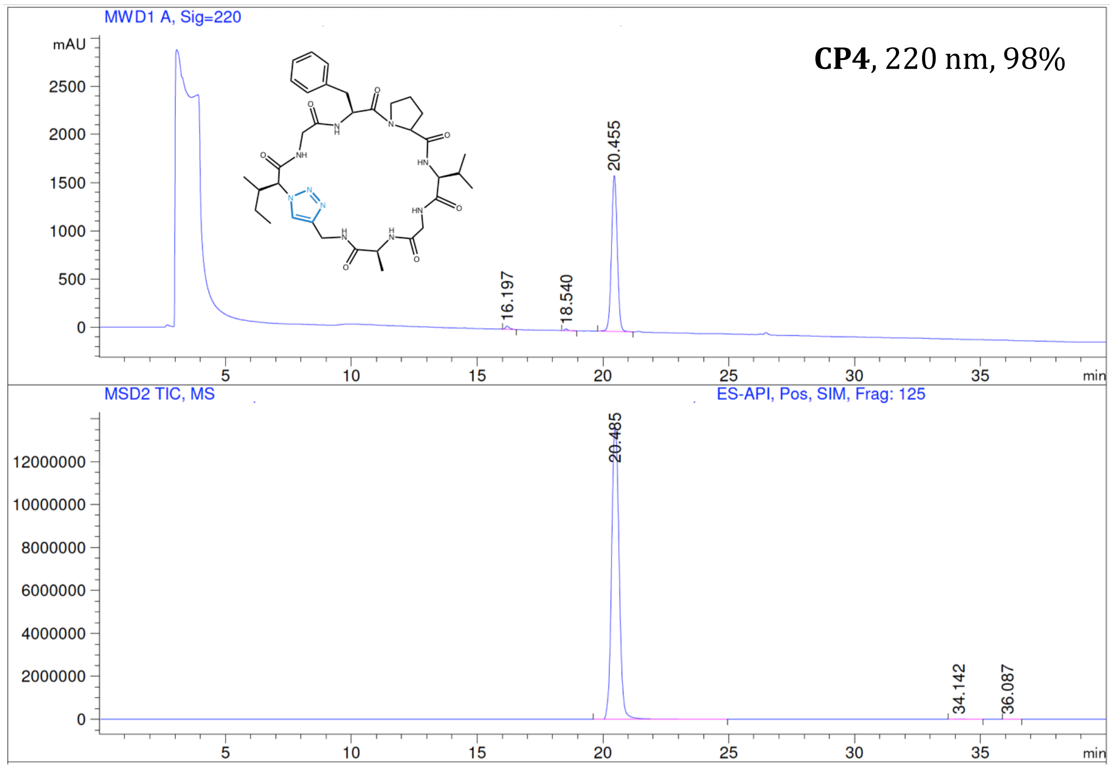
MWD1 A, Sig=220
mAU
2500
2000
1500
1000
500
0
CP4, 220 nm, 98%
16.197
18.540
20.455
5
10
15
20
25
30
35
min
MSD2 TIC, MS
ES-API, Pos, SIM, Frag: 125
12000000
10000000
8000000
6000000
4000000
2000000
0
20.485
34.142
36.087

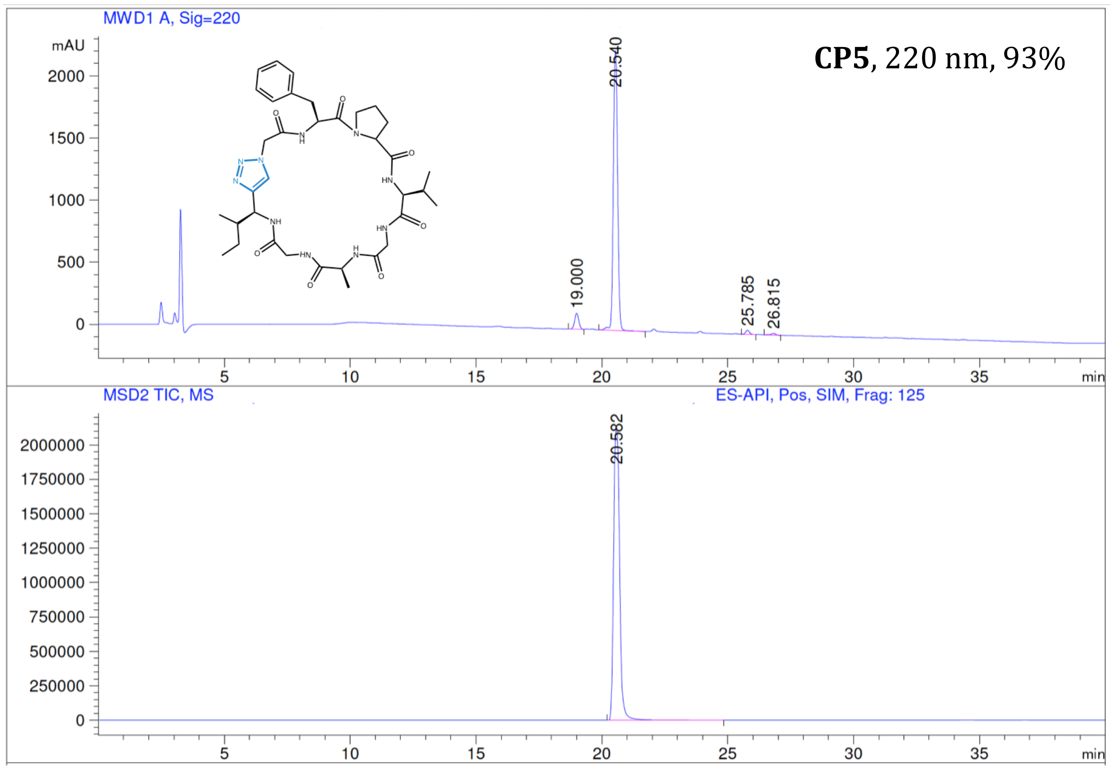
MWD1 A, Sig=220
mAU
2000
1500
1000
500
0
CP5, 220 nm, 93%
19.000
20.540
25.785
26.815
5
10
15
20
25
30
35
min
MSD2 TIC, MS
ES-API, Pos, SIM, Frag: 125
2000000
1750000
1500000
1250000
1000000
750000
500000
250000
0
20.582

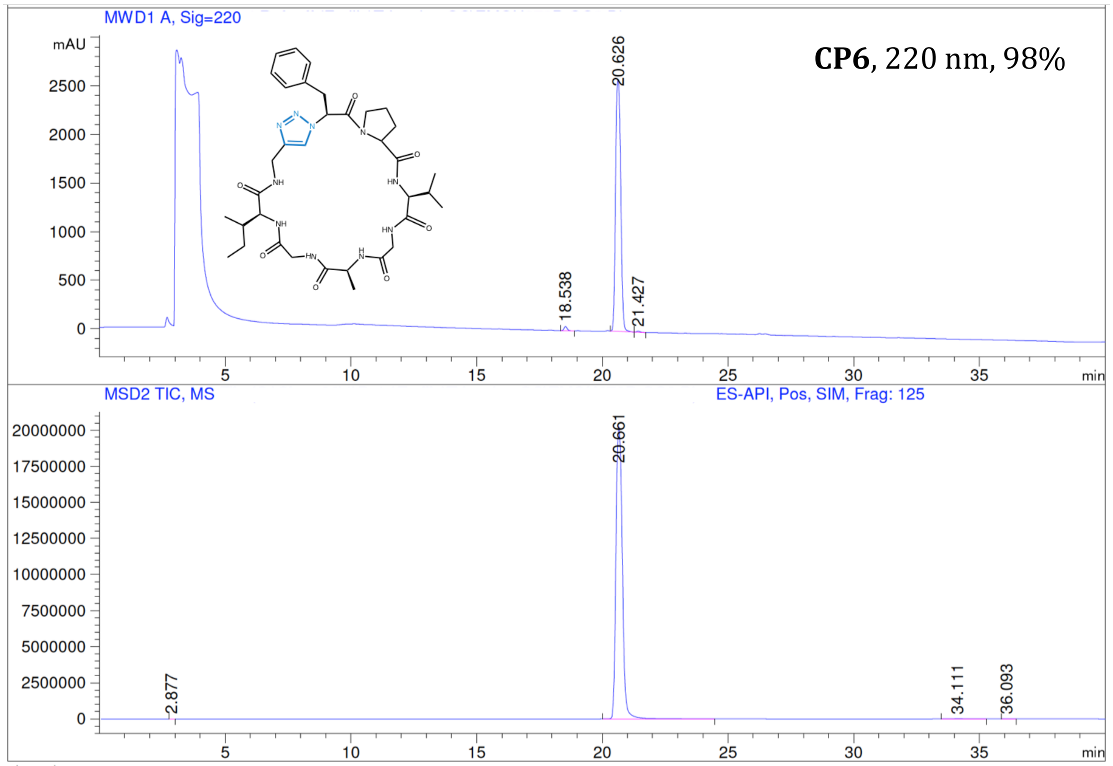

MWD1 A, Sig=220
CP6, 220 nm, 98%
mAU
2500
2000
1500
1000
500
0
20.626
18.538
21.427
5
10
15
20
25
30
35
min
MSD2 TIC, MS
ES-API, Pos, SIM, Frag: 125
20000000
17500000
15000000
12500000
10000000
7500000
5000000
2500000
0
20.661
2.877
34.111
36.093

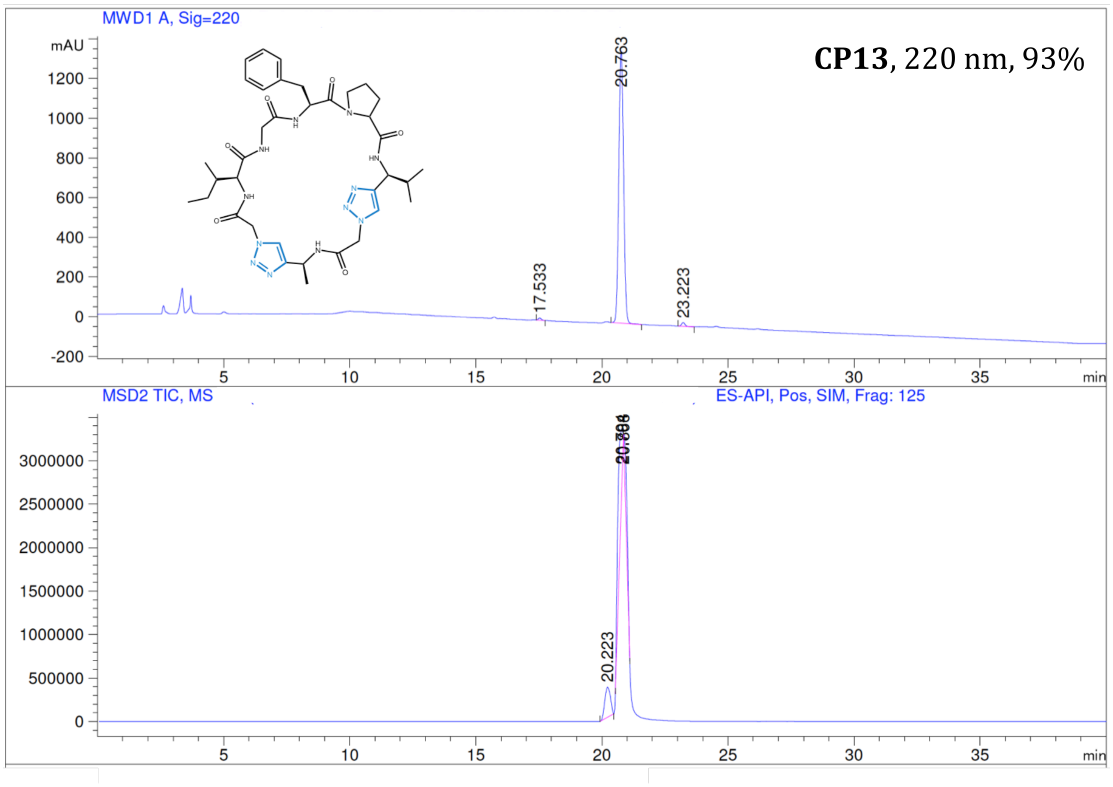

MWD1 A, Sig=220
CP13, 220 nm, 93%
mAU
1200
1000
800
600
400
200
0
-200
20.763
17.533
23.223
5
10
15
20
25
30
35
min
MSD2 TIC, MS
ES-API, Pos, SIM, Frag: 125
3000000
2500000
2000000
1500000
1000000
500000
0
20.223

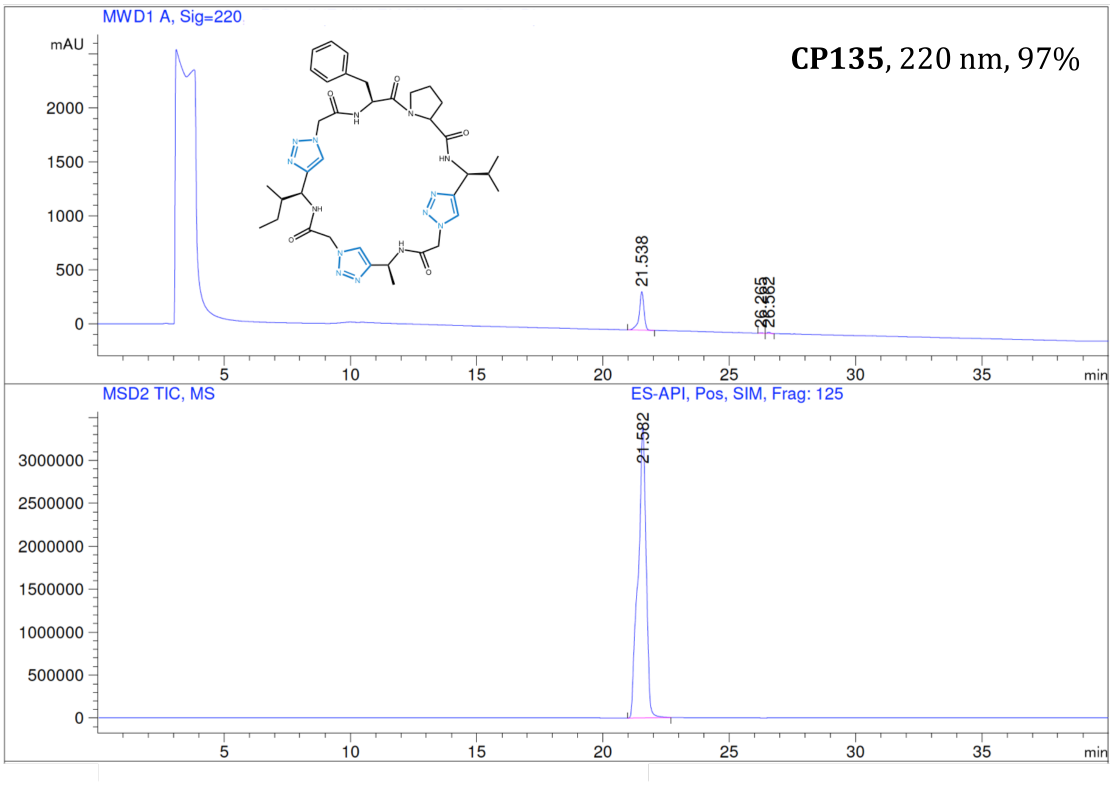

XI. LC-MS traces of starting peptides

For each of the precursor peptides **P,** the UV trace at 220 nm obtained by HPLC is complemented by its corresponding LCMS trace at the desired molecular weight (Single Ion Monitoring SIM mode). The HPLC methods used are described in section I.

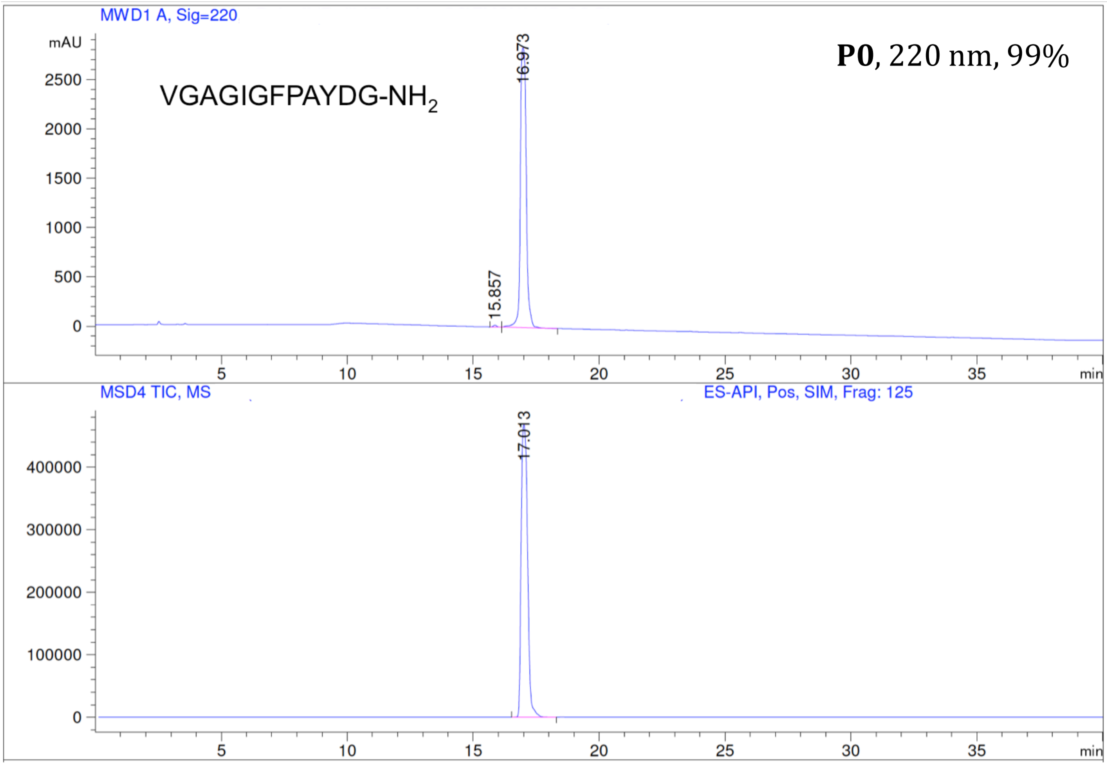

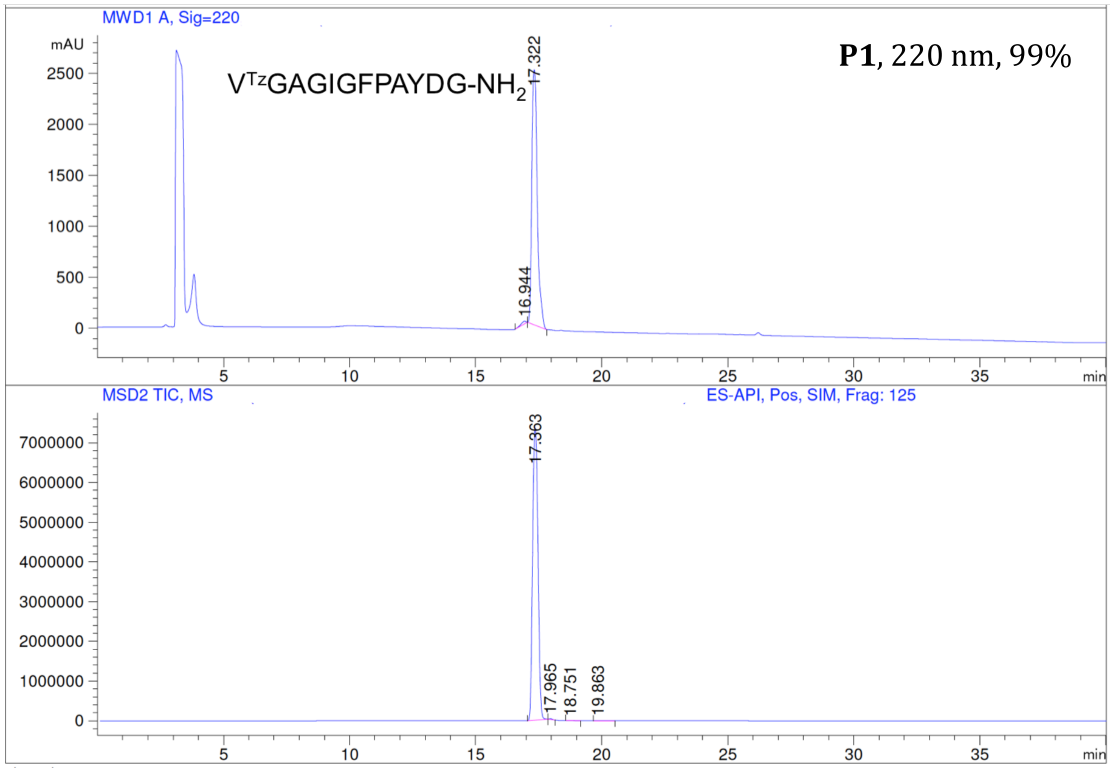

MWD1 A, Sig=220
mAU
2500
2000
1500
1000
500
0
VTzGAGIGFPAYDG-NH2
17.322
16.944
P1, 220 nm, 99%
5
10
15
20
25
30
35
min
MSD2 TIC, MS
ES-API, Pos, SIM, Frag: 125
7000000
6000000
5000000
4000000
3000000
2000000
1000000
0
17.363
17.965
18.751
19.863
5
10
15
20
25
30
35
min

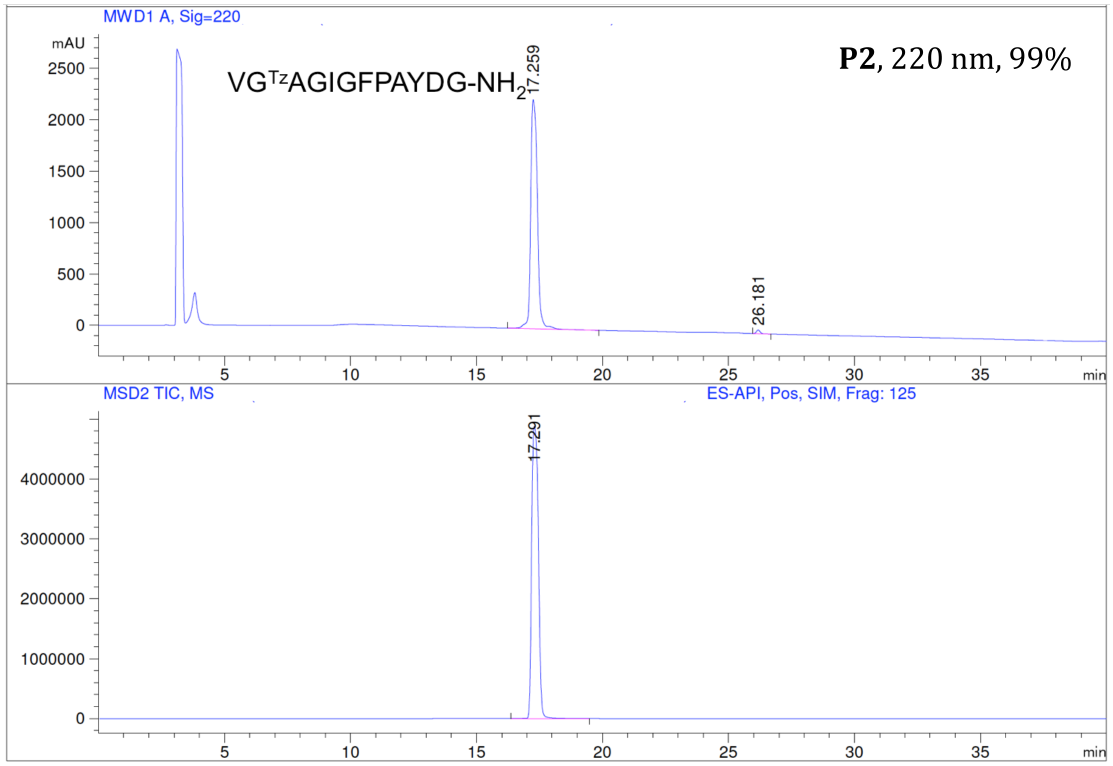

MWD1 A, Sig=220
mAU
2500
2000
1500
1000
500
0
VGTzAGIGFPAYDG-NH2
17.259
26.181
P2, 220 nm, 99%
5
10
15
20
25
30
35
min
MSD2 TIC, MS
ES-API, Pos, SIM, Frag: 125
4000000
3000000
2000000
1000000
0
17.291
5
10
15
20
25
30
35
min

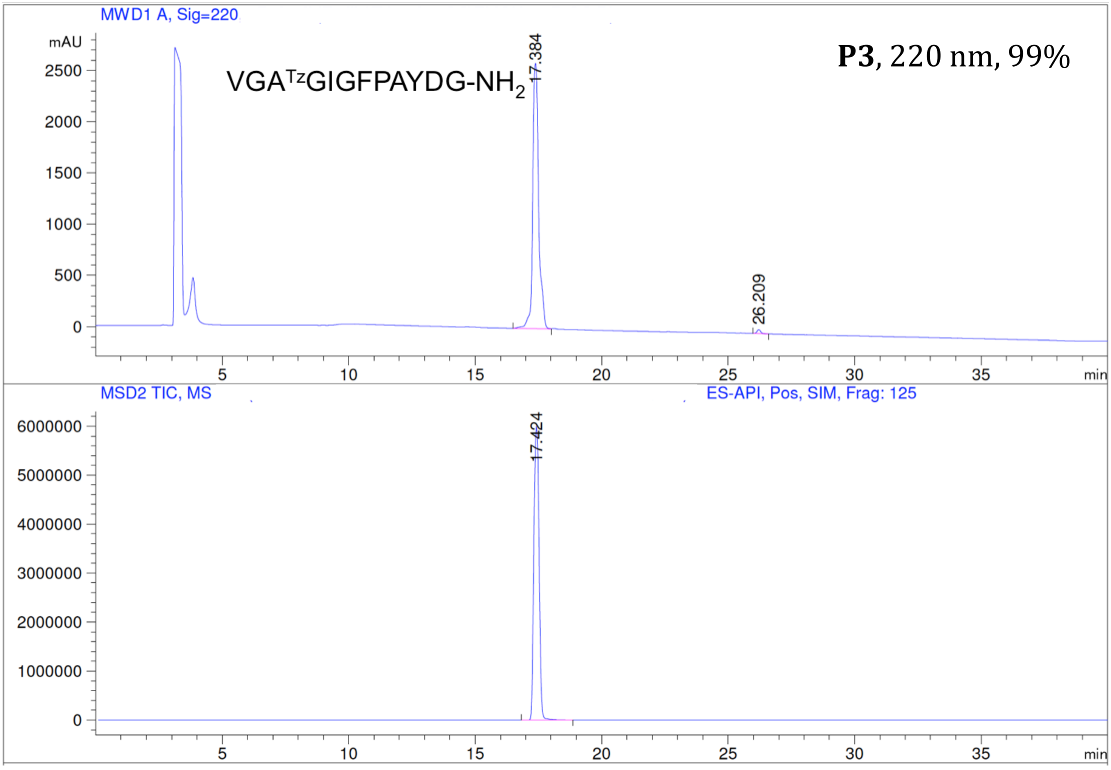

MWD1 A, Sig=220
mAU
P3, 220 nm, 99%
VGA^TzGIGFPAYDG-NH2
17.384
26.209
MSD2 TIC, MS
ES-API, Pos, SIM, Frag: 125
17.424
min

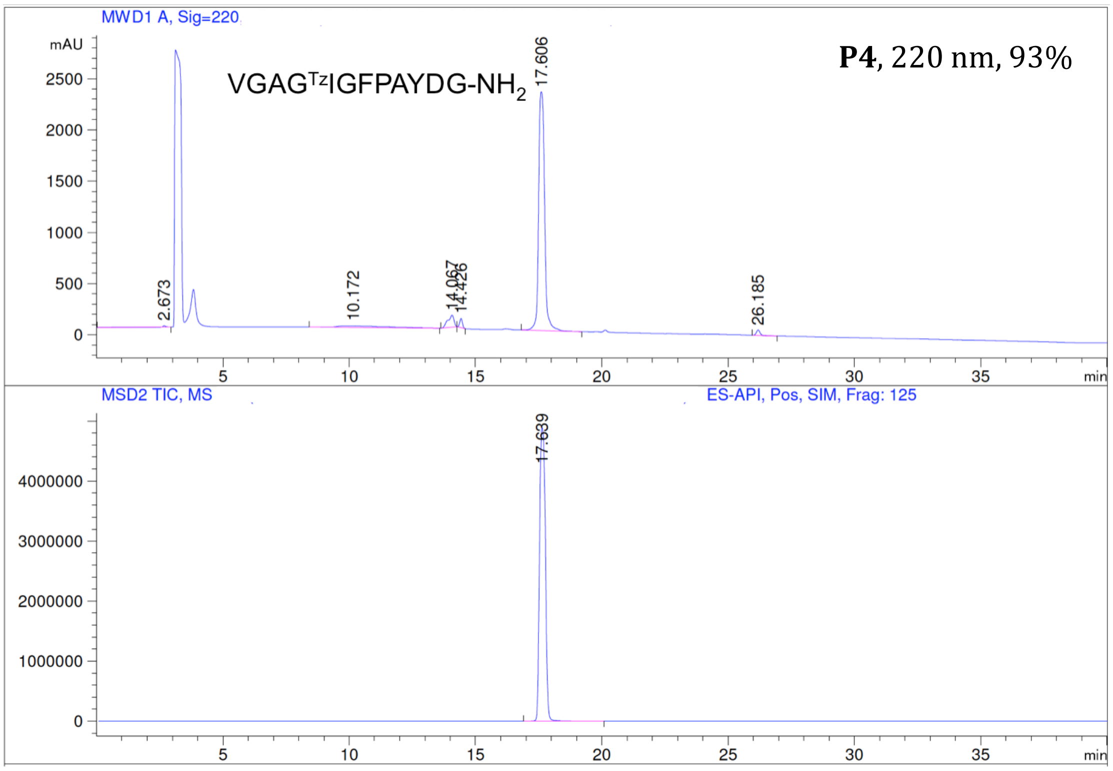

MWD1 A, Sig=220
mAU
P4, 220 nm, 93%
VGAG^TzIGFPAYDG-NH2
17.606
2.673
10.172
14.067
14.426
26.185
MSD2 TIC, MS
ES-API, Pos, SIM, Frag: 125
17.639
min

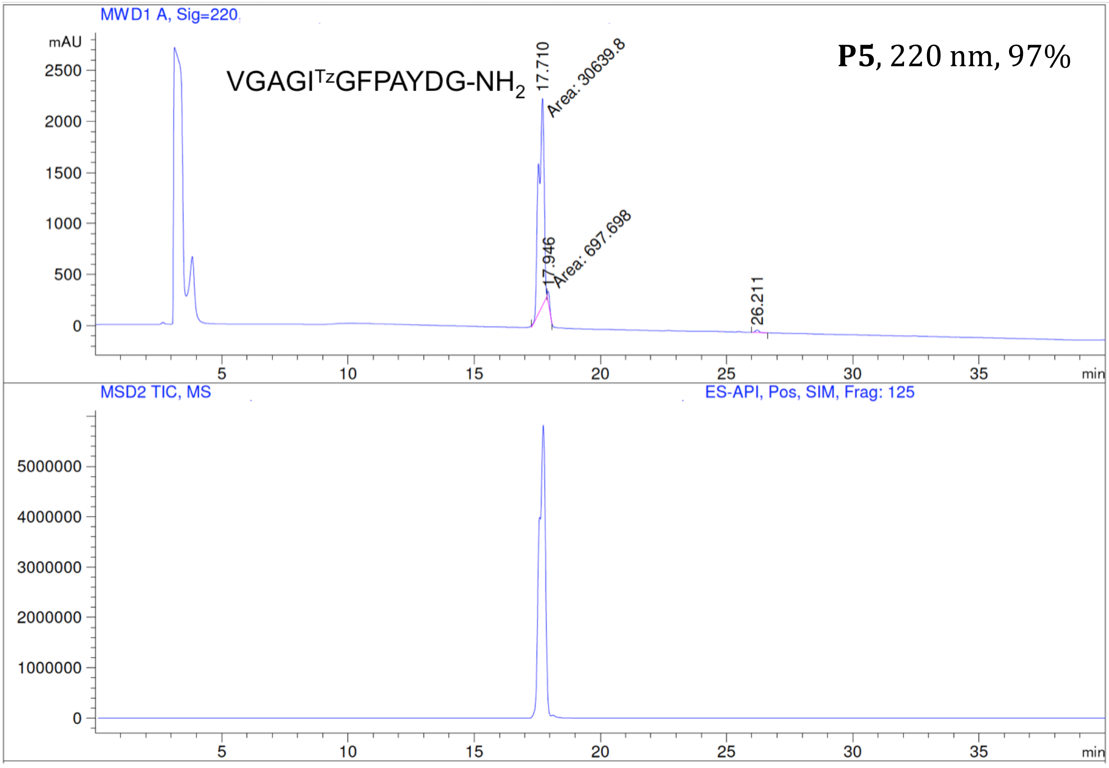
MWD1 A, Sig=220
mAU
VGAGI^{Tz}GFPAYDG-NH$_2$
P5, 220 nm, 97%
17.710
Area: 30639.8
17.946
Area: 697.698
26.211
MSD2 TIC, MS
ES-API, Pos, SIM, Frag: 125
min

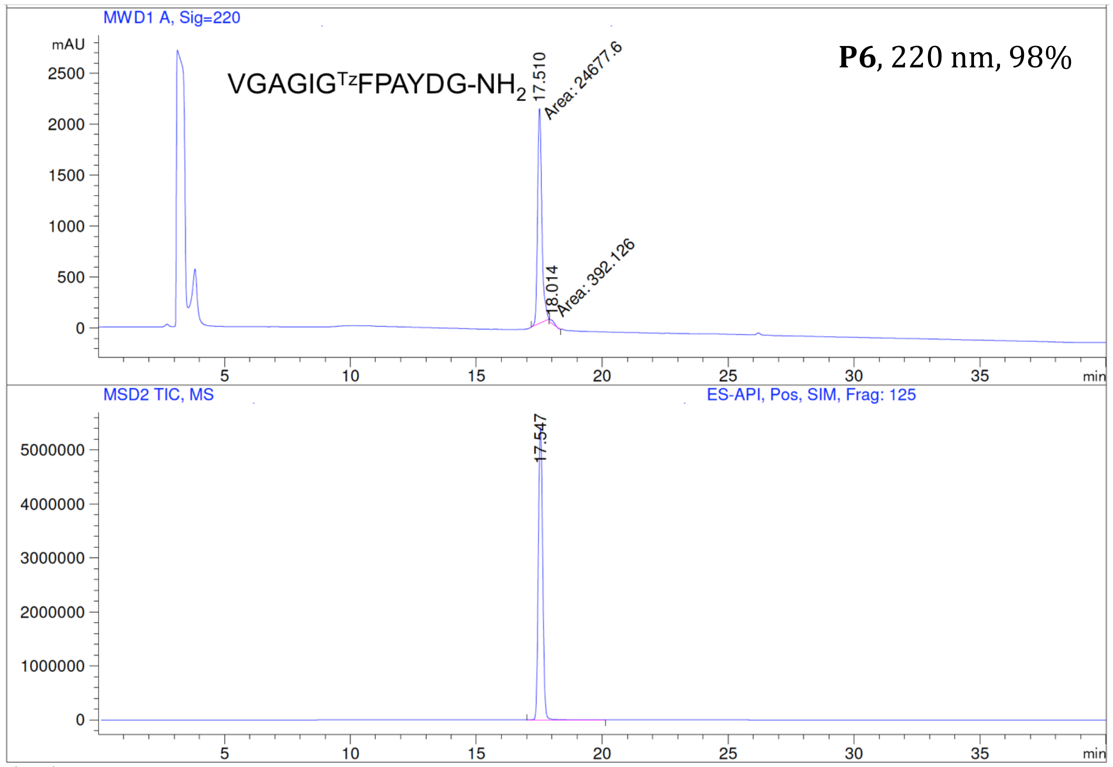
MWD1 A, Sig=220
mAU
VGAGIG^{Tz}FPAYDG-NH$_2$
P6, 220 nm, 98%
17.510
Area: 24677.6
18.014
Area: 392.126
MSD2 TIC, MS
ES-API, Pos, SIM, Frag: 125
17.547
min

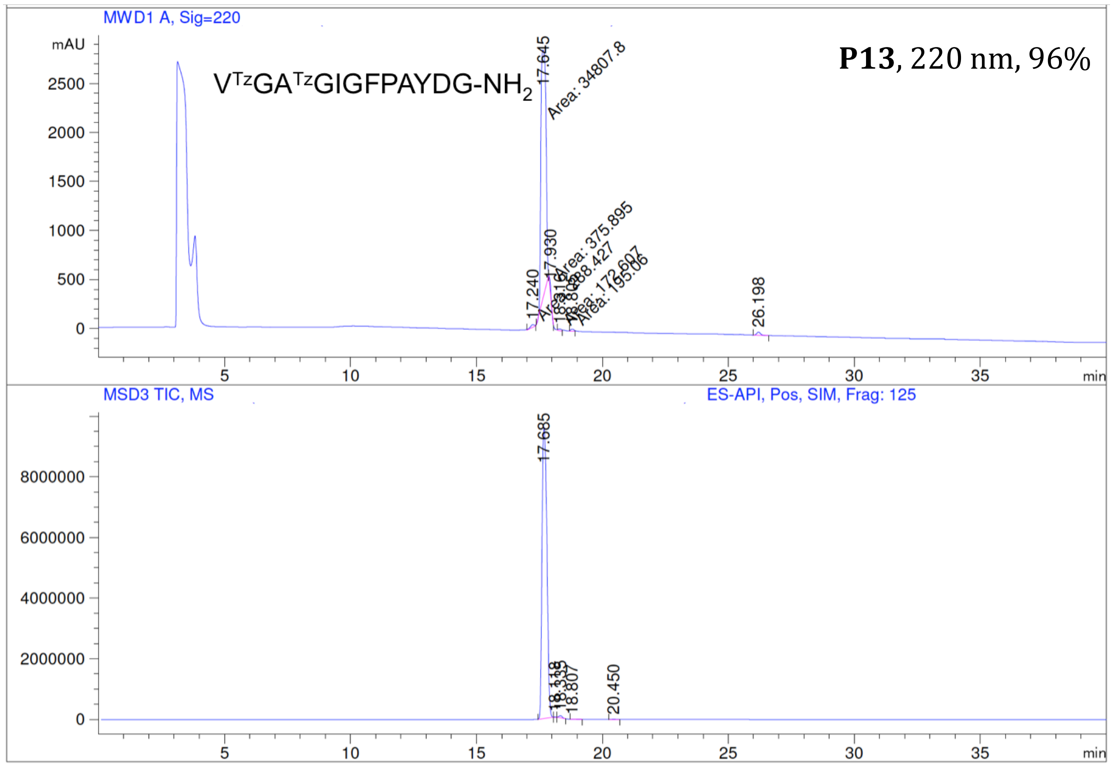
MWD1 A, Sig=220
mAU
V^TzGA^TzGIGFPAYDG-NH2
P13, 220 nm, 96%
17.645
Area: 34807.8
17.930
17.240
26.198
MSD3 TIC, MS
ES-API, Pos, SIM, Frag: 125
17.685
20.450
18.807
min

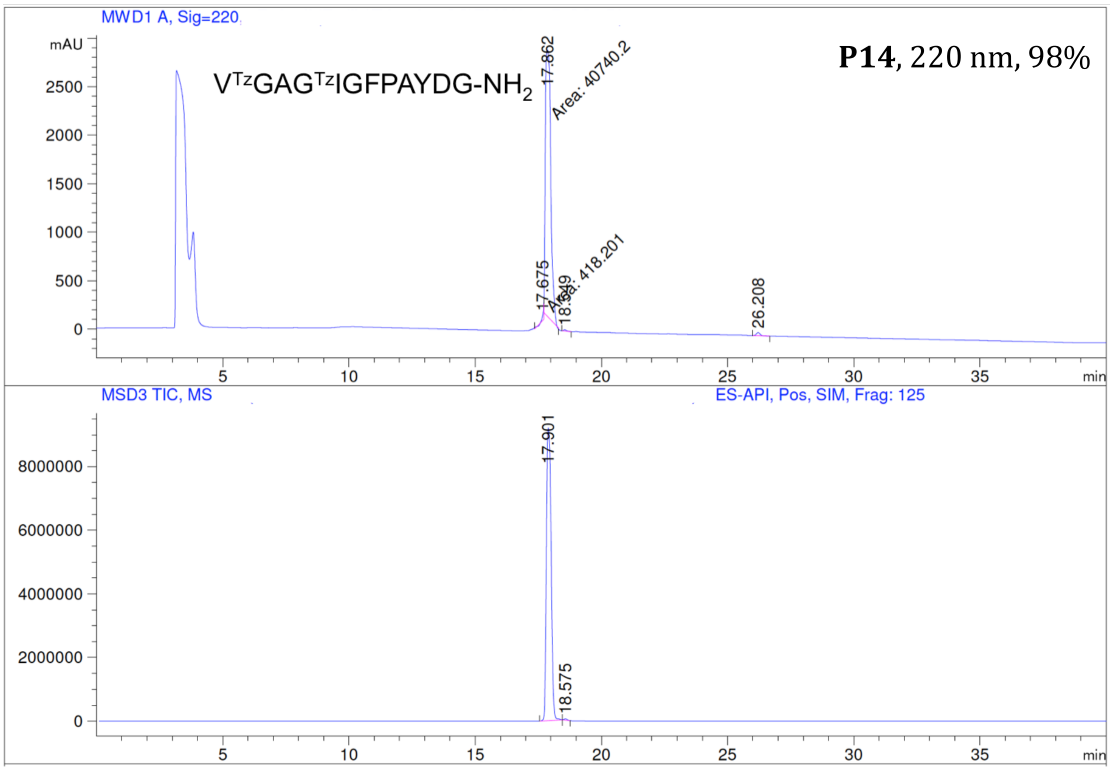
MWD1 A, Sig=220
mAU
V^TzGAG^TzIGFPAYDG-NH2
P14, 220 nm, 98%
17.862
Area: 40740.2
17.675
Area: 418.201
26.208
MSD3 TIC, MS
ES-API, Pos, SIM, Frag: 125
17.901
18.575
min

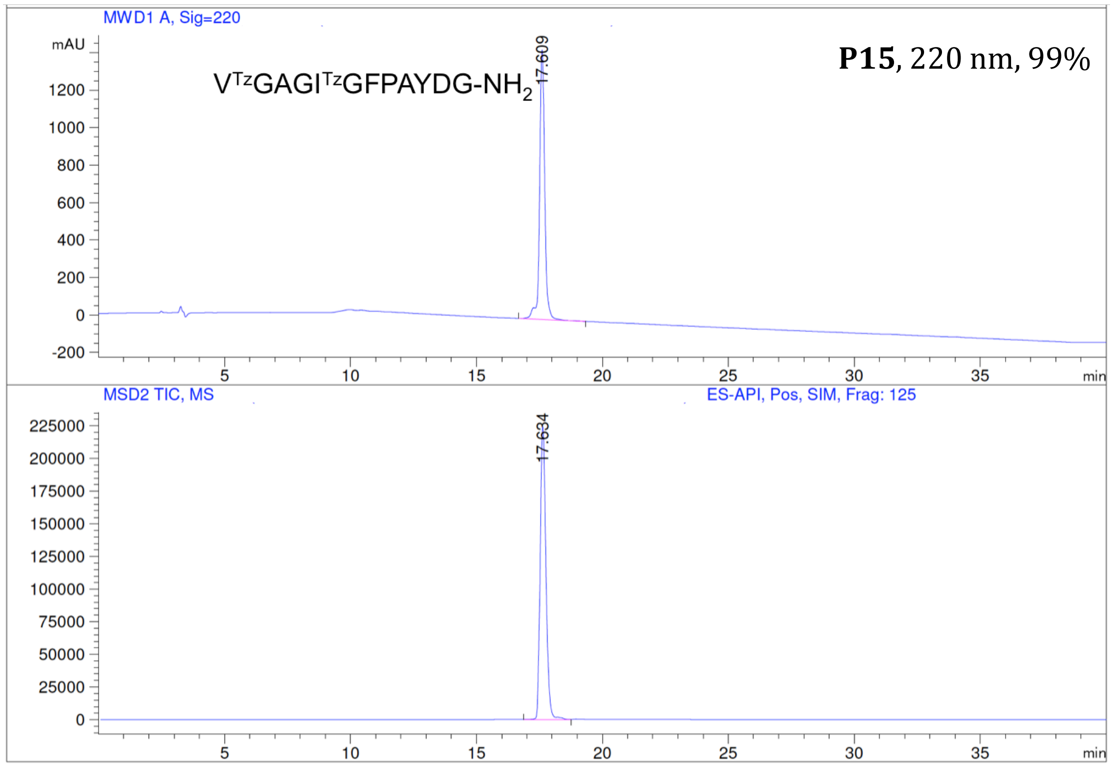

MWD1 A, Sig=220
mAU
P15, 220 nm, 99%
V^TzGAGI^TzGFPAYDG-NH2
17.609
MSD2 TIC, MS
ES-API, Pos, SIM, Frag: 125
17.634
min

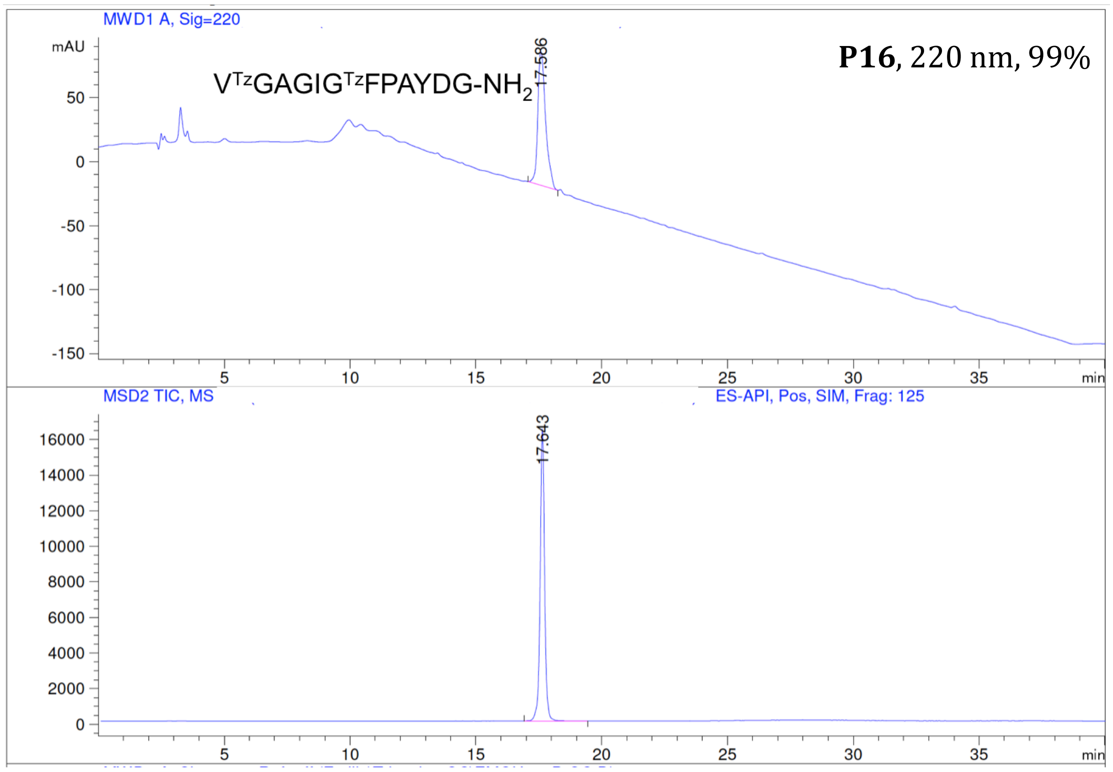

MWD1 A, Sig=220
mAU
P16, 220 nm, 99%
V^TzGAGIG^TzFPAYDG-NH2
17.586
MSD2 TIC, MS
ES-API, Pos, SIM, Frag: 125
17.643
min

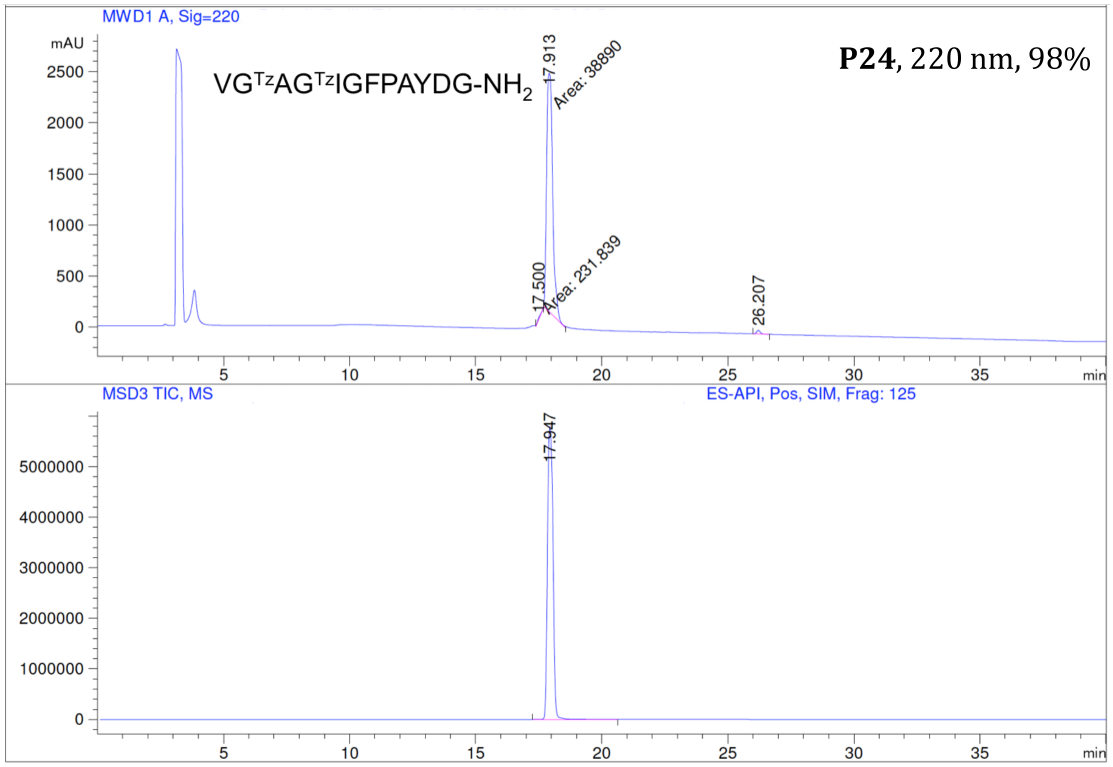
MWD1 A, Sig=220
mAU
VG^{Tz}AG^{Tz}IGFPAYDG-NH$_2$
P24, 220 nm, 98%
17.913
Area: 38890
17.500
Area: 231.839
26.207
min
MSD3 TIC, MS
ES-API, Pos, SIM, Frag: 125
17.947
min

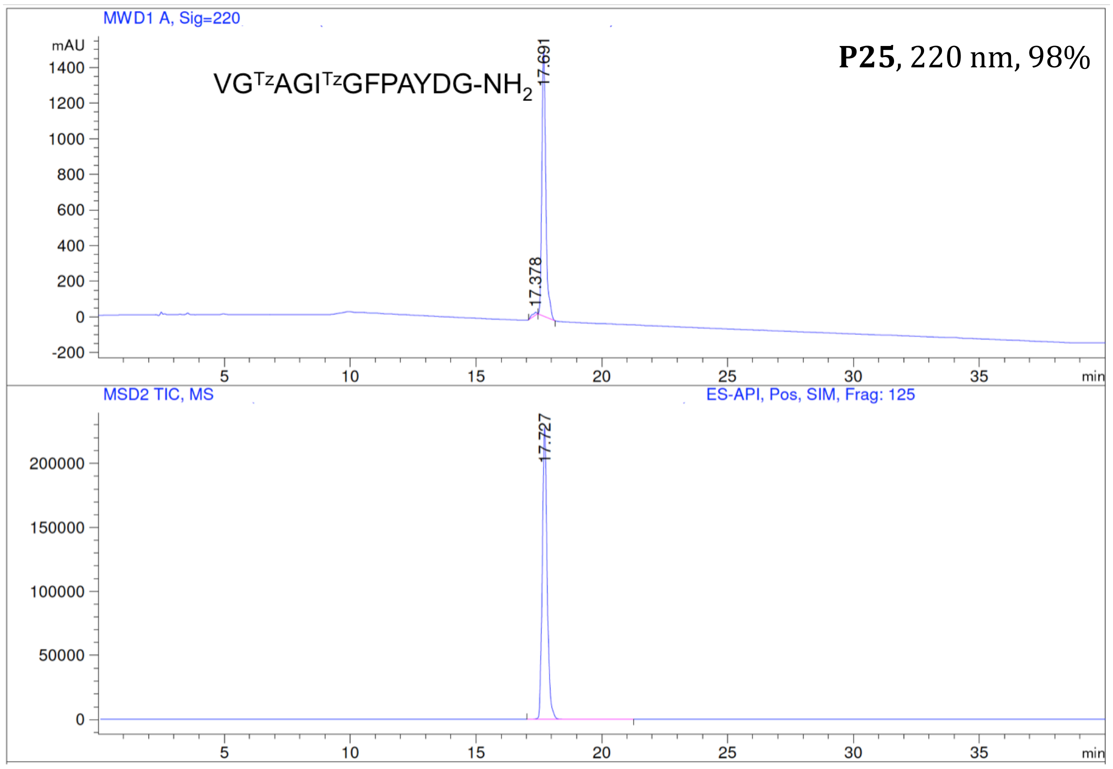
MWD1 A, Sig=220
mAU
VG^{Tz}AGI^{Tz}GFPAYDG-NH$_2$
P25, 220 nm, 98%
17.691
17.378
min
MSD2 TIC, MS
ES-API, Pos, SIM, Frag: 125
17.727
min

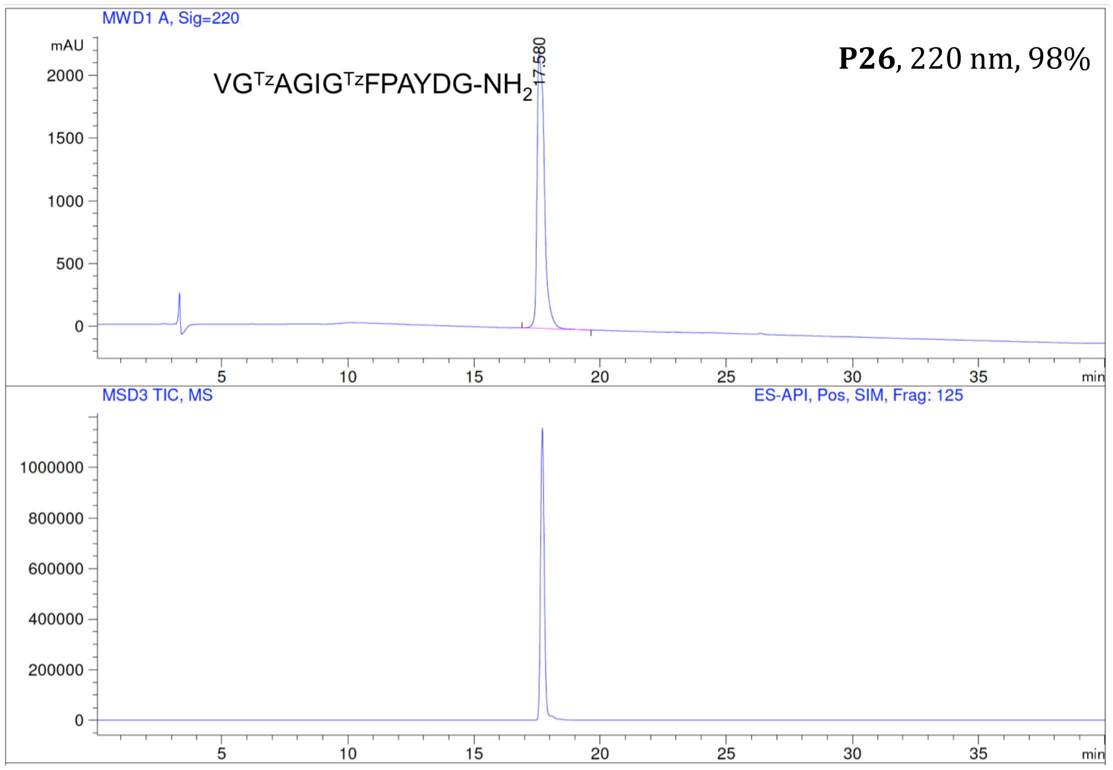
MWD1 A, Sig=220
mAU
VG^Tz AGIG^Tz FPAYDG-NH2
17.580
P26, 220 nm, 98%
MSD3 TIC, MS
ES-API, Pos, SIM, Frag: 125
min

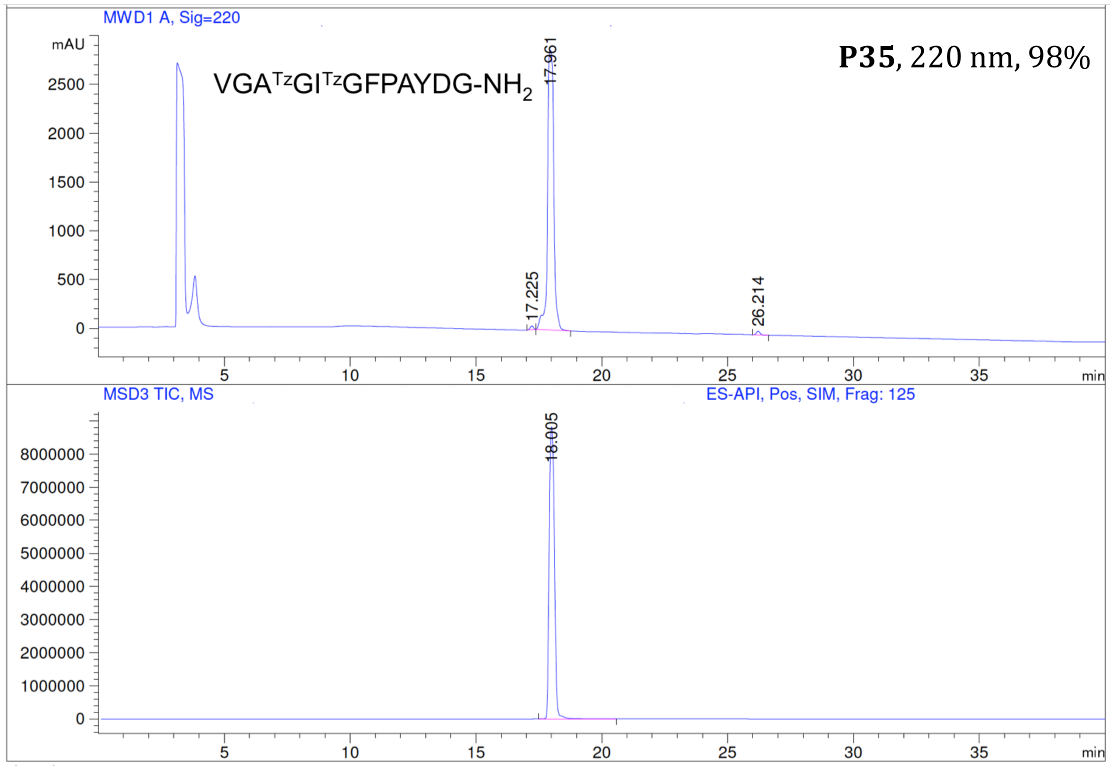
MWD1 A, Sig=220
mAU
VGA^Tz GI^Tz GFPAYDG-NH2
17.961
17.225
26.214
P35, 220 nm, 98%
MSD3 TIC, MS
ES-API, Pos, SIM, Frag: 125
18.005
min

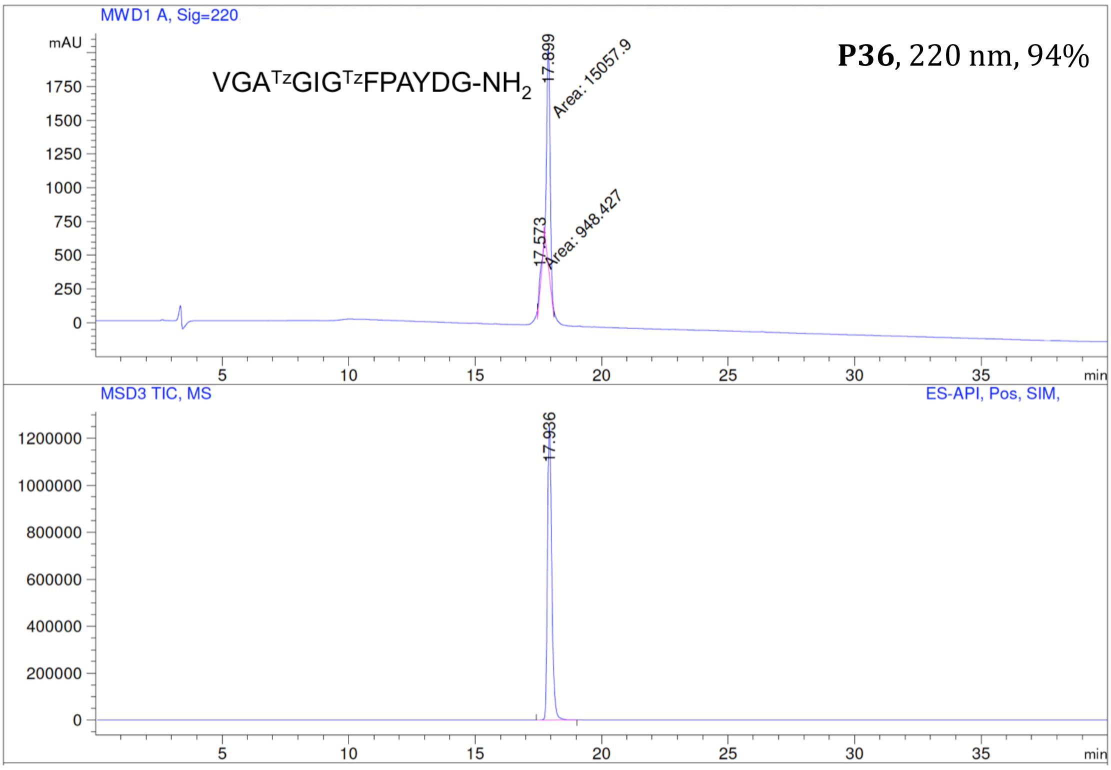
MWD1 A, Sig=220
mAU
1750
1500
1250
1000
750
500
250
0
VGA^{Tz}GIG^{Tz}FPAYDG-NH2
17.899
Area: 15057.9
17.573
Area: 948.427
P36, 220 nm, 94%
5
10
15
20
25
30
35
min
MSD3 TIC, MS
ES-API, Pos, SIM,
1200000
1000000
800000
600000
400000
200000
0
17.936

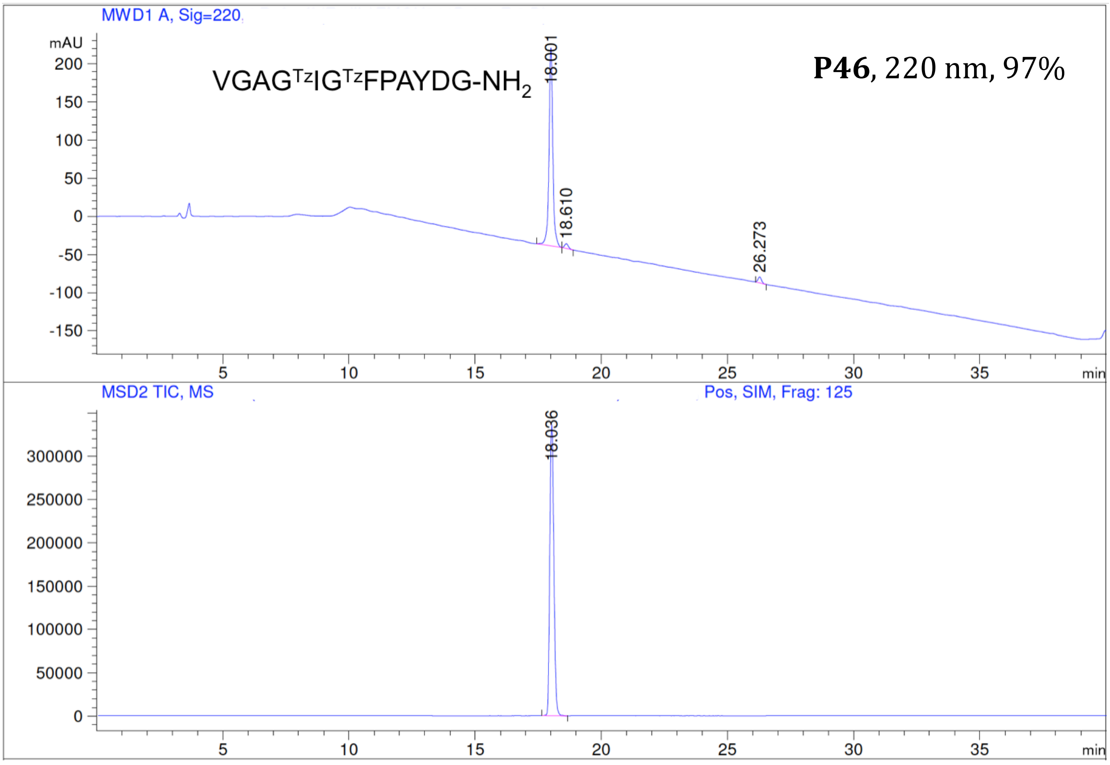
MWD1 A, Sig=220
mAU
200
150
100
50
0
-50
-100
-150
VGAG^{Tz}IG^{Tz}FPAYDG-NH2
18.001
18.610
26.273
P46, 220 nm, 97%
5
10
15
20
25
30
35
min
MSD2 TIC, MS
Pos, SIM, Frag: 125
300000
250000
200000
150000
100000
50000
0
18.036

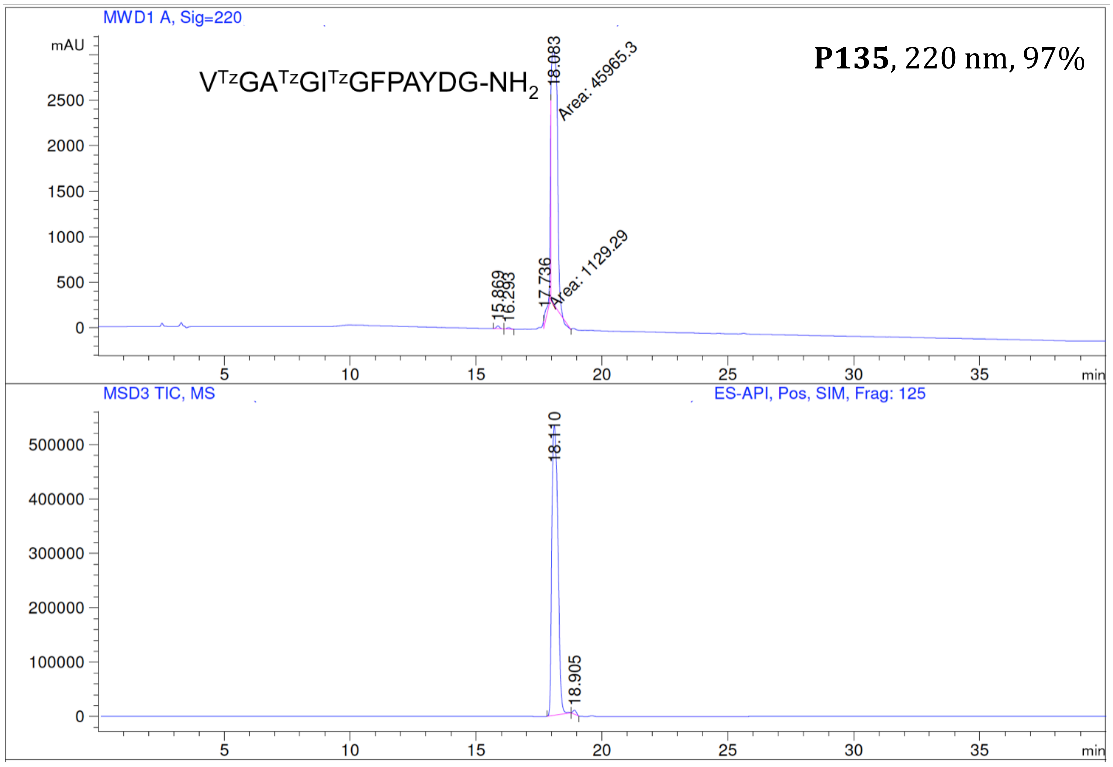
MWD1 A, Sig=220
mAU
P135, 220 nm, 97%
V^Tz^GA^Tz^GI^Tz^GFPAYDG-NH2
18.083
Area: 45965.3
15.869
16.293
17.736
Area: 1129.29
MSD3 TIC, MS
ES-API, Pos, SIM, Frag: 125
18.110
18.905
min

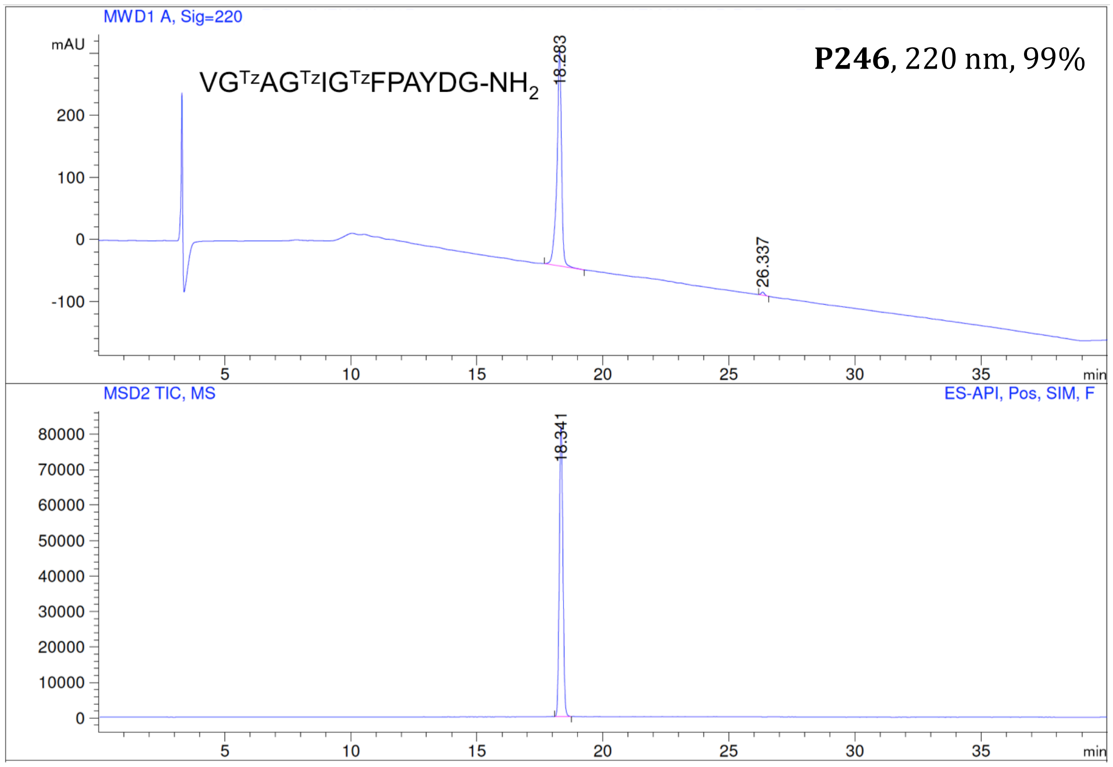
MWD1 A, Sig=220
mAU
P246, 220 nm, 99%
VG^Tz^AG^Tz^IG^Tz^FPAYDG-NH2
18.283
26.337
MSD2 TIC, MS
ES-API, Pos, SIM, F
18.341
min

XII. References

[1] G. R. Fulmer, A. J. M. Miller, N. H. Sherden, H. E. Gottlieb, A. Nudelman, B. M. Stoltz, J. E. Bercaw, K. I. Goldberg, *Organometallics* **2010**, *29*, 2176.

[2] J. Koehnke, A. Bent, W. E. Houssen, D. Zollman, F. Morawitz, S. Shirran, J. Vendome, A. F. Nneoyiegbe, L. Trembleau, C. H. Botting, M. C. M. Smith, M. Jaspars, J. H. Naismith, *Nat Struct Mol Biol* **2012**, *19*, 767.

[3] E. D. Goddard-Borger, R. V. Stick, *Organic Letters* **2007**, *9*, 3797.

[4] N. Fischer, E. D. Goddard-Borger, R. Greiner, T. M. Klapötke, B. W. Skelton, J. Stierstorfer, *The Journal of Organic Chemistry* **2012**, *77*, 1760-1764.

[5] Lundquist, J. C. Pelletier, *Organic Letters* **2001**, *3*, 781.

[6] H. D. Dickson, S. C. Smith, K. W. Hinkle, *Tetrahedron Letters* **2004**, *45*, 5597-5599.

[7] J.-A. Fehrentz, B. Castro, *Synthesis* **1983**, *1983*, 676.

[8] G. Reginato, A. Mordini, F. Messina, A. Degl'Innocenti, G. Poli, *Tetrahedron* **1996**, *52*, 10985.

[9] I. E. Valverde, A. Bauman, C. A. Kluba, S. Vomstein, M. A. Walter, T. L. Mindt, *Angew. Chem. Int. Ed.* **2013**, *52*, 8957.

[10] M. Tischler, D. Nasu, M. Empting, S. Schmelz, D. W. Heinz, P. Rottmann, H. Kolmar, G. Buntkowsky, D. Tietze, O. Avrutina, *Angew. Chem. Int. Ed.* **2012**, *51*, 3708.

[11] A. Tam, U. Arnold, M. B. Soellner, R. T. Raines, *J. Am. Chem. Soc.* **2007**, *129*, 12670.
